# Supply Constraint from Earthquakes in Japan in Input–Output Analysis

**DOI:** 10.1111/risa.13525

**Published:** 2020-06-07

**Authors:** Michiyuki Yagi, Shigemi Kagawa, Shunsuke Managi, Hidemichi Fujii, Dabo Guan

**Affiliations:** ^1^ Faculty of Economics and Law Shinshu University 3‐1‐1 Asahi Matsumoto Nagano 390‐8621 Japan; ^2^ Faculty of Economics Kyushu University 744 Motooka Nishi‐ku Fukuoka 819‐0395 Japan; ^3^ Urban Institute & Department of Civil Engineering Kyushu University 744 Motooka Nishi‐ku Fukuoka 819‐0395 Japan; ^4^ Department of Earth System Science Tsinghua University Room S801, S803, S805 Mengminwei Science and Technology Building Haidian Beijing 100084 China

**Keywords:** Earthquakes in Japan, input‐output analysis, supply constraint

## Abstract

Disasters often cause exogenous flow damage (i.e., the [hypothetical] difference in economic scale with and without a disaster in a certain period) to production (“supply constraint”). However, input‐output (IO) analysis (IOA) cannot usually consider it, because the Leontief quantity model (LQM) assumes that production is endogenous; the Ghosh quantity model (GQM) is considered implausible; and the Leontief price model (LPM) and the Ghosh price model (GPM) assume that quantity is fixed. This study proposes to consider a supply constraint in the LPM, introducing the price elasticity of demand. This study uses the loss of social surplus (SS) as a damage estimation because production (sales) is less informative as a damage index than profit (margin); that is, production can be any amount if without considering profit, and it does not tell exactly how much profit is lost for each supplier (upstream sector) and buyer (downstream sector). As a model application, this study examines Japan's largest five earthquakes from 1995 to 2017 and the Great East Japan Earthquake (GEJE) in March 2011. The worst earthquake at the peak tends to increase price by 10–20% and decrease SS by 20–30%, when compared with the initial month's prices/production. The worst damage tends to last eight months at most, accumulating 0.5‐month‐production damage (i.e., the sum of [hypothetical] differences in SS with and without an earthquake [for eight months] is 50% of the initial month production). Meanwhile, the GEJE in the five prefectures had cumulatively, a 25‐month‐production damage until the temporal recovery at the 37th month.

## 1 INTRODUCTION

Japan is known as an earthquake‐prone country; between 1996 and September 2018, there were 155 earthquakes—an average of 6.7 earthquakes per year—which resulted in human injuries (Japan Meteorological Agency, 2018). Dead or missing people were reported as a result of 20 of these 155 earthquakes, with more than 10 people being reported dead or missing as a result of six of them. Ninety‐nine cases resulted not only in human injuries but also in physical damage (houses, school buildings, landslides, window glass, water pipes, and so on). Tsunamis occurred in 18 cases, and more than 1 meter tsunamis occurred in three cases (the mortality rate when a person is involved in a 1 m tsunami is almost 100%). Note that in 1995, the Hyogo‐ken Nanbu Earthquake (the so‐called Great Hanshin Earthquake) resulted in 6,434 deaths and three missing people.

Natural disasters such as earthquakes often disrupt economic activities across supply chains. To effectively use human capital and efficiently transform materials, production supply chains have become more complex (Brown, 2015). The risks of supply chain disruption have also increased because of unplanned and unusual events within complex production supply chains (Mital, Del Giudice, & Papa, 2018). Savitz (2012) used the Great East Japan Earthquake (GEJE, Japan) in March 2011 and the major flooding in Thailand in 2011 to highlight the need for manufacturers to apply effective risk‐management strategies along supply chains to minimize disruptions. These examples demonstrate the importance of forecasting the economic damage that will result from supply chain disruptions when structuring risk‐management schemes for product supply chains.

Economic methods such as computable general equilibrium (CGE) analysis, econometrics, and input–output (IO) analysis (IOA) have been widely used to quantify the economic damage associated with natural disasters, including earthquakes. They have also been used to conduct pre‐ and postevaluations of recoveries from economic damage. Among them, IOA can be effective in evaluating economic impacts at the regional/sectoral level through the reduction in intermediate demand. In recent years, studies have used IOA to quantify the environmental loads with respect to greenhouse gas emissions (Kanemoto, Moran, & Hertwich, 2016), water consumption (Feng, Chapagain, Suh, Pfister, & Hubacek, 2011), land use change (Weinzettel, Hertwich, Peters, Steen‐Olsen, & Galli, 2013), biodiversity trends (Wilting, Schipper, Bakkenes, Meijer, & Huijbregts, 2017), and material consumption (Wiedmann et al., 2015), for example. These loads are included in “environmental footprint (Hoekstra & Wiedmann, 2014).” Although no extant method is versatile enough to evaluate economic damage over the long and short term, IOA is often used to analyze economic damage incurred through the supply chain as a result of large‐scale, regional disasters such as floods and hurricanes (Crawford‐Brown et al., 2013; Hallegatte, 2008; Li, Crawford‐Brown, Syddall, & Guan, 2013; Okuyama, 2007; Shimoda & Fujikawa, 2012).

**Fig 1:**
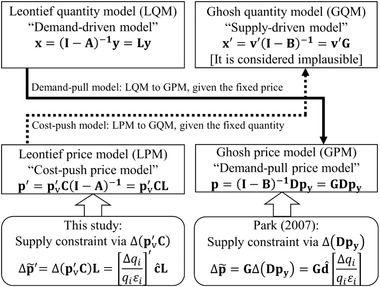
Leontief and Ghosh models with Park (2007) and this study. *Notes*: This figure shows the relationship between the four models (LQM, GQM, LPM, and GPM) and the application of supply constraint by Park (2007) and this study. As the demand‐pull model, LQM can be converted to GPM, given the price is fixed. Meanwhile, as the cost‐push model, LPM can be converted to GQM, given the quantity is fixed. The supply constraints are considered via GPM in Park (2007) and LPM in this study.

This study focuses on an exogenous (flow) damage (i.e., the hypothetical difference of economic scales with and without a disaster in a certain period) to production (“supply constraint”) in IOA. The reason for such focus is because we can often know changes in industrial production at the monthly or quarterly levels from specific production statistics (provided by governments, industry organizations, and so on). As an issue, however, IOA cannot basically handle the supply constraint. Four typical IO models are the Leontief quantity model (LQM; Leontief, 1936), the Ghosh quantity model (GQM; Ghosh, 1958), the Leontief price model (LPM), and the Ghosh price model (GPM) (note that these price models were independently developed by Davar (1989) and Oosterhaven (1989)) (Fig. 1). The LQM cannot treat the supply constraint because production is endogenous. In GQM, although value added (or primary input) is exogenous, GQM itself is considered implausible (Oosterhaven, 1988, 1989, 1996, 2012). In LPM and GPM, because the quantity is assumed to be fixed, the supply constraint is not basically applicable.

The purpose of this study is to propose a method that can consider the supply constraint in LPM, introducing the price elasticity of demand. This idea comes from Park (2007), who uniquely interpreted that GPM can consider the supply constraint. This approach requires the common assumptions of IOA, for instance, that the technical coefficient is fixed.

This study modifies Park's (2007) approach in certain aspects. Specifically, this study proposes using the loss of social surplus (SS) as damage instead of the change in production. Production (sales) is less informative as a damage index than profit (margin) because it can be any amount without considering profit. Also, production does not exactly tell how much profit is lost for or how much damage of the supply constraint is passed on to each supplier (upstream sectors) and each buyer (downstream sectors). For example, suppose the supply quantity is constrained to 80% after the disaster, increasing the price to 125%. Thus, production remains 100%, but the buyers are damaged because they buy smaller quantities at the higher price. Such damage information is available in SS, which is consumer surplus (CS) plus producer surplus (PS).

As a model application, among the 155 earthquakes plus the Hyogo‐ken Nanbu Earthquake in 1995, which is included because of the huge impact that earthquake had, this study examines the six largest earthquakes in terms of deaths and missing people (Table I). These earthquakes are: the Hyogo‐ken Nanbu Earthquake in January 1995 (hereafter H95Jan), which resulted in 6,434 deaths and three missing people; the Mid Niigata Prefecture Earthquake in October 2004 (N04Oct), with 68 deaths; the Niigata‐ken Chuetsu‐oki Earthquake in July 2007 (N07Jul), with 15 deaths; the Iwate‐Miyagi Nairiku Earthquake in June 2008 (IM08Jun), with 17 deaths and six missing people; the GEJE, with 19,630 deaths and 2,569 missing people; and the 2016 Kumamoto earthquakes in April 2016 (K16Apr), which resulted in 269 deaths.

**Table I:** Japan's six most destructive earthquakes between 1995 and 2017 and the research settings

#	Date of occurrence	Earthquake name (location)	Human damage	Main Physical damage	Focal prefectures	*t* = 0	*t* = 1
H95Jan	Jan 17, 1995	The Hyogo–ken Nanbu Earthquake (Hanshin region; mainly in Hyogo)	6,434 deaths, 3 missing people, and 43,792 injured people	104,906 and 144,274 houses were completely and partially destroyed	Hyogo	(May 1995)	January 1995
N04Oct	October 23, 2004	The Mid Niigata Prefecture Earthquake in 2004 (Chuetsu [middle] region of Niigata)	68 deaths and 4,805 injured people	3,175 and 13,810 houses were completely and partially destroyed	Niigata	September 2004	October 2004
N07Jul	July 16, 2007	The Niigata‐ken Chuetsu‐oki Earthquake in 2007 (Chuetsu offshore of Niigata)	15 deaths and 2,346 injured people	1,331 and 5,710 houses were completely and partially destroyed	Niigata	June 2007	July 2007
IM08Jun	June 14, 2008	The Iwate‐Miyagi Nairiku Earthquake in 2008 (southern inland of Iwate Prefecture)	17 deaths, 6 missing people and 426 injured people	30 and 146 houses were completely and partially destroyed	Iwate and Miyagi	May 2008	June 2008
GEJE	March 11, 2011	The 2011 off the Pacific coast of Tohoku Earthquake (the Great East Japan Earthquake)	19,630 deaths, 2,569 missing people, and 6,230 injured people	121,781 and 280,962 houses were completely and partially destroyed	Fukushima, Iwate, Miyagi, Ibaraki, and Chiba (FIMIC)	Mar 2016	April 2016
K16Apr	April 14 to 16, 2016	The 2016 Kumamoto earthquakes (Kumamoto prefecture)	269 deaths and 2,806 injured people	8,668 and 34,716 houses were completely and partially destroyed	Kumamoto	February 2011	March 2011

*Notes*: Regarding H95Jan, because there is no data for the month prior to this earthquake (i.e., December 1994), the month before the disaster (*t* = 0) is set as May 1995 because this month exhibited the highest production capacity in that year.

The structure of this article is as follows. Section 2 explains the basic IO models. Section 3 models the supply constraint in IOA, following and modifying Park (2007). Section 4 explains the application to earthquakes in Japan. Section 5 shows the estimated results, and Section 6 concludes.

## 2 BASIC IO MODELS

### 2.1 Basic Quantity and Price IO Models

Two basic IO models (following Miller & Blair, 2009; and Oosterhaven, 1996) are the Leontief model (LQM and LPM) and the Ghosh model (GQM and GPM) (Fig. 1). Note that to facilitate understanding by comparison, this section follows the explanation in Oosterhaven (1996). LQM (“the demand‐driven model”) is expressed as:

(1) x=Zi+Yi=Zi+y=Ax+y.

**x** is *I*‐vector total output (or input) per sector: $\left[x_{i}\right]$ where brackets represent a vector or matrix. *I* means the number of sectors. **Z** is *I × I*‐matrix with intermediate outputs (or inputs) per sector: $\left[z_{ij}\right]$. **Y** is *I × M*‐matrix with final demand (or outputs) per sector: $\left[y_{im}\right]$. *M* means the number of categories in final demand. **i** is a summation vector (of one). **A** is *I × I*‐matrix with fixed intermediate input coefficients (or technical input coefficients) $\left(\left[a_{ij}\right]\right)$ in a single‐region IOA. Note that in a multiregional IO (MRIO) model, sectors *i* and *j* may be in different regions, trading a good from *i* to *j*. Thus, the MRIO model terms $a_{ij}$ not as a “technical coefficient” but as “the product of a technical IO coefficient and IO trade coefficient” (Oosterhaven & Hewings, 2014).

(2) $$Z=A\hat{x}\Leftrightarrow A=Z{\hat{x}}^{-1},$$

where the hat (of **x**) means a diagonal matrix. LQM is solved:

(3) $$x={\left(I-A\right)}^{-1}y=\mathrm{Ly},$$

where **I** is the *I × I*‐identity matrix. **L** is the so‐called Leontief inverse (or input inverse), where $L=\left[l_{ij}\right]={\left(I-A\right)}^{-1}$.

LPM (“the cost‐push price model”; Davar, 1989; Oosterhaven, 1989) is expressed as:

(4) $$p^{\prime }=p^{\prime }A+p_{v}^{\prime }C.$$

**p** is an *I*‐vector of index price [*p_i_*] for sectoral output (unit cost for purchasers). **p_v_** is an *N*‐vector [*p_vn_*] of index price for primary inputs (or value added). **p** and **p_v_** are usually a unit vector (i.e., one) (however, the lengths I and N are different). **C** is *N × I* matrix of fixed primary input coefficients, where **V** is the *N × I* matrix of primary inputs (value added, [*v_ni_*]). *N* means the number of categories in value added.

(5) $$V=C\hat{x}\Leftrightarrow C=V{\hat{x}}^{-1}.$$

The sum of weights regarding **A** and **C** is equal to 1 ($i^{\prime }A+i^{\prime }C=i^{\prime }$). LPM is solved:

(6) $$p^{\prime }=p_{v}^{\prime }C{\left(I-A\right)}^{-1}=p_{v}^{\prime }\mathrm{CL}.$$

Meanwhile, GQM (“the supply‐driven model”) is expressed as:

(7) $$x^{\prime }=i^{\prime }Z+i^{\prime }V=i^{\prime }Z+v^{\prime }=x^{\prime }B+v^{\prime }.$$

**B** is the *I × I*‐matrix, with fixed intermediate output coefficients (or technical output coefficients) ($\left[b_{ij}\right]$) in a single‐region IOA. Similarly to LQM, an MRIO model terms $b_{ij}$ not as a “technical coefficient” but as “the product of technical IO coefficient and IO trade coefficient” (Oosterhaven & Hewings, 2014).

(8) $$Z=\hat{x}B\Leftrightarrow B={\hat{x}}^{-1}Z.$$

The relationship between **A** and **B** is expressed as:

(9) $$B={\hat{x}}^{-1}A\hat{x}\Leftrightarrow A=\hat{x}B{\hat{x}}^{-1}.$$

GQM is solved:

(10) $$x^{\prime }=v^{\prime }{\left(I-B\right)}^{-1}=v^{\prime }G.$$

**G** is called the Ghosh inverse (or output inverse), where $G=\left[g_{ij}\right]={\left(I-B\right)}^{-1}$.

GQM is mathematically equivalent to LPM, given that price is variable and quantity is fixed (Dietzenbacher, 1997). Following Park (2007), recall that $B={\hat{x}}^{-1}A\hat{x}$, and GQM is solved:

(11) $$\begin{array}{ccc} \Delta x^{\prime } & = & \Delta v^{\prime }{\left(I-B\right)}^{-1}=\Delta v^{\prime }{\left(I-{\hat{x}}^{-1}A\hat{x}\right)}^{-1}\\ & = & \Delta v^{\prime }{\left({\hat{x}}^{-1}\hat{x}-{\hat{x}}^{-1}A\hat{x}\right)}^{-1}=\Delta v^{\prime }{\hat{x}}^{-1}{\left(I-A\right)}^{-1}\hat{x}. \end{array}$$

Here, suppose that the relative price of primary input changes only in the *l*th factor (e.g., labor) and does not change in other factors. Let $\Delta v_{l}^{p^{{}^{\prime }}}$ be the relative change in value added in the *l*th factor:

(12) $$\Delta v_{l}^{p^{\prime }}=\Delta v_{l}^{\prime }{\hat{x}}^{-1},$$

where $\Delta v_{l}^{\prime }$ is the corresponding value added. Substituting $\Delta v_{l}^{p^{\prime }}$ and multiplying ${\hat{x}}^{-1}$ in Equation (11):

(13) $$\Delta x^{\prime }{\hat{x}}^{-1}=\Delta v_{l}^{p^{\prime }}{\left(I-A\right)}^{-1}=\Delta v_{l}^{p^{\prime }}L.$$

Let $\Delta p^{\prime }$ and $\Delta (p_{v}^{\prime }C)$ be the change ratios in production and primary input, respectively. Note that $\Delta (p_{v}^{{}^{\prime }}C)$ does not always mean C is fixed:

(14) $$\Delta \left(p_{v}^{\prime }C\right)=\left(\Delta p_{v}^{\prime }\right)\left(\Delta C\right)\neq \Delta p_{v}^{\prime }C.$$

$\Delta p^{\prime }$ is expressed:

(15) $$\Delta p^{\prime }=\Delta x^{\prime }{\hat{x}}^{-1}=\Delta v_{l}^{p^{\prime }}L=\Delta \left(p_{v}^{\prime }C\right)L.$$

Thus, it is exactly the same as LPM, given that quantity is fixed. In other words, GQM can be interpreted as a price model.

Finally, GPM (“the demand‐pull price model”; Davar, 1989; Oosterhaven, 1989) is expressed as:

(16) $$p=\mathrm{Bp}+Dp_{y}.$$

**D** is the *I × M*‐matrix of the fixed final output coefficients:

(17) $$Y=\hat{x}D\Leftrightarrow D={\hat{x}}^{-1}Y.$$

**p** is an *I*‐vector of index price [*p_i_*] for sectoral input (unit revenue for suppliers), and **p_y_** is an *M*‐vector [*p_ym_*] of index price for final outputs per category. **p** and **p_y_** are usually a unit vector (however, the lengths I and M are different). The sum of weights regarding **B** and **D** is equal to 1 (Bi+Di=i). GPM is solved:

(18) $$p={\left(I-B\right)}^{-1}Dp_{y}=\mathrm{GD}p_{y}.$$

GPM is mathematically equivalent to LQM, given that the price is fixed and the quantity is variable (Dietzenbacher, 1997). Following Park (2007), recall that $A=\hat{x}B{\hat{x}}^{-1}$, and LQM is solved:

(19) $$\begin{array}{ccc} \Delta x & = & {\left(I-A\right)}^{-1}\Delta y={\left(\hat{x}{\hat{x}}^{-1}-\hat{x}B{\hat{x}}^{-1}\right)}^{-1}\Delta y\\ & = & \hat{x}{\left(I-B\right)}^{-1}{\hat{x}}^{-1}\Delta y. \end{array}$$

By multiplying ${\hat{x}}^{-1}$ in Equation (19), the change ratio in production $\left({\hat{x}}^{-1}\Delta x\right)$ is expressed as:

(20) $$\begin{array}{ccc} {\hat{x}}^{-1}\Delta x & = & {\hat{x}}^{-1}\hat{x}{\left(I-B\right)}^{-1}{\hat{x}}^{-1}\Delta y={\left(I-B\right)}^{-1}{\hat{x}}^{-1}\Delta y\\ & = & G{\hat{x}}^{-1}\Delta y. \end{array}$$

Let Δp and $\Delta (Dp_{y})$ be the change ratios in total production and final demand, respectively. Note that $\Delta (Dp_{y})$ does not always mean D is fixed:

(21) $$\Delta \left(Dp_{y}\right)=\left(\Delta D\right)\left(\Delta p_{y}\right)\neq D\Delta p_{y}.$$

Δp is expressed as:

(22) $$\Delta p={\hat{x}}^{-1}\Delta x=G{\hat{x}}^{-1}\Delta y=G\Delta \left(Dp_{y}\right).$$

Thus, it is exactly the same as GPM, given that the price is fixed. In other words, GPM can be interpreted as a quantity model.

### 2.2 Two Implicit Assumptions

Park (2007) argues that IOA has two implicit assumptions: the newly required value added and final demand (Ghosh, 1958). Regarding the former, the ratio of the newly required value added over total input is defined as **c**:

(23) $$c=i^{\prime }C=i^{\prime }V{\hat{x}}^{-1}=v^{\prime }{\hat{x}}^{-1}.$$

cis an *I*‐vector and indeed the absolute price of primary inputs:

(24) $$\begin{array}{ccc} & & c=p_{v}^{\prime }C=i^{\prime }V{\hat{x}}^{-1}=v^{\prime }{\hat{x}}^{-1}\Leftrightarrow \Delta c=\Delta \left(p_{v}^{\prime }C\right)\\ & & \;=\Delta \left(i^{\prime }V{\hat{x}}^{-1}\right)=\Delta \left(v^{\prime }{\hat{x}}^{-1}\right). \end{array}$$

$\Delta (v^{\prime }{\hat{x}}^{-1})$ means either or both **x** and **v** change. The newly required value added is expressed as:

(25) $$\Delta v^{\prime }=\Delta v^{\prime }{\hat{x}}^{-1}\hat{x}=\left(\Delta c\right)\hat{x}.$$

Here, we assume that $\left(\Delta c\right)\hat{x}$ is equal to $c\hat{\Delta x}$, meaning that a certain change in **c** is passed only onto the change in production:

(26) $$\left(\Delta c\right)\hat{x}=c\hat{\Delta x}=\Delta x^{\prime }\hat{c}.$$

Equation (26) can be solved because of multiplying $x^{\prime }$ and c (both *I*‐vectors) in an element‐wise way. By multiplying ${\hat{x}}^{-1}$ in Equation (26):

(27) $$\begin{array}{ccc} \Delta \left(p_{v}^{\prime }C\right) & = & \Delta c=\left(\Delta c\right)\hat{x}{\hat{x}}^{-1}=c\hat{\Delta x}{\hat{x}}^{-1}=\Delta x^{\prime }{\hat{x}}^{-1}\hat{c}\\ & = & \Delta p^{{}^{\prime }}\hat{c}. \end{array}$$

Notice that $\Delta x^{\prime }{\hat{x}}^{-1}$ is $\Delta p^{\prime }$, given that quantity is fixed (because of LPM). Thus, this implies that a certain change in the primary input price $\left(\Delta (p{}_{v}^{\prime }C)\right)$ should be transferred from the change in the total input price $\left(\Delta p^{\prime }\right)$ via $\hat{c}$.

Similarly, regarding the latter, the ratio of the final demand (y) over total output (x) is defined as **d**:

(28) $$d=\mathrm{Di}={\hat{x}}^{-1}\mathrm{Yi}={\hat{x}}^{-1}y.$$

**d** is an *I*‐vector, and indeed, the change ratio of final demand, given that price is fixed (because of GPM):

(29) $$d=Dp_{y}={\hat{x}}^{-1}y\Leftrightarrow \Delta d=\Delta \left(Dp_{y}\right)=\Delta \left({\hat{x}}^{-1}y\right).$$

$\Delta ({\hat{x}}^{-1}y)$ means either or both of **x** and **y** change. The newly required final demand is expressed as:

(30) $$\Delta y=\hat{x}{\hat{x}}^{-1}\Delta y=\hat{x}\left(\Delta d\right).$$

Here, we assume that $\hat{x}\left(\Delta d\right)$ is equal to $\hat{\Delta x}d$, meaning that a certain change in **d** is passed only onto the change in production:

(31) $$\hat{x}\left(\Delta d\right)=\hat{\Delta x}d=\hat{d}\Delta x.$$

Equation (31) can be solved because of multiplying x and d (both *I*‐vectors) in an element‐wise way. By multiplying ${\hat{x}}^{-1}$ in Equation (31):

(32) $$\begin{array}{ccc} \Delta \left(Dp_{y}\right) & = & \Delta d={\hat{x}}^{-1}\hat{x}\left(\Delta d\right)={\hat{x}}^{-1}\hat{\Delta x}d=\hat{d}{\hat{x}}^{-1}\Delta x\\ & = & \hat{d}\Delta p. \end{array}$$

Notice that ${\hat{x}}^{-1}\Delta x$ is Δp, given that price is fixed (because of GPM). This implies that a certain change ratio of final demand $\left(\Delta (Dp_{y})\right)$ should be transferred from the change ratio of production (Δp) via $\hat{d}$.

### 2.3 Implausibility of the IO Models

In the IO literature, Oosterhaven (1988) has argued that GQM is implausible (see Supplementary Information A). In summary, GQM converts (delivers) the additional primary input (Δ**v**) into final demand (**y**) in a perfectly elastic way (without any technological relationship), meaning that “consumers will buy whatever is supplied to them” (Oosterhaven, 2012, p.106). Implausibility implies that intermediate demand (input ratios) varies arbitrarily and that a production function is not necessary. Adding to the debate on implausibility (Gruver, 1989; Rose & Allison, 1989), Dietzenbacher (1997) argued that the four IO models (LQM, GQM, LPM, and GPM) should be divided into either the demand‐pull (LQM and GPM, given the fixed price) or cost‐push models (LPM and GQM, given the fixed quantity) (Section 2.1; Fig. 1).

## 3 MODELING OF SUPPLY CONSTRAINT

### 3.1 Introduction of This Section

This section proposes the supply‐driven IO model with reference to Park (2007; the unpublished paper). As in Section 2, a basic IOA can analyze only the following four changes: final demand (Δy) in LQM (Equation (3)) and price for final outputs $\left(\Delta p_{y}\right)$ in GPM (Equation (18)) as the demand‐pull models; and value added $\left(\Delta v^{\prime }\right)$ in GQM (Equation (10)) and price for value added $\left(\Delta p_{v}^{{}^{\prime }}\right)$ in LPM (Equation (6)) as the cost‐push models.

As an issue, the supply constraint is usually a quantity issue on the supply side, but GQM itself is considered implausible (Sections 2.3 and 3.2). Instead of GQM, Park (2007) proposes to use GPM (Equations (22)–(32)) for the supply constraint by introducing the price elasticity of demand (Section 3.3). Note that Park (2009; the unpublished paper) applied this approach to conduct an economic analysis of the U.S. oil industry based on changes in crude oil prices (see Supplementary Information B).

This study modifies Park (2007) in the following two ways. First, because GPM is a demand‐side model, this study supposes that LPM is appropriate for analyzing the supply constraints (Section 3.5). Second, because production itself is less informative as a damage index than profit, this study proposes to use the loss of SS as the damage index (Sections 3.4 and 3.6; for comparison between IOA, this study, and CGE, see Supplementary Information C). Section 3.7 (Supplementary Information D) discusses how this study relates to the methods proposed in the previous studies regarding the seven issues.

### 3.2 The Difficulty of Supply Constraint in IOA

An exogenous shock such as a disaster often disrupts supply (i.e., supply constraint). Although we often know how much production decreases due to a shock via sectoral production statistics (typically at the monthly level), the supply constraint is difficult to consider in IOA. First, the supply constraint is usually a quantity issue. Even if price rises, quantity decreases; however, the fact that consumers will reduce the purchase quantity because of the price is just a reflection of a supply‐demand balance. Therefore, IO price models are usually inadequate because they assume that quantity is fixed (however, Dietzenbacher, 1997, interpreted GPM as a quantity model; Section 2.1).

LQM cannot usually handle the supply constraint because LQM assumes that total output is endogenous. In the literature, the inoperability IO model (e.g., Haimes & Jiang, 2001; Santos, 2006) considers the supply constraint; however, this model is regarded as suspicious for usability because it is based on LQM (Oosterhaven, 2017).

Note that many disaster studies have used LQM (e.g., Crawford‐Brown et al., 2013; Hallegatte, 2008; Li et al., 2013; Okuyama, 2007; Steenge & Bočkarjova, 2007). A basic application is to adopt the survival coefficient (or production capacity) $\Theta =[\theta_{i}]$ for total output (or input) (Δ**x**) in sector *i*. $\theta_{i}$ is unity before a disaster and $0\leq \theta_{i}\leq 1$ after a disaster.

(33) $$\hat{\Theta }x=x+\Delta x.$$

Again, however, the surviving total output $\left(\hat{\Theta }x\right)$ is basically endogenous.

Finally, GQM is applied in some disaster studies (e.g., for a recent survey, see Galbusera & Giannopoulos, 2018; Shimoda & Fujikawa, 2012). GQM is numerically capable if we know an exogenous change in primary input (Δ**v**) due to a supply constraint in Equation (10). However, GQM is not popular because of its implausibility (Oosterhaven, 1988, 2017).

### 3.3 Supply Constraint in Park (2007)

**Fig 2:**
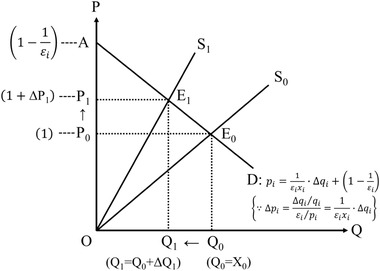
Demand and supply model with the supply constraint.

Park (2007) has uniquely considered the supply constraint in GPM. Note that Park (2009) adopts a similar method (see Supplementary Information B). Park (2007) first proposes that the supply constraint may decrease the quantity and increase the price as in basic economics. In Fig. 2, S and D are basic supply and demand curves; E is an equilibrium; and subscripts 0 and 1 are before and after the constraint, respectively. Suppose that the demand curve is fixed; if the quantity is constrained from Q_0_ to Q_1_, the supply curve moves from S_0_ to S_1_, depending on the fixed demand curve. Thus, the market price increases from P_0_ to P_1_, and production will change from Q_0_ times P_0_ to Q_1_ times P_1_.

**Fig 3:**
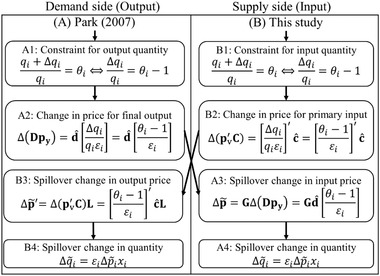
Difference between Park (2007) and this study.

Park (2007) proposes using GPM for the supply constraint in the following four steps (A1 to A4; Fig. 3). (A1) Output quantity is exogenously constrained in certain sectors. (A2) Using A1 and the exogenous price elasticity of demand (ε), the price of final outputs (Δp_y_) changes (increases). (A3) The spillover effect of Δp_y_ changes (increases) the input price ($\Delta \tilde{p}$) (where tilde means the estimation or spillover effect in this study). (A4) Based on A3, input quantity is estimated.

Specifically, (A1) the price elasticity of demand (ε) in sector *i* is exogenously defined as:

(34) $$\varepsilon_{i}=\frac{\Delta q_{i}/q_{i}}{\Delta p_{i}/p_{i}}.$$

ε is the arc elasticity here (not the point elasticity). Let the base price (*p_i_*) be one and the base quantity (*q_i_*) be equal to *x_i_* (= *p_i_q_i_*). The change of price is expressed as:

(35) $$\Delta p_{i}=\frac{\Delta q_{i}/q_{i}}{\varepsilon_{i}/p_{i}}=\frac{\Delta q_{i}}{q_{i}\varepsilon_{i}}.$$

We may use the survival coefficient $\theta_{i}$ instead of the quantity ratio:

(36) $$\frac{q_{i}+\Delta q_{i}}{q_{i}}=\theta_{i}\Leftrightarrow \frac{\Delta q_{i}}{q_{i}}=\theta_{i}-1.$$

(A2) The newly required price of final output ($\Delta (Dp_{y})$) is calculated (Equation (32)) as follows:

(37) $$\Delta \left(Dp_{y}\right)=\hat{d}\Delta p=\hat{d}\left[\frac{\Delta q_{i}}{q_{i}\varepsilon_{i}}\right]=\hat{d}\left[\frac{\theta_{i}-1}{\varepsilon_{i}}\right].$$

(A3) The spillover change in price $\left(\Delta \tilde{p}\right)$ is calculated (Equation (22)):

(38) $$\Delta \tilde{p}=G\Delta \left(Dp_{y}\right)=G\hat{d}\left[\frac{\Delta q_{i}}{q_{i}\varepsilon_{i}}\right]=G\hat{d}\left[\frac{\theta_{i}-1}{\varepsilon_{i}}\right].$$

(A4) Based on A3 and ε, the spillover change in input quantity $\left(\Delta {\tilde{q}}_{i}\right)$ is estimated. Because Park (2007) does not specify this procedure, Subsection 3.4 discusses it.

### 3.4 Generalization of Elasticity Between Price and Quantity

A feature of Park (2007) is the introduction of the price elasticity of demand into IOA. As Oosterhaven (1996) pointed out, IOA usually assumes that price (*p*) and quantity (*q*) of production (*x*) are independent of each other. This study presupposes that changes in production are not adequate to use as a damage index because, unlike profit, production does not tell exactly how much damage to the supply constraint is passed along to each supplier (upstream sectors) and each buyer (downstream sectors). For example, suppose that the supply quantity is constrained to 80%, increasing the price to 125%. Thus, production is 100%, and the suppliers may not be damaged; however, the buyers will be negatively affected because they will buy smaller quantities at the higher price.

In IOA, even if price changes exogenously by $\Delta p_{i}$ in sector *i*, because quantity does not change $\left(s\Delta q_{i}=0\right)$, production increases by $\left(\Delta p_{i}x_{i}\right)$:

(39) $$\left(p_{i}+\Delta p_{i}\right)\left(q_{i}+\Delta q_{i}\right)=\left(1+\Delta p_{i}\right)x_{i}=x_{i}+\Delta p_{i}x_{i},$$

where $\left(p_{i}=1\right)$ and $\left(q_{i}=x_{i}\right)$.

In Park (2007), quantity is elastic with respect to price:

(40) $$\varepsilon_{i}=\frac{\Delta q_{i}/q_{i}}{\Delta p_{i}/p_{i}}=\frac{\Delta q_{i}}{\Delta p_{i}x_{i}}\Rightarrow \Delta q_{i}=\varepsilon_{i}\Delta p_{i}x_{i}.$$

If price changes exogenously by $\Delta p_{i}$, production will increase by $\left(1+\varepsilon_{i}+\varepsilon_{i}\Delta p_{i}\right)\Delta p_{i}x_{i}$:

(41) $$\begin{array}{ccc} & & \left(p_{i}+\Delta p_{i}\right)\left(q_{i}+\Delta q_{i}\right)=\left(1+\Delta p_{i}\right)\left(x_{i}+\varepsilon_{i}\Delta p_{i}x_{i}\right)\\ & & \;=x_{i}+\left(1+\varepsilon_{i}+\varepsilon_{i}\Delta p_{i}\right)\Delta p_{i}x_{i}. \end{array}$$

Notice that Equation (39) is a special form of Equation (41), assuming that $\left(1+\varepsilon_{i}+\varepsilon_{i}\Delta p_{i}\right)$ is one.

(42) $$\left(1+\varepsilon_{i}+\varepsilon_{i}\Delta p_{i}\right)=1\Rightarrow \varepsilon_{i}\left(1+\Delta p_{i}\right)=0.$$

Thus, IOA usually assumes that $\varepsilon_{i}$ is 0. $\Delta p_{i}$ is not usually –1 because if so, a price $\left(p_{i}+\Delta p_{i}\right)$ becomes 0.

Note that the supply constraint usually increases price $\left(\Delta p_{i}>0\right)$. Therefore, production (Equation (41)) will decrease when $\left(1+\varepsilon_{i}+\varepsilon_{i}\Delta p_{i}\right)$ is negative:

(43) $$\begin{array}{c} 1+\varepsilon_{i}+\varepsilon_{i}\Delta p_{i}=1+\varepsilon_{i}\left(1+\Delta p_{i}\right)<0\Leftrightarrow \varepsilon_{i}<-\frac{1}{1+\Delta p_{i}}. \end{array}$$

Equation (43) holds when $\varepsilon_{i}$ is elastic to some degree (at least less than –1 when $\Delta p_{i}=0$). Otherwise, production will even increase after the supply constraint.

### 3.5 Extension to the Cost‐Push Price Model

This and the next subsections aim to address two issues in Park (2007). This study argues that GPM in Park (2007) is inadequate for making economic interpretations (of the supply constraint), which is consistent with the assumptions in the following two points (see Subsection 2.3). First, GPM is a demand‐pull model (Dietzenbacher, 1997), and hence, it is a demand‐side analysis (i.e., the demand constraint). Second, if we are following Dietzenbacher (1997), GPM is a “quantity” model (Equations (19)–(22)), and therefore, GPM cannot handle price change. Thus, this study supposes that LPM is adequate for the supply constraint. First, LPM is the cost‐push model and is for the supply‐side analysis. Second, LPM can handle price change.

Specifically, this study proposes to use LPM in the following four steps (B1–B4; Fig. 3). (B1) Input quantity is exogenously constrained in certain sectors. (B2) Using B1 and the exogenous price elasticity of demand, the price of primary inputs $\left(\Delta p_{v}\right)$ changes (increases) from Equation (27):

(44) $$\Delta \left(p_{v}^{\prime }C\right)=\Delta p^{\prime }\hat{c}={\left[\frac{\Delta q_{i}}{q_{i}\varepsilon_{i}}\right]}^{\prime }\hat{c}={\left[\frac{\theta_{i}-1}{\varepsilon_{i}}\right]}^{\prime }\hat{c}.$$

(B3) The spillover effect of Δ**p_v_** changes (increases) output price ($\Delta \tilde{p}$) (Equation (15)).

(45) $$\begin{array}{ccc} & & \Delta {\tilde{p}}^{\prime }=\Delta \left(p_{v}^{{}^{\prime }}C\right)L=\Delta p^{\prime }\hat{c}L={\left[\frac{\Delta q_{i}}{q_{i}\varepsilon_{i}}\right]}^{{}^{\prime }}\hat{c}L\\ & & \;={\left[\frac{\theta_{i}-1}{\varepsilon_{i}}\right]}^{{}^{\prime }}\hat{c}L. \end{array}$$

(B4) Based on B3 and ε, spillover change in output quantity $\left(\Delta \tilde{q}\right)$ is estimated, such as from Equation (40):

(46) $$\Delta {\tilde{q}}_{i}=\varepsilon_{i}\Delta {\tilde{p}}_{i}x_{i}.$$

Note that the elasticities (ε) are set as the same for Equations (44) and (46) for simplification, but they can be different: such as $\varepsilon^{s}$ (for supply “s”) in Equation (44) and $\varepsilon^{d}$ (for demand “d”) in Equation (46).

### 3.6 Social Surplus Loss as a Damage of Supply Constraint

The other issue is that Park (2007) estimates production as damage, which is obtained by multiplying total inputs and price changes in the next period (*x* times Δ*p*; see Supplementary Information B). Unlike profit, however, changes in production do not exactly tell us how much damage is passed on to each supplier (upstream sectors) and each buyer (downstream sectors). Even if production increases after the supply constraint (as in Equations (39)–(43)), consumers, in particular, may be damaged because the constraint decreases quantity and increases price.

Instead, this study proposes to use the loss of SS as the damage because it can identify the damage incurred by each buyer and seller. Note that the estimation of SS is common practice in CGE applications (e.g., Koks et al., 2016), who conduct both IOA and CGE for flood analysis). CGE looks ideal for disaster analysis because it is theoretically consistent; however, CGE is usually much harder to estimate than IOA (see Supplementary Information C).

For simplicity, as in Fig. 2, this study supposes that the demand (*D*) and supply (*S*) curves are linear, and that *D* does not change, whereas *S* changes from *S*_0_ to *S*_1_ (note that *S*_0_ and *S*_1_ are supposed to go through the origin O). Specifically, the demand curve (D) in sector *i* has a slope of $\frac{1}{\varepsilon_{i}x_{i}}$ from Equations (34) and (35), and hence, has an intercept $\left(1-\frac{1}{\varepsilon_{i}}\right)$.

Suppose that before the disaster, quantity is production ($q_{i}=x_{i}$) and price is one $\left(p_{i}=1\right)$ in sector *i*. SS ($ss_{i}^{before}$) before the supply constraint (in sector *i*) is ΔAOE_0_, where CS ($cs_{i}^{before}$) is ΔAP_0_E_0_, and PS ($ps_{i}^{before}$) is ΔOP_0_E_0_ (notice that PS is half of $x_{i}$).

(47) $$\begin{array}{ccc} & & ss_{i}^{before}=cs_{i}^{before}+ps_{i}^{before}=\Delta \mathrm{AO}E_{0}\\ & & \;=\frac{1}{2}\cdot \mathrm{AO}\Delta P_{0}E_{0}=\frac{1}{2}\cdot \left(1-\frac{1}{\varepsilon_{i}}\right)\cdot x_{i}\\ & & \;=\left(\frac{1-\varepsilon_{i}}{2}\right)x_{i}=-\frac{x_{i}}{2\varepsilon_{i}}+\frac{x_{i}}{2}, \end{array}$$

(48) $$\begin{array}{ccc} cs_{i}^{before} & = & \Delta AP_{0}E_{0}=\frac{1}{2}\cdot AP_{0}\cdot P_{0}E_{0}=\frac{1}{2}\cdot \left(-\frac{1}{\varepsilon_{i}}\right)\cdot x_{i}\\ & = & -\frac{x_{i}}{2\varepsilon_{i}}, \end{array}$$

(49) $$\begin{array}{ccc} ps_{i}^{before} & = & \Delta OP_{0}E_{0}=\frac{1}{2}\cdot OP_{0}\cdot P_{0}E_{0}=\frac{1}{2}\cdot 1\cdot x_{i}\\ & = & \frac{x_{i}}{2}. \end{array}$$

Meanwhile, suppose that after the disaster in sector *i*, the price changes from one to $\left(1+\Delta {\tilde{p}}_{i}\right)$ from Equation (45), whereas quantity changes from $x_{i}$ to $\left(1+\varepsilon_{i}\Delta {\tilde{p}}_{i}\right)x_{i}$ from Equation (46). Thus, new production $\left(x_{i}^{after}\right)$ is calculated:

(50) $$x_{i}^{after}=\left(1+\Delta {\tilde{p}}_{i}\right)\cdot \left(1+\varepsilon_{i}\Delta {\tilde{p}}_{i}\right)x_{i}.$$

SS after the supply constraint ($ss_{i}^{after}$) is ΔAOE_1_, where CS ($cs_{i}^{after}$) is ΔAP_1_E_1_, and PS ($ps_{i}^{after}$) is ΔOP_1_E_1_.

(51) $$\begin{array}{ccc} ss_{i}^{after} & = & cs_{i}^{after}+ps_{i}^{after}=\Delta \mathrm{AO}E_{1}\\ & = & \frac{1}{2}\cdot \mathrm{AO}\cdot P_{1}E_{1}=\frac{1}{2}\cdot \left(1-\frac{1}{\varepsilon_{i}}\right)\cdot \left(1+\varepsilon_{i}\Delta {\tilde{p}}_{i}\right)x_{i}\\ & = & \left(1+\varepsilon_{i}\Delta {\tilde{p}}_{i}\right)\cdot ss_{i}^{before}, \end{array}$$

(52) $$\begin{array}{ccc} cs_{i}^{after} & = & \Delta AP_{1}E_{1}=\frac{1}{2}\cdot AP_{1}\cdot P_{1}E_{1}\\ & = & \frac{1}{2}\cdot \left[\left(1-\frac{1}{\varepsilon_{i}}\right)-\left(1+\Delta {\tilde{p}}_{i}\right)\right]\cdot \left(1+\varepsilon_{i}\Delta {\tilde{p}}_{i}\right)x_{i}\\ & = & \frac{1}{2}\cdot \left(1-\frac{1}{\varepsilon_{i}}\right)\cdot \left(1+\varepsilon_{i}\Delta {\tilde{p}}_{i}\right)x_{i}\\ & & \;-\frac{1}{2}\cdot \left(1+\Delta {\tilde{p}}_{i}\right)\cdot \left(1+\varepsilon_{i}\Delta {\tilde{p}}_{i}\right)x_{i}=ss_{i}^{after}\\ & & \;-\frac{x_{i}^{after}}{2}, \end{array}$$

(53) $$\begin{array}{ccc} ps_{i}^{after} & = & \Delta OP_{1}E_{1}=\frac{1}{2}\cdot OP_{1}\cdot P_{1}E_{1}\\ & = & \frac{1}{2}\cdot \left(1+\Delta {\tilde{p}}_{i}\right)\cdot \left(1+\varepsilon_{i}\Delta {\tilde{p}}_{i}\right)x_{i}=\frac{x_{i}^{after}}{2}. \end{array}$$

Therefore, SS (ΔAOE_0_ and ΔAOE_1_) are two triangles, and they have the same base (AO) and different height, P_0_E_0_ (= Q_0_) and P_1_E_1_ (= Q_1_), depending only on the change in height (quantity).

Specifically, this study proposes changes in SS, CS, and PS as damage indicators for the whole industry, the downstream sectors, and the upstream sectors, respectively. In sector *i*, the loss of SS ($\Delta ss_{i}$) is $ss_{i}^{before}$ minus $ss_{i}^{after}$:

(54) $$\begin{array}{ccc} \Delta ss_{i} & = & ss_{i}^{before}-ss_{i}^{after}=ss_{i}^{before}-\left(1+\varepsilon_{i}\Delta {\tilde{p}}_{i}\right)\cdot ss_{i}^{before}\\ & = & -\varepsilon_{i}\Delta {\tilde{p}}_{i}\cdot ss_{i}^{before}. \end{array}$$

The loss of CS ($\Delta cs_{i}$) is $cs_{i}^{before}$ minus $cs_{i}^{after}$, which is a trapezoid P_1_P_0_E_0_E_1_:

(55) $$\begin{array}{ccc} \Delta cs_{i} & = & cs_{i}^{before}-cs_{i}^{after}=\frac{1}{2}\cdot \left(P_{1}E_{1}+P_{0}E_{0}\right)\cdot P_{1}P_{0}\\ & = & \frac{1}{2}\cdot \left(\left(1+\varepsilon_{i}\Delta {\tilde{p}}_{i}\right)x_{i}+x_{i}\right)\cdot \Delta {\tilde{p}}_{i}\\ & = & \frac{\Delta {\tilde{p}}_{i}\left(2+\varepsilon_{i}\Delta {\tilde{p}}_{i}\right)}{2}\cdot x_{i}. \end{array}$$

The loss of PS $\left(\Delta ps_{i}\right)$ is $ps_{i}^{before}$ minus $ps_{i}^{after}$:

(56) $$\begin{array}{ccc} \Delta ps_{i} & = & ps_{i}^{before}-ps_{i}^{after}=\frac{x_{i}^{after}}{2}-\frac{x_{i}}{2}\\ & = & -\frac{\Delta {\tilde{p}}_{i}\left(1+\varepsilon_{i}+\varepsilon_{i}\Delta {\tilde{p}}_{i}\right)}{2}\cdot x_{i}. \end{array}$$

### 3.7 Applications to the Approaches in the Previous Studies

Some readers may wonder how this study relates to the methods proposed in the previous studies. Due to space limitations, Supplementary Information D briefly discusses the following seven items: the endogenous recovery for the survival coefficient (D.2), the sequential interindustry models in Romanoff (1984) and Okuyama, Hewings, and Sonis (2004) (D.3), the impact on transportation networks in Sohn, Hewings, Kim, Lee, and Jang (2004) and Kim, Ham, and Boyce (2002) (D.4), the input–occupancy–output model in Chen (1990) and Chen, Guo, and Yang (2005) (D.5), the extension to the CGE model in Kratena et al. (2013), Kratena, Streicher, Salotti, Sommer, and Jaramillo (2017), and Kratena and Streicher (2017) (D.6), spatial substitution and price multipliers (D.7), and the supply constraint in GQM (D.8).

## 4 APPLICATION TO EARTHQUAKES IN JAPAN

### 4.1 Multiregional IO Table in Japan

As a model application, this study examines the effect of supply constraint in the largest earthquakes in Japan (Table I). This study uses the 2005 MRIO table at the prefecture level (Hasegawa, Kagawa, & Tsukui, 2015), which covers the 47 prefectures of Japan. This table includes 80 industry sector classifications (#1 to #80), where 54 sectors from #2 to #55 are mining and manufacturing sectors (industrial sectors), and 26 sectors (#1, #56 to #80) are agricultural and service sectors (nonindustrial sectors) (see Supplementary Information Tables S4–S9). Thus, there are 3,760 industry‐prefectures (80 sectors in 47 prefectures). Because of the data restriction for production capacity, this study examines the 54 mining and industrial sectors out of 80 sectors.

An important feature of earthquakes is that the resultant damage does not tend to spill over to other prefectures. An earthquake with a large magnitude (e.g., M8.0 or more) will shake greatly in the prefecture closest to the epicenter (e.g., “Shindo” [seismic intensity scale in Japan] is “lower‐five” or above), increasing the risk of human injuries and buildings collapsing. Meanwhile, it will not shake as much in other prefectures far from the epicenter (e.g., Shindo is four or below).

As an exception, however, the GEJE damaged a vast area across prefectures (Hayes et al., 2017). The GEJE not only caused damage as a result of the earthquake, but also due to the tsunami (washing away coastal housing, other buildings, and infrastructure; power problems associated with the Fukushima nuclear power plant accident; and medical problems). This explains why the GEJE is referred to as a triple disaster (Managi & Guan, 2017). The triple disaster also affected undamaged prefectures indirectly in terms of power outages, disruption to logistics and supply chains, and so on. Thus, the prefectural MRIO table is suitable for analyzing huge disasters such as the GEJE because it can consider damage incurred across different prefectures.

The reason for using the 2005 version of the MRIO table (Hasegawa et al., 2015) is that the growth rate of nominal gross domestic product (GDP) is meager between 1995 and 2016 (approximately 0.21% on average): 516 trillion (T), 525T, and 539T Japanese yen (JPY) in 1995, 2005, and 2016, respectively (Cabinet Office, Government of Japan, 2018) (i.e., the so‐called lost two decades). Because the industrial structure may change nontrivially over time, however, it is assumed herein that the industrial structure remained as per 2005 during all periods.

The original MRIO table contains annual values, but this study uses monthly values, simply dividing the annual value by 12 (months). If necessary, the production value can be seasonally adjusted. As in GDP statistics, a popular method is to create “centered ratios” for every month by using the (past) 12‐month centered moving average. Note that if the input coefficient varies every month, it will require the IO table of each month.

Production (**x**) at the monthly level is 80,793 billion (B) JPY (100%) for all sectors, 25,263B JPY (31%) for the 54 mining and industrial sectors, and 55,530B JPY (69%) for the other 26 sectors. Similarly, final demand (**y**) at the monthly level is 46,870B JPY (100%) for all sectors, 11,455B JPY (24%) for the 54 mining and industrial sectors, and 35,415B JPY (76%) for the others.

### 4.2 Research Settings

As research settings (Table I), the focal prefectures are Hyogo for H95Jan, Niigata for N04Oct and N07Jul, Iwate and Miyagi for IM08Jun, and Kumamoto for K16Apr. Those for GEJE are Fukushima, Iwate, Miyagi, Ibaraki, and Chiba (FIMIC prefectures), which experienced the triple disaster, and non‐FIMIC prefectures (i.e., 42 of the 47 prefectures), which experienced power outages and disruption to logistics and supply chains. As the selection criteria, the prefecture that was closest to the epicenter is focused. However, in the case of IM08Jun, because the epicenter is near the prefectural border between Iwate and Miyagi, we have chosen two prefectures exceptionally.

Regarding the initial and occurrence terms, the initial month before each earthquake (*t* = 0) is set to May 1995 for H95Jan, September 2004 for N04Oct, June 2007 for N07Jul, May 2008 for IM08Jun, March 2016 for K16Apr, and February 2011 for GEJE. Similarly, the month in which each earthquake occurred (*t* = 1) is set to the next months, except for January 1995 in H95Jan. Regarding H95Jan, the month before the disaster (*t* = 0) is set as May 1995 because data were unavailable for December 2014, and production capacity in 1995 was at its highest in May. The analysis periods are 12 months for H95Jan, N04Oct, N07Jul, IM08Jun, and K16Apr, and 48 months for GEJE.

### 4.3 Data: Production Capacity

Regarding production capacity (*θ*), this study uses seasonally adjusted indices of industrial production (IIP) published by the statistics office of each prefecture (at different points in time) such as the Hyogo prefecture (2017) for H95Jan, and the Statistics division of the Niigata prefecture (2018) for N04Oct and N07Jul. Note that the Ministry of Economy, Trade, and Industry, Japan (2018) summarizes each prefectural IIP since January 2008.

IIP covers production (in all prefectures), shipments, and inventories, and this study uses IIP because it has abundant production data as an actual index. Because of the real index, however, IIP has a drawback in that it is affected not only by the direct effect of disaster, but also by the indirect effect among sectors, which may be somewhat mitigated by the inventories (see Supplementary Information E).

Because IIP only covers the mining and industrial sectors, analysis is restricted herein to the 54 mining and industrial sectors (#2 to #55) out of 80 sectors in the MRIO table (see Supplementary Information Tables S4–S9). As an issue, however, the IIP sector classification differs from the MRIO table classification (80 sectors). This study uses the common 26‐sector classification, connecting the industrial classification of the MRIO table (from #1 to #80) and the IIP id (from #1 to #26). Because there are missing values (i.e., missing sectors) in certain prefectures, this study substitutes another similar IIP id for missing values (see Supplementary Information Tables S4–S5).

The production capacity at *t* for sector *i* ($\theta_{i}\left(t\right)$) is configured:

(57) $$\theta_{i}\left(t\right)=\left\{\begin{array}{cc} \mathrm{II}P_{i}\left(t\right)/\mathrm{II}P_{i}\left(0\right) & \;\mathrm{if}\;i\in K\\ 1 & \;\mathrm{if}\;i\notin K \end{array}\right.,$$

where $\mathrm{II}P_{i}\left(t\right)$ denotes the original IIP in sector *i* at month *t*. *K* means the set of damaged areas/sectors, and $\theta_{i}\left(t\right)$ is 1 when i∉K. Supplementary Information for the raw data set includes $\theta_{i}\left(t\right)$ (i∈K) at IIP id, with missing values for each disaster (Excel sheets: H95Jan, N04Oct, N07Jul, IM08Jun, K16Apr, and GEJE) and information on IO id (“ioid”), IIP id (“iipid”), and area id (48 prefectures in “areaid”).

### 4.4 Data: Price Elasticity of Demand

To estimate price elasticity (ε), this study uses the following regression model in log form:

(58) $$\ln q_{it}=\sum_{i}D_{i}\ln\alpha_{i}+\sum_{i}D_{i}\varepsilon_{i}\ln p_{it}+e_{it},$$

where q is quantity and p is price in sector i in year *t*. *D_i_* is a dummy variable (0 or 1) for sector *i*. ε is the coefficient of the log price, representing the price elasticity of demand. $\ln\alpha_{i}$ is a constant term in each sector *i*, and *e* is an error term. Note that this study uses industrial dummy variables to estimate the constant term ($\ln\alpha_{i}$) and price elasticity ($\varepsilon_{i}$; by using interaction terms).

Regarding the data, this study uses the Japan Industrial Productivity (JIP) Database 2018 provided by the Research Institute of Economy, Trade, and Industry, Japan (2019). The database includes gross output data at real value (in chain‐linked sectoral 2011 prices) and nominal value in 100 unique JIP sectors for 22 years (1994 to 2015), and there are 2,194 observations because nursing care (#95) has six missing values. Thus, we can calculate the sectoral deflator (price) by dividing the nominal value by the real value (where the deflator is one in 2011). Using the real gross output as quantity (*q*) and the sectoral deflator as price (*p*), this study estimated the price elasticity of demand (*ε*) in each of 100 JIP sectors (see Supplementary Information Tables S10–S11).

Note that the 100 JIP sectors are different from the 80 sectors in the MRIO table again. Thus, this study made the weighted average *ε* for 17 summarized sectors to connect with the 80 MRIO sectors (by using the real gross output as of 2005), so that all values become negative. In Table II, the price elasticity (*ε*) is –1.305 in total sectors (#1–17), –0.717 in the manufacturing and mining sectors (#1–6; the focus of this study), and –1.598 in the agriculture and service sectors (#7–17).

**Table II:** Price Elasticity of Demand (Weighted by Real Gross Output as of 2005)

	(1)	(2)	(3)	(4)
#	Elasticity (ε)	Summarized industry group (#1–17)	IIP industry group (#1–80)	JIP industry group (#1–100)
1	–0.450	Mining, food products, textile/chemical fibers, and pulp	2–8,11,12,19	5–16
2	–0.643	Chemical, pharmaceutical, petroleum, and cement products	14–18, 20–23, 26–29	17–28
3	–0.378	Iron, steel, and metal	30–37	29–34
4	–0.395	Machinery and electrical products	38–49	35–48
5	–2.123	Motor vehicles and equipment	50–53	49–51
6	–0.288	Miscellaneous manufacturing industries (printing, plastic, and so on)	9,10,13,24,25,54,55	52–59
7	–0.058	Agriculture	1	1–4
8	–0.087	Utilities and waste disposal	60–62	60–65
9	–2.093	Construction	56–59	66, 67, 89
10	–1.820	Retail and wholesale	63	68, 69
11	–0.072	Transportation and mail	67, 77	70–75, 88
12	–1.879	Communications and information services	68–72,76	78–81, 87
13	–0.813	Finance and insurance	64	82, 83
14	–1.104	Housing and real estate	65,66	76, 84, 85
15	–3.270	Medical service	75	93–95
16	–2.987	Research, education, and other services for businesses	73, 74, 78	86, 90–92
17	–0.414	Other services for individuals	79, 80	77, 96–100
1–17	–1.305	Total sectors	1–80	1–100
1–6	–0.717	Manufacturing and mining sectors	5–59	2–55
7–17	–1.598	Agriculture and service sectors	1–4.60–80	1, 56–100

*Notes*: This table shows the price elasticity of demand at the weighted average value by using the real gross output (as of 2005). Columns (1) and (2) show the summarized 17 industry groups and their elasticity (ε). Columns (3) and (4) indicate the JIP Industry group (#1–100) and IIP Industry group (#1–80), respectively, to connect with the summarized industry groups (#1–17). See Supplementary Information Tables S10 and S11 for the descriptive statistics and the regression results.

### 4.5 Previous Studies Concerning Indirect Damage Estimation: H95Jan and GEJE

In disaster studies, the loss of SS is not widespread for estimating damage. Instead, two popular damages are direct damage (e.g., damage to capital stock) and indirect damage (e.g., flow damage due to the spillover effect). This study supposes that the loss of SS is similar to indirect damage because it does not consider the damage to capital stock and so on. In other words, indirect damage referred to in the previous studies consists of damage to buyers (downstream sectors) and sellers (upstream sectors), which are similar to the losses of CS and PS, respectively. This study supposes that the reason SS is not popular is that CS is difficult to estimate (although PS is easy). CS is calculated from the difference between the reservation price (i.e., willingness‐to‐pay price) and transaction price. However, the reservation price is usually difficult to estimate.

Two previous studies that estimated damage due to H95Jan (Toyoda & Kouchi, 1997) and GEJE (Hayashi, 2012) are introduced for comparative purposes (see Supplementary Information F). Toyoda and Kouchi (1997) estimated that the indirect damage caused by H95Jan for one year was 1,203B JPY for the industrial sectors (in 10 cities and 10 towns in Hyogo). Meanwhile, regarding Hayashi (2012), the indirect damage caused by GEJE was estimated to be approximately 10T JPY in Fukushima alone and approximately 100T JPY over the decade.

## 5 RESULTS

### 5.1 Production Capacity

Table III shows the production capacity (*θ*) of 54 industrial sectors in the case of each earthquake (Supplementary Information Figs. S11–S12). For simplicity, this study considers approximately 99% or more to represent a temporary recovery. Values in parentheses denote production capacity after the first temporary recovery.

**Table III:** Production capacity (*θ*)

*t* (months)	H95Jan	N04Oct	N07Jul	IM08Jun	K16Apr	GEJE (FIMIC)	GEJE (non‐FIMIC)
0	100.0%	100.0%	100.0%	100.0%	100.0%	100.0%	100.0%
(B JPY)	(1,179B)	(400B)	(400B)	(525B)	(226B)	(3,114B)	(22,150B)
1	84.5%	95.8%	96.9%	96.7%	76.6%	67.5%	86.8%
2	87.1%	95.6%	99.0%	95.7%	72.6%	72.7%	87.2%
3	89.1%	96.9%	97.5%	(92.4%)	91.6%	86.7%	91.3%
4	94.9%	97.2%	99.2%	(92.9%)	87.9%	89.3%	96.3%
5	100.0%	97.2%	99.6%	(92.3%)	99.7%	85.8%	97.3%
6	97.5%	98.2%	(100.5%)	(84.0%)	98.9%	87.7%	98.7%
7	94.1%	99.4%	(98.8%)	(76.6%)	99.6%	85.0%	97.6%
8	95.3%	99.8%	(99.4%)	(74.5%)	(106.3%)	88.4%	100.5%
9	91.2%	(98.6%)	(98.6%)	(69.8%)	(106.8%)	89.2%	(96.9%)
10	95.7%	(100.1%)	(98.0%)	(69.6%)	(106.2%)	87.1%	(99.9%)
11	93.1%	(101.2%)	(99.1%)	(75.6%)	(111.8%)	90.9%	(100.6%)
12 (one year)	95.0%	(99.7%)	(99.0%)	(76.9%)	(113.2%)	93.4%	(101.3%)
37	—	—	—	—	—	98.9%	(101.6%)
48 (four years)	—	—	—	—	—	(95.7%)	(98.7%)

*Notes*: Production capacity is set to 100% before a disaster (*t* = 0). Regarding H95Jan, because there is no data for the month prior to this earthquake (i.e., December 1994), the month before the disaster (*t* = 0) is set as May 1995 (the highest production capacity for the 12 months). Regarding IM08Jun, production capacity further decreases from the third month, probably because of the global financial crisis in 2008. See Supplementary Information Figs. S11–S12.

The production capacities associated with five of the earthquakes, excepting GEJE, are as follows. For H95Jan, production capacity is 84.5% when *t* = 1 (the worst). It recovers (100%) when *t* = 5 that is set to be the reference month; however, it then drops from the sixth month (97.5%) and does not recover to 100% again during the period. For N04Oct, production capacity is 95.8% (*t* = 1) and 95.6% (*t* = 2; the worst), and recovers to 99.4% when *t* = 7. For N07Jul, production capacity is 96.9% (*t* = 1) and recovers to 99.0% (*t* = 2). For IM08Jun, production capacity is 96.7% (*t* = 1) and 95.7% (*t* = 2; the worst). Interestingly, it drops to 92.4% (*t* = 3) and then more severely to 69.6% when *t* = 10, probably because of the global financial crisis in 2008. Thus, as a caveat, the model cannot separate the impact of two or more shocks occurring simultaneously (e.g., a disaster shock and a macroeconomic shock). Finally, regarding K16Apr, production capacity is 76.6% (*t* = 1) and 72.6% (*t* = 2; the worst), and recovers to 99.7% (*t* = 5). From these results, the peak of earthquake damage (to production) tends to occur in the first or second month after the earthquake. As the worst case, K16Apr induced a drop to 72.6% in the second month. Also, importantly, production capacity tends to temporarily recover between the fourth and eighth months after the disaster.

Damage attributable to GEJE differs substantively between FIMIC and non‐FIMIC prefectures. Regarding FIMIC, production capacity is lowest at 67.5% (*t* = 1), and indeed, this represents a greater impact on capacity than any of the other five earthquakes discussed above. It then gradually improves to 93.4% (*t* = 12; one year), and (almost) recovers fully, to 98.9%, in the 37th month (*t* = 37). Thus, the impact of the triple disaster is enormous, and the first temporal recovery took longer to occur than that for the above five earthquakes (mainly because of the damage in Fukushima). Meanwhile, regarding non‐FIMIC prefectures, production capacity is lowest at 86.8% (*t* = 1) and recovers to 100.5% (*t* = 8). Therefore, the speed of recovery in these prefectures was similar to that of the above five earthquakes.

### 5.2 Estimated Results

**Fig 4:**
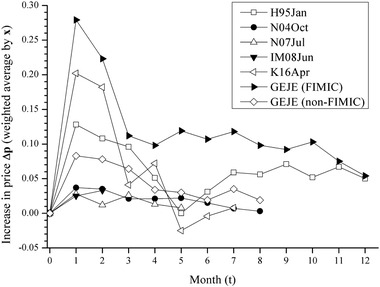
Increase in price for 12 months (initial price is one). *Notes*: Price is one at *t* = 0 and is the weighted average by initial production within the focal prefectures in the 54 manufacturing and mining sectors. See Table IV.

**Fig 5:**
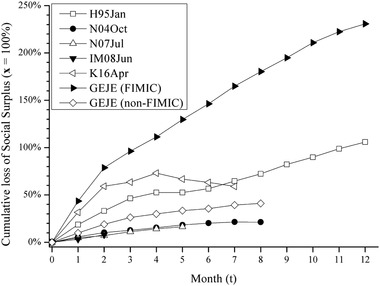
Cumulative loss of social surplus for 12 months (initial production is 100%). *Note*: See Tables V and VI and Supplementary Information Figs. S13–S17.

Tables IV and V show the estimated results of price, which is the weighted average by initial production within the focal prefectures in the 54 manufacturing and mining sectors, and the monthly damage for 12 months, and Table VI summarizes the peak and cumulative damage, which is also calculated only for the focal prefectures in the 54 sectors. In Tables IV and V, values in parentheses indicate the periods after the economy recovers temporarily. Figs. 4 and 5 plot the results of price and cumulative loss of SS, respectively (for other damage results, see Supplementary Information Figs. S13–S17).

**Table IV:** The Change in Output Price (Δp)

*t* (months)	H95Jan	N04Oct	N07Jul	IM08Jun	K16Apr	GEJE (FIMIC)	GEJE (non‐FIMIC)
1	0.128	0.037	0.028	0.025	0.202	0.279	0.083
2	0.108	0.035	0.012	0.033	0.182	0.223	0.078
3	0.096	0.021	0.026	(0.059)	0.041	0.112	0.064
4	0.051	0.021	0.013	(0.051)	0.072	0.098	0.034
5	0.000	0.022	0.008	(0.054)	−0.025	0.119	0.030
6	0.031	0.015	(0.003)	(0.116)	−0.004	0.107	0.019
7	0.059	0.007	(0.024)	(0.171)	0.008	0.118	0.035
8	0.056	0.003	(0.021)	(0.188)	(−0.059)	0.098	0.019
9	0.071	(0.014)	(0.026)	(0.214)	(−0.072)	0.092	(0.036)
10	0.052	(0.002)	(0.033)	(0.220)	(−0.086)	0.103	(0.021)
11	0.067	(−0.014)	(0.026)	(0.182)	(−0.117)	0.075	(0.015)
12 (one year)	0.050	(0.010)	(0.025)	(0.171)	(−0.111)	0.054	(0.013)
37	—	—	—	—	—	0.008	(0.007)
48 (four years)	—	—	—	—	—	(0.043)	(0.037)

*Notes*: Price is one at *t* = 0 and is the weighted average by initial production within the focal prefectures in the 54 manufacturing and mining sectors. Values are in parentheses after the first temporal recovery (except for IM08Jun; see Section 5). See Fig. 4.

**Table V:** The Losses of Social, Consumer, and Producer Surpluses (Compared With the Initial Monthly Production)

*t* (months)	H95Jan	N04Oct	N07Jul	IM08Jun	K16Apr	GEJE (FIMIC)	GEJE (non‐FIMIC)
Initial production (**x**) in	1.2T	0.4T	0.4T	0.5T	0.2T	3.1T	22.1T
manufacturing sectors (T JPY)	(100%)	(100%)	(100%)	(100%)	(100%)	(100%)	(100%)
Loss of social surplus (Δss)							
1 month	18.7%	5.7%	4.6%	3.1%	31.6%	43.5%	9.8%
2	14.3%	4.7%	2.4%	4.8%	27.5%	35.2%	9.2%
3	13.3%	2.3%	4.2%	(9.7%)	4.3%	17.5%	7.2%
4	6.2%	2.7%	2.9%	(8.1%)	9.5%	15.1%	3.7%
5	0.0%	2.9%	2.3%	(9.3%)	−6.3%	18.4%	3.4%
6	4.0%	2.0%	(1.4%)	(18.6%)	−3.5%	16.6%	2.1%
7	8.0%	1.1%	(4.7%)	(28.4%)	−4.2%	18.6%	3.8%
8	7.7%	−0.1%	(4.6%)	(30.9%)	(−16.5%)	15.4%	1.8%
9	9.9%	(1.3%)	(5.4%)	(35.0%)	(−16.2%)	14.5%	(3.7%)
10	7.6%	(−0.1%)	(7.0%)	(35.8%)	(−17.7%)	16.0%	(2.2%)
11	9.1%	(−2.6%)	(6.6%)	(30.1%)	(−24.8%)	11.5%	(1.5%)
12 (one year)	7.0%	(1.0%)	(5.9%)	(28.4%)	(−22.4%)	8.4%	(1.4%)
37	—	—	—	—	—	1.1%	(0.6%)
48 (four years)	—	—	—	—	—	(7.1%)	(4.5%)
Loss of consumer surplus (Δcs)							
1 month	21.1%	6.6%	5.1%	3.7%	33.0%	46.7%	9.5%
2	17.0%	5.7%	2.5%	5.6%	28.4%	37.7%	8.6%
3	15.4%	3.2%	4.6%	(10.8%)	4.3%	19.1%	7.1%
4	7.6%	3.5%	2.9%	(9.3%)	9.1%	16.7%	3.8%
5	0.0%	3.5%	2.2%	(10.3%)	−8.1%	20.4%	3.6%
6	4.9%	2.5%	(1.0%)	(21.0%)	−4.8%	18.5%	2.1%
7	9.3%	1.4%	(4.6%)	(31.6%)	−4.9%	20.6%	4.2%
8	8.9%	0.3%	(4.3%)	(34.2%)	(−19.5%)	17.2%	2.2%
9	11.3%	(2.0%)	(5.2%)	(38.4%)	(−19.9%)	16.1%	(3.7%)
10	8.8%	(0.2%)	(6.6%)	(39.2%)	(−22.9%)	18.0%	(2.3%)
11	10.5%	(−2.8%)	(6.4%)	(32.9%)	(−30.4%)	13.1%	(1.7%)
12 (one year)	8.0%	(1.5%)	(5.5%)	(30.9%)	(−25.7%)	9.5%	(1.6%)
37	—	—	—	—	—	0.8%	(0.2%)
48 (four years)	—	—	—	—	—	(7.2%)	(4.5%)
Loss of producer surplus (Δps)							
1 month	−2.4%	−0.9%	−0.5%	−0.6%	−1.4%	−3.3%	0.4%
2	−2.6%	−1.0%	−0.1%	−0.8%	−0.8%	−2.4%	0.6%
3	−2.0%	−0.9%	−0.5%	(−1.1%)	0.0%	−1.6%	0.1%
4	−1.4%	−0.7%	0.0%	(−1.2%)	0.4%	−1.6%	0.0%
5	0.0%	−0.7%	0.0%	(−1.1%)	1.8%	−2.0%	−0.1%
6	−0.8%	−0.5%	(0.3%)	(−2.4%)	1.3%	−1.9%	0.0%
7	−1.4%	−0.3%	(0.1%)	(−3.2%)	0.6%	−2.1%	−0.4%
8	−1.2%	−0.4%	(0.3%)	(−3.3%)	(3.1%)	−1.8%	−0.4%
9	−1.4%	(−0.7%)	(0.2%)	(−3.4%)	(3.7%)	−1.6%	(0.0%)
10	−1.2%	(−0.3%)	(0.3%)	(−3.4%)	(5.2%)	−2.0%	(−0.2%)
11	−1.4%	(0.2%)	(0.2%)	(−2.8%)	(5.5%)	−1.5%	(−0.2%)
12 (one year)	−1.0%	(−0.4%)	(0.4%)	(−2.6%)	(3.3%)	−1.2%	(−0.2%)
37	—	—	—	—	—	0.3%	(0.5%)
48 (four years)	—	—	—	—	—	(−0.1%)	(0.0%)

*Notes*: Initial monthly production (at *t* = 0) is 100%. Values are in parentheses after the first temporal recovery (except for IM08Jun). Note that these values are calculated only for the focal prefectures in the 54 sectors. See Fig. 5 for cumulative Δss and Supplementary Information Figs. S11–S15 for Δss, Δcs, and Δps.

**Table VI:** Values at the Peak and Cumulative Summation at the Temporal Recovery

	H95Jan	N04Oct	N07Jul	IM08Jun	K16Apr	GEJE (FIMIC)	GEJE (non‐FIMIC)
Initial production (**x**) in	1.2T	0.4T	0.4T	0.5T	0.2T	3.1T	22.1T
manufacturing sectors (T JPY)	(100%)	(100%)	(100%)	(100%)	(100%)	(100%)	(100%)
Δp at the peak	0.128	0.037	0.028	0.033	0.202	0.279	0.083
(The peak month)	(t = 1)	(t = 1)	(t = 1)	(t = 2)	(t = 1)	(t = 1)	(t = 1)
Δss at the peak (T JPY)	0.22T	0.02T	0.02T	0.03T	0.07T	1.35T	2.18T
(**x** = 100%)	(18.7%)	(5.7%)	(4.6%)	(4.8%)	(31.6%)	(43.5%)	(9.8%)
Δcs at the peak (T JPY)	0.25T	0.03T	0.02T	0.03T	0.07T	1.46T	2.10T
(**x** = 100%)	(22.1%)	(6.6%)	(5.1%)	(5.6%)	(33.0%)	(46.7%)	(9.5%)
Δps at the peak (T JPY)	−0.03T	−0.004T	−0.002T	−0.004T	−0.003T	−0.1T	0.08T
(**x** = 100%)	(−2.4%)	(−0.9%)	(−0.5%)	(−0.8%)	(−1.4%)	(−3.3%)	(0.4%)
(The temporal recovery)	(t = 12)	(t = 8)	(t = 5)	(t = 2)	(t = 7)	(t = 37)	(t = 8)
Cumulative Δss (T JPY)	1.25T	0.09T	0.07T	0.04T	0.13T	8.05T	9.11T
(**x** = 100%)	(105.9%)	(21.3%)	(16.4%)	(7.9%)	(59.0%)	(251.5%)	(41.1%)
Cumulative Δcs (T JPY)	1.45T	0.11T	0.07T	0.05T	0.13T	8.82T	9.1T
(**x** = 100%)	(122.9%)	(26.8%)	(17.4%)	(9.3%)	(57.0%)	(283.4%)	(41.1%)
Cumulative Δps (T JPY)	−0.2T	−0.02T	−0.004T	−0.01T	0.004T	−0.77T	0.01T
(**x** = 100%)	(−17.0%)	(−5.5%)	(−1.0%)	(−1.4%)	(1.9%)	(−24.8%)	(0.1%)

*Notes*: Price is one at *t* = 0 and is the weighted average by initial production within the focal prefectures in the 54 manufacturing and mining sectors. Initial monthly production (at *t* = 0) is 100%. See Tables IV and V.

Regarding the five earthquakes, the price peaks during the first month (the four cases) or second month (IM08Jun) (Fig. 4). H95Jan and K16Apr exhibit the worst increase in price (0.128 and 0.202), whereas N04Oct, N07Jul, and IM08Jun have a relatively small effect on the price by several percent (0.037, 0.028, and 0.033).

Tables V and VI and Fig. 5 represent the loss of SS (Δss) as the ratio of initial production, where the numerator is each of the losses, and the denominator is the initial production value only in the 54 manufacturing and mining sectors in the disaster area. The greatest damage at the peak (Δss) is 18.7% (i.e., 0.187 month‐production damage; 0.22T JPY) for H95Jan and 31.6% (0.07T JPY) for K16Apr. 0.22T JPY in H95Jan is larger than 0.07T JPY in K16Apr because the economic scale in Hyogo is larger than Kumamoto. In the other three cases, Δss at the peak is approximately 5% of initial production (5.7% [0.02T JPY] for N04Oct, 4.6% [0.02T JPY] for N07Jul, and 4.8% [0.03T JPY] for IM08Jun). When dividing Δss into losses of CS (Δcs) and PS (Δps), Δss consists almost of Δcs, and Δps tends to be small and even negative (i.e., profit). Specifically, Δcs at the peak are 0.25T JPY (22.1%) for H95Jan, 0.03T JPY (6.6%) for N04Oct, 0.02T JPY (5.1%) for N07Jul, 0.03T JPY (5.6%) for IM08Jun, and 0.07T JPY (33.0%) for K16Apr; similarly, Δps at the peak are −0.03T JPY (−2.4%) for H95Jan, −0.004T JPY (−0.9%) for N04Oct, −0.002T JPY (−0.5%) for N07Jul, −0.004T JPY (−0.8%) for IM08Jun, and −0.003T JPY (−1.4%) for K16Apr.

The damages are likely to converge until the eighth month at most. In Table VI, the cumulative loss of Δss until the temporal recovery is 105.9% (1.25T JPY) in H95Jan (*t* = 12) and 59.0% (0.13T JPY) in K16Apr (*t* = 7), meaning that the worst earthquake tends to have caused damage of over half a month's worth of production (i.e., more than 50%). The other three cases have lost SS (Δss) by approximately 10–20% until the temporal recovery (21.3% [0.09T JPY] for N04Oct when *t* = 8, 16.4% [0.07T JPY] for N07Jul when *t* = 5, and 7.9% [0.04T JPY] for IM08Jun when *t* = 2).

Meanwhile, GEJE increased the price by 0.279 in FIMIC prefectures at the peak (*t* = 1), which is larger than the worst earthquake (0.202 for K16Apr), and by 0.083 in non‐FIMIC prefectures (*t* = 1), which is slightly larger than N04Oct, N07Jul, and IM08Jun (Table V). The loss of SS (Δss) at the peak (Table VI) is 1.35T JPY (43.5%) in FIMIC (where Δcs is 1.46T JPY and Δps is −0.1T JPY) and 2.18T JPY (9.8%) in non‐FIMIC (where Δcs is 2.1T JPY and Δps is 0.08T JPY). In addition, the cumulative Δss due to GEJE (Table VI) is 8.05T JPY (251.5%) when *t* = 37 in FIMIC (where Δcs is 8.82T JPY and Δps is −0.77T JPY) and 9.11T JPY (41.1%) when *t* = 8 in non‐FIMIC (where Δcs is 9.1T JPY and Δps is 0.01T JPY).

### 5.3 Discussion

Here, the results are compared with those from Toyoda and Kouchi (1997) for H95Jan and Hayashi (2012) for GEJE (Supplementary Information Table S12). Regarding H95Jan, this study estimates that the damage of SS is 1.25T JPY (where Δcs is 1.45T JPY and Δps is –0.2T JPY). Meanwhile, Toyoda and Kouchi (1997) estimated that indirect damage for one year in the main damaged area in Hyogo was approximately 1,203.1B JPY for industrial sectors. Therefore, the loss of SS estimate from this study is 103.9% (= 1.25T/1.2031T) of the indirect damage estimation by Toyoda and Kouchi (1997); therefore, they are approximately equal.

Regarding GEJE, the loss of SS at the first temporary recovery is 7.83T JPY to FIMIC prefectures (*t* = 37) and 9.11T JPY to non‐FIMIC prefectures (*t* = 8). Therefore, the damage estimated herein is at least 16.94T JPY (= 7.83T+9.11T). Meanwhile, Hayashi (2012) suggested that the indirect damage of GEJE was approximately 100T JPY over 10 years. Roughly estimating the indirect damage incurred only by the industrial sectors gives approximately 24T JPY for 10 years because these sectors produced 24% of the total final demand in 2005. Therefore, the loss of SS estimate of this study is 70.6% (= 16.94T/24T) of the indirect damage estimated by Hayashi (2012), which suggests an underestimation. This is probably because of the target periods; this study estimates the first temporal recovery (37 and eight months), whereas Hayashi (2012) considers 10 years.

## 6 CONCLUSIONS

This study examines how to consider the supply constraint in IOA. Different from GPM in Park (2007), this study adopts LPM by introducing the price elasticity of demand, because of the following two points. First, LPM is for supply‐side analysis and is suited to the supply constraint. Second, LPM can handle “price” change (Dietzenbacher, 1997). In addition, this study proposes to use the loss of SS as the damage of supply constraint, because, unlike profit (margin), production (sales) does not identify how much damage is passed on to each supplier (upstream sector) and buyer (downstream sector).

Our methodology of the supply constraint is applied to the large earthquakes in Japan. Following the results, implications vis‐à‐vis countermeasures are as follows. The largest earthquakes tend to require economic assistance for 0.2–0.3 months (of initial production) immediately after a disaster within a damaged prefecture and more than 0.5 months (50% of initial production) in total until the first temporal recovery (the eighth month at most) to compensate for the loss of SS. Also, GEJE required twice as much (fast) economic assistance in the FIMIC prefectures than that required to address the damage caused by the largest earthquakes, which was at least 25 months in total (over 250% of initial production at the 37th month). Meanwhile, non‐FIMIC prefectures tended to incur approximately 0.4‐month damage (i.e., the loss of SS) until the eighth month. Note that our estimation is similar to (or slightly smaller than) the indirect damage estimation in the literature (Hayashi, 2012; Toyoda & Kouchi, 1997).

Our approach in LPM is straightforward to apply because it requires only a usual MRIO table and production capacity data, with simple assumptions (i.e., the input coefficients are invariant; the price elasticity of demand; and the linear functions of supply and demand). Note, however, that there are the following limitations. Because this study considered the supply constraint, the production capacity in the study is exogenous (based on past data). Therefore, this study does not predict when disaster damage will converge, and does not deal with simulating how to minimize the loss of SS (Supplementary Information D.2). For example, the IOA literature (e.g., Li et al., 2013) often examines these kinds of predictions and simulations, considering disaster countermeasures such as import and export policies, transfer policies among multiregions, inventory management, and so on. As another limitation, because production capacity is exogenous in the model, the impact of two or more simultaneous shocks cannot be separated (as in the case of IM08Jun). Nevertheless, as easily as the basic IOA, the supply constraint of this study can be applied to indirect damage predictions (or, the loss of SS) of potential catastrophes such as the Nankai Trough earthquakes [Nankai megathrust earthquakes] near Japan, which are anticipated to occur in the future (Investigative Commission for Nankai Trough Earthquake Model, 2012; Working Group on Countermeasures to the Nankai Trough Earthquake, 2012).

## Supporting information

Supplementary Material

Supplementary Material
