# Supplementary material for: Supply Constraint from Earthquakes in Japan in Input–Output Analysis
Source: Risk Anal. 2020 Jun 7;40(9):1811–30. doi: 10.1111/risa.13525 (PMC7818455; doi:10.1111/risa.13525)
Supplement: Supplementary file 2 — Supplementary Material [file RISA-40-1811-s002.pdf]

Supplementary Information

Abbreviations

Sections

- Supplementary Information A: Implausibility of GQM
- Supplementary Information B. Review of Park (2009)
- Supplementary Information C: Comparison between IOA, this study (and Park (2007)), and CGE
- Supplementary Information D. Applications to the approaches in the previous studies
  - D.1 Introduction: Comparison of the round effects in between LQM and LPM
  - D.2 Endogenous recovery for the survival coefficient
  - D.3 Sequential Interindustry Models (SIM) in Romanoff (1984) and Okuyama et al. (2004)
  - D.4 Impact on transportation networks in Sohn et al. (2004) and Kim et al. (2002)
  - D.5 Input-occupancy-output model in Chen (1990) and Chen et al. (2005)
  - D.6 Extension to the CGE model: the FIDELIO model in Kratena et al. (2013; 2017) and Kratena and Streicher (2017)
  - D.7 Spatial substitution and price multipliers: the FIDELIO model in Kratena and Streicher (2017)
  - D.8 The supply constraint in GQM
- Supplementary Information E: IIP and disaster damage
- Supplementary Information F: Indirect damages in H95Jan (Toyoda & Kouchi, 1997) and GEJE (Hayashi, 2012)

Figs.

- Fig. S1. Causal chain representation of the Ghoshian model (Oosterhaven, 1988, p.206, Fig. 1)
- Fig. S2. Economic loss estimation in Park (2009)
- Fig. S3. IO table in this study
- Fig. S4. Example of an IO table
- Fig. S5. Example of SAM
- Fig. S6. The round effect of LQM
- Fig. S7. The round effect of this study based on LPM
- Fig. S8. Overview of the main economic flows in FIDELIO (Kratena et al., 2013, Fig. 1.1, p.5)
- Fig. S9. Overview of selected prices in FIDELIO (Kratena et al., 2013, Fig. 1.2, p.10)
- Fig. S10. Direct damage of a disaster and indirect damage among sectors in a supply chain of five sectors (as an example)
- Fig. S11. Production capacity of H95Jan, N04Oct, N07Jul, IM08Jun, and K16Apr
- Fig. S12. Production capacity of GEJE
- Fig. S13. Loss of social surplus (initial production is 100%)
- Fig. S14. Loss of consumer surplus (initial production is 100%)
- Fig. S15. Loss of producer surplus (initial production is 100%)
- Fig. S16. Cumulative loss of consumer surplus (initial production is 100%)
- Fig. S17. Cumulative loss of producer surplus (initial production is 100%)

Tables

- Table S1. Summary of Data Necessary to Estimate Total Output Vectors (Park, 2009, Table 3, p.22)
- Table S2. Total Impacts via Price-sensitive Supply-driven USIO Model (Park, 2009, Table 4, p.23)
- Table S3. Comparison between IOA, this study, and CGE
- Table S4. Industry identification numbers
- Table S5. Industries of IIP for substitution numbers
- Table S6. Production (**x**) and final demand (**y**) at the monthly level in each prefecture (unit: B JPY)
- Table S7. Initial monthly production of each prefecture and initial social, consumer, and producer surpluses (unit: T JPY)
- Table S8. Production (**x**) and final demand (**y**) at the monthly level in 80 sectors (unit: B JPY)
- Table S9. Sample size of each IIP for production capacity
- Table S10. Descriptive statistics for JIP database
- Table S11. Regression results of the price elasticity of demand (ε)
- Table S12. Earthquake damage estimates from previous studies: H95Jan and GEJE

Abbreviations

- B: billion
- CGE: computable general equilibrium
- CS: consumer surplus
- FIDELIO: Fully Interregional Dynamic Econometric Long-term IO
- FIMIC: Fukushima, Iwate, Miyagi, Ibaraki, and Chiba
- GDP: gross domestic product
- GEJE: the Great East Japan Earthquake (GEJE)
- GPM: the Ghosh price model
- GQM: the Ghosh quantity model
- H95Jan: the Hyogo-ken Nanbu Earthquake in January 1995
- ICFM: integrated commodity flow model
- IIP: indices of industrial production
- IM08Jun: the Iwate-Miyagi Nairiku Earthquake in June 2008
- IO: input-output
- IOA: input-output analysis
- JIP: Japan Industrial Productivity
- JPY: Japanese yen
- K16Apr: the 2016 Kumamoto earthquakes in April 2016
- LPM: the Leontief price model
- LQM: the Leontief quantity model
- MB: million barrels
- MRIO: multi-regional input-output
- N04Oct: the Mid Niigata Prefecture Earthquake in October 2004
- N07Jul: the Niigata-ken Chuetsu-oki Earthquake in July 2007
- PS: producer surplus
- SAM: social accounting matrix
- SIM: Sequential Interindustry Model
- SS: social surplus
- T: trillion
- USD: U.S. dollar
- $/B: U.S. dollars per barrel

Supplementary Information A: Implausibility of GQM

In the IO literature, Oosterhaven (1988) has argued that GQM is implausible and has concluded that "both as a general description of the working of any economy and as a way to estimate the effects of loosening or tightening the supply of one scarce resource, the supply-driven model may not be used" (Oosterhaven, 1988, p.208). In GQM, the delivery from primary input ($\Delta\mathbf{v}$) to final demand ($\mathbf{y}$) is possible without any intermediate input, labor, and capital.

By using the Taylor expansion as in Oosterhaven (1988, Fig. 1 in p.206 and Eq.12 in p.207; Supplementary Information Fig. S1), GQM (Eq.10) is solved:

$$\mathbf{x}' = \mathbf{v}'\mathbf{I} + \mathbf{v}'\mathbf{B} + \mathbf{v}'\mathbf{B}^2 + \mathbf{v}'\mathbf{B}^3 + \cdots \quad \text{(S1)}$$

First, it is assumed the primary input is increased only in sector i (Δv_i) but not others (i.e., zero). The first term of the series ($\mathbf{v}'\mathbf{I}$) is the direct increase of this new production (e.g., extra input of $100 in sector i). Because it is direct, it is possible without any intermediate inputs. Next, the second term of the series ($\mathbf{v}'\mathbf{B}$) is the first round indirect (forward-linkage) production effects of the direct production effect in sector i. This means that the additional increase in production in sector i (e.g., $100 times **B**) is purchased by i and other sectors (as their inputs). Importantly, this is possible without any intermediate input and any increase in labor and capital. This interpretation applies to the third term ($\mathbf{v}'\mathbf{B}^2$) and others as well, and these round effects (i.e., endogenous forward production effect) are assumed to be finished simultaneously. Thus, these round effects are possible without any intermediate input, labor, and capital, and they will at last increase the final demand.

Supplementary Information B. Review of Park (2009)

This part reviews Park (2009; the unpublished paper). Park (2009) is helpful for understanding Park's (2007) approach and also beneficial as an example study of the price IO model in the U.S. oil industry because the price IO model has much fewer applications than the quantity model. Following Park's (2007) approach, Park (2009) examined the economic losses in the U.S. oil industry caused by two hurricanes (Katrina and Rita, which occurred in August 2005). After the two hurricanes hit the Gulf of Mexico coast (Louisiana and surrounding areas), the proportion of U.S. mining production in the state of Louisiana decreased from 11,090 million U.S. dollars (USD) in 2004 (10.23% of the U.S. total) to 9,569 million USD in 2005 (9.06% of the U.S. total) (i.e., it was changed by −13.72%). Park (2009) created the national IO table with 47 USC sectors (29 commodity sectors and 18 service sectors) and estimated the economic losses due to the damage in the U.S. oil industry for the four months after the hurricanes. Note that Park, Son, & Park (2017) recently examined the economic losses (job losses) caused by hurricane Sandy, which occurred in 2012, for U.S. industry. Park et al. (2017) also use the same 47 USC sectors, which are converted from the two-digit North American Industry Classification System sectors.

The model of Park (2009) is simplified as follows. From Eq.34 (A1 in Fig. 2), the price elasticity of demand in the oil industry (ε_{oil}) is defined as:

$$\varepsilon_{oil} = \frac{\Delta q_{oil}/q_{oil}}{\Delta p_{oil}/p_{oil}} \tag{B1}$$

Before the hurricanes, the base price of the oil industry (p_{oil}) is set as one, and the quantity of oil products (q_{oil}) is equal to production (x_{oil}). From Eq.35, using the exogenous price elasticity ($\bar{\varepsilon}_{oil}$), the price change is elastic to the quantity change as follows:

$$\Delta p_{oil} = \frac{\Delta q_{oil}/q_{oil}}{\bar{\varepsilon}_{oil}/p_{oil}} = \frac{\Delta q_{oil}}{q_{oil}\bar{\varepsilon}_{oil}} = \Delta q_{oil}\pi_{oil} \tag{B2}$$

where $\pi_{oil} = \frac{p_{oil}}{q_{oil}\bar{\varepsilon}_{oil}} = \frac{1}{q_{oil}\bar{\varepsilon}_{oil}}$. From Eqs. 22 and 38 (A3 in Fig. 2), the spillover change in price ($\Delta\tilde{\mathbf{p}}$) is calculated as:

$$\Delta\tilde{\mathbf{p}} = \mathbf{G}\Delta(\mathbf{D}\mathbf{p}_\mathbf{y}) = \mathbf{G}\hat{\mathbf{d}}[\Delta p_{oil}] = \mathbf{G}\hat{\mathbf{d}}[\Delta q_{oil}\pi_{oil}] \tag{B3}$$

$[\Delta p_{oil}]$ means the vector that takes Δp_{oil} in the oil industry and 0 in other industries. Note that Park (2007; 2009) calls the above equations the supply-driven model.

Table S1 shows a summary of the data necessary to estimate the total output vectors (Park, 2009,

Table 3). Note that this table can be calculated without the IO model. The quantity (q_{oil}) was originally expected to be 487.7 million barrels (MB), and the decrease (Δq_{oil}) was -140MB during the proceeding four months (i.e., it changed by -28.7%). The symbol "W" is the weight of each oil product, depending on the decrease proportion in quantity (Δq_{oil}). Note that the weighted average of the oil price before the hurricanes (based on W) would be 63.9 U.S. dollars per barrel ($/B). By using W and $\bar{\varepsilon}_{oil}$, Park (2009) calculated that the price increase (Δp_{oil}) was 5.1534 $/B (an increase of 8.06%). Note that Park (2009) refers to 5.1534 $/B as the total price-type direct losses on the U.S. oil market due to the two hurricanes. Park (2009) also estimated that the total loss in value was $721.5 million for four months, or 140MB (Δq_{oil}) times 5.1534$/B ($\Delta p_{oil}$) (Fig. S2).

Applying the data to the model, Table S1 (Park, 2009, Table 4) shows the estimated results in each of the 47 industries. Park (2009) estimated that the total price increased by 10.9780 $/B where 7.1545 $/B in the oil industry (i.e., USC sector 10: coal and petroleum products) plus 3.8235 $/B sums up the price changes in the other 46 USC industries. Thus, Park (2009) concluded that the total economic losses would be $1.54 billion (i.e., 11$/B times 140MB) (Fig. S2).

As mentioned in the main text, however, this study argues that Park (2009) has some interpretation issues. First, although Park (2007; 2009) calls the approach (Eqs. B1–B3) a supply-driven model, it seems indeed to be a demand-driven model (Section 3.2) because Eq.B3 is based on GPM (the demand-pull price model; Eqs.22 and 32), not on LPM (the cost-push price model; Eqs.15 and 27). Specifically, Δp_{oil} in Eq.B3 is the price change for the final output (final demand) in GPM, not for the value added in LPM. In other words, Eq.B3 treats the drop in 140MB of oil products not as a reduction in the primary inputs (supply) but as a reduction in buyers' purchases (demand). In addition, as in Section 3.5, when we follow Dietzenbacher (1997), GPM is a quantity model (Eqs.19–22) and therefore, cannot handle price changes. Indeed, unlike Dietzenbacher (1997), Eq.B3 treats the vector of price change $[\Delta p_{oil}]$.

Second, Park (2009, Table 4) summed up the price increases in each industry (Table S2). However, this study wonders whether the price vectors should not be summed up for the different industries. For example, suppose that two different industries increase total prices by 0.1 and 0.2 (10% and 20%), respectively. Park's (2009) approach sums them up to 0.3 (30%=10%+20%). Because the two industries do not necessarily have the same price unit, however, the value of 0.3 doesn't seem to make any sense.

Finally, suppose that the following estimation is correct: Δq_{oil} is 140MB, Δp_{oil} is 5.1534$/B,

and $\Delta\tilde{\mathbf{p}}$ is 10.9780 $/B. Even so, it seems strange to estimate the total value of losses as $721.5 million (5.1534$/B times 140MB) and the total economic losses as $1.54 billion (10.9780 $/B times 140 MB). Regarding the former, for buyers, 140MB were not purchased (or demanded), and only 347.7MB were indeed purchased at the 5.1534$/B higher price (Fig. S2); therefore, we wonder why the unpurchased 140MB would lose 5.1534$/B (in each deal). Similarly, regarding the latter, for sellers, 140MB were not produced (or supplied), and only 347.7 MB were produced at the 10.9780 $/B higher price (Fig. S2); therefore, we wonder why the non-produced 140 MB lost 10.9780 $/B in each production. In addition, it is important to note that 10.9780 $/B is not necessarily a loss for producers because some percentage of the 10.9780 $/B may be profitable for them (as value added or profit).

From another perspective (as in Section 3.3), we can compare the changes in production (sales) before and after the hurricanes. If there were no hurricanes, sales would have been $31.1 billion, or 487.7MB (q_{oil}) times 63.9$/B ($p_{oil}$). After the hurricanes (347.7MB), sales were $24 billion when the price increased by 5.1534$/B (347.7MB times 69.1$/B) and $26 billion when the price increased by 10.9780$/B (347.7MB times 74.9$/B). Thus, taking the difference without and with the hurricanes, the changes in sales are $−7,154 billion and $−5,129 billion. These values seem much higher than Park's (2009) damage estimate ($721.5 million and $1.54 billion) because the arc price elasticity of oil products is quite low (Section 3.3). After all, the quantity was changed by −28.7% (from 487.7MB to 347.7MB), but the price was increased only by 8.1% (+5.1534$/B from 63.9$/B) and 17.2% (+10.9780$/B). In other words, if the arc elasticity is stronger than 1.4 (i.e., 40.3% divided by –28.7%), sales could even be said to increase after the hurricanes. For example, when the price increases to 89.6 $/B (i.e., increased by 40.3%), the sales after the hurricanes would be the same as before (i.e., $31 billion).

References

Park, J., Son, M., & Park, C. (2017). Natural disasters and deterrence of economic innovation: a case of temporary job losses by Hurricane Sandy. *Journal of Open Innovation: Technology, Market, and Complexity*, *3*(1), 5. doi:10.1186/s40852-017-0055-2

Supplementary Information C: Comparison between IOA, this study (and Park (2007)), and CGE

This study briefly compares the common IOA, this study (and Park (2007)), and CGE (Supplementary Information Table S3). In IOA, price and quantity are usually independent of each other. IOA covers intermediate demand and input (as endogenous sectors), and final demand and value added (as exogenous sectors) (for IO table in this study, see Supplementary Information Fig. S3). The endogenous sectors balance supply and demand (hence, a square matrix) and are endogenous based on the technical coefficients (**A** or **B**). The exogenous sectors do not usually balance supply and demand (hence, it is not a square matrix). Because they are exogenous, labor and capital (as value added) are free to use, whereas household and export (etc.) in final demand are free to change. Also, the basic IOA requires only an IO table. Importantly, IOA is calculated by spreadsheet and has scalability in the calculation (i.e., it does not matter how many sectors are included).

This study (and Park (2007)) adopt IOA, assuming that price is elastic to quantity. The quantity of supply is exogenous (i.e., supply constraint), and the price is endogenous by using the price elasticity of demand. Thus, this study requires some parameters for the supply constraint and the price elasticity of demand. The other assumptions are the same as IOA.

Meanwhile, the CGE models are theoretically consistent. Price and quantity in CGE are theoretically elastic with each other. CGE can identify all economic activities by each of the utility functions. Instead of the IO table, CGE uses a social accounting matrix (SAM). Unlike the IO table, supply and demand in SAM are fully balanced in all economic sectors (hence, SAM is a square matrix; see Supplementary Information Figs. S4 for an example of the IO table and S5 for SAM).

CGE looks ideal for the disaster analysis, but it is harder to estimate than IOA. Regarding the data, CGE requires SAM. Hence, researchers have to create SAM, which covers a broader range than the IO table, and usually need to fit SAM to the CGE model. Also, it is necessary to prepare models (utility functions) and parameters for all activities to be consistent theoretically through the model. In addition, CGE is computable but highly non-linear and has no scalability, meaning that the more sectors there are, the more difficult it is to solve.

Supplementary Information D. Applications to the approaches in the previous studies

D.1 Introduction: The round effects of LQM and LPM

Some readers (including an anonymous reviewer) may wonder how this study relates to the methods proposed in previous studies. Thus, this section briefly discusses the following seven items: the endogenous recovery for the survival coefficient (Section D.2), the sequential interindustry models (SIM) in Romanoff (1984) and Okuyama et al. (2004) (D.3), the impact on transportation networks in Sohn et al. (2004) and Kim et al. (2002) (D.4), the input-occupancy-output model in Chen (1990) and Chen et al. (2005) (D.5), the extension to the CGE model: Fully Interregional Dynamic Econometric Long-term IO (FIDELIO) model in Kratena et al. (2013; 2017) and Kratena and Streicher (2017) (D.6), spatial substitution and price multipliers: the FIDELIO model in Kratena and Streicher (2017) (D.7), and the supply constraints in GQM (D.8).

Before coming to that, this part confirms once again that there is a difference between the LQM (the demand-driven quantity model) and this study based on LPM (the supply-driven price model) because most of the previous researches are based on LQM. First of all, regarding quantity, LQM is suitable for analyzing how the demand (final demand as output; $\mathbf{y}$) causes the supply (production as input; $\mathbf{x}$); however, going the opposite way (from supply to demand) is not suitable. Meanwhile, regarding price, LPM is suitable for analyzing how the supply (value added; $\mathbf{p_v}$) affects the demand ($\mathbf{p}$); similarly, however, going the opposite way (from demand to supply) is not suitable.

Opposite to GQM (from supply to demand, as in Fig. S1), LQM examines an economic effect from the demand (the right) to the supply (the left) only at the quantity level (see Fig. S6). By using the Taylor expansion as in Oosterhaven (1988), LQM (Eq.3) is solved:

$$\mathbf{x} = \mathbf{I}\mathbf{y} + \mathbf{A}\mathbf{y} + \mathbf{A}^2\mathbf{y} + \mathbf{A}^3\mathbf{y} + \cdots \tag{D1.1}$$

First, it is assumed the final demand (final output) is changed only in sector i (Δy_i) but not in others (i.e., zero). The first term of the series ($\mathbf{Iy}$) is the direct increase of this new demand (e.g., the extra output of \$100 in sector i). Next, the second term of the series ($\mathbf{Ay}$) is the first round indirect (backward-linkage) production effects of the direct production effect in sector i. This means that the additional increase in production in sector i (e.g., $\mathbf{A}$ times \$100) is required by i and other sectors as their outputs. This interpretation applies to the third term ($\mathbf{A}^2\mathbf{y}$) and others as well, and these round effects (i.e., the endogenous backward production

effect) are assumed to be finished simultaneously. Thus, they will at last increase the value added (e.g., n-th factor in sector j via the coefficient c_{nj}; Δv_{nj}).

Meanwhile, similarly to GQM, LPM examines an economic effect from the supply (the left) to the demand (the right) at the price level (i.e., the straight arrows in Fig. S7). Note that, as in the dotted arrows in Fig. S7, this study converts the quantity change to a price change (or vice versa), introducing the price elasticity of demand (Park, 2007). Specifically, first, suppose that a certain amount of quantity (as total input) in sector i decreases on the supply side ($\Delta x_i = \Delta q_i$). Note that, instead of Δq_i, Eq.36 uses the survival coefficient θ_i. By using the price elasticity (ε_i), Δq_i is converted to the price change for the value added (primary inputs) of the n-th factor (i.e., $\Delta(p_{vn}c_{ni})$; Eq.37) where c is the (fixed) coefficient for p_v.

By using the Taylor expansion as in Oosterhaven (1988), LPM (Eq.6) is solved:

$$\mathbf{p}' = \mathbf{I}(\mathbf{p}_{\mathrm{v}}'\mathbf{C}) + \mathbf{A}(\mathbf{p}_{\mathrm{v}}'\mathbf{C}) + \mathbf{A}^2(\mathbf{p}_{\mathrm{v}}'\mathbf{C}) + \mathbf{A}^3(\mathbf{p}_{\mathrm{v}}'\mathbf{C}) + \cdots \quad \text{(D1.2)}$$

where **C** is the matrix of the coefficient c_{ni}. Similarly to Eq.D1.1, it is assumed the price of value added (primary inputs) is increased only in sector i ($\Delta p_{vn}c_{ni}$) but not others (i.e., zero). The first term of the series ($\mathbf{I}(\mathbf{p}_{\mathrm{v}}'\mathbf{C})$) is the direct increase of this new supply (e.g., extra price margin of one cent per $1 [1%] in sector i). Next, the second term of the series ($\mathbf{A}(\mathbf{p}_{\mathrm{v}}'\mathbf{C})$) is the first round indirect (forward-linkage) production effects of the direct production effect in sector i. This means that the additional increase in price margin in sector i (e.g., **A** times one cent per $1) is required by i and other sectors (as their input price). This interpretation applies to the third term ($\mathbf{A}^2(\mathbf{p}_{\mathrm{v}}'\mathbf{C})$) and others as well, and these round effects (i.e., the endogenous forward production effect) are assumed to be finished simultaneously. Thus, they will at last change the price of total outputs and the final outputs (e.g., m-th factor in sector j via the coefficient d_{mj}; $\Delta p_{ym}d_{mj}$).

D.2 Endogenous recovery for the survival coefficient

As noted in the conclusions (Section 6), the production capacity in the study is exogenous (based on past data). Therefore, this study does not predict when the disaster damage will converge. However, some readers may wonder how to consider the notion of resilience in the supply in an endogenous way.

Perhaps one of the simplest ideas is to consider the t-period sequential model, assuming that the final output (or the survival coefficient) recovers and depending on the realized final demand in the previous period. Let Δq_i^t be the quantity change and θ_i^t be the survival rate both in the period t. Also, let $\tilde{q}_i^t$ be the

realized final output (final demand), which is the pre-disaster quantity (q_i) plus the spillover change in quantity ($\Delta\tilde{q}_i^t$). From Eq.36, the change ratio of the quantity at t is expressed:

$$\frac{\Delta q_i^t}{q_i} = \theta_i^t - 1 \quad \text{(D2.1)}$$

Here, suppose that the survival coefficient recovers, depending on the realized final outputs. For example, a producer may not be able to carry out production activities without obtaining the required final outputs. Or, if too many final outputs are supplied in the market, some suppliers may adjust their production. Thus, in the endogenous way of thinking, suppose that the quantity change at period t+1 (Δq_i^{t*1}, or θ_i^{t+1}) is a function of the realized final demand ($\tilde{q}_i^t$, or $\Delta\tilde{q}_i^t$) at the previous period t.

$$\frac{\Delta q_i^{t+1}}{q_i} = \theta_i^{t+1} - 1 = f(\tilde{q}_i^t) \quad \text{(D2.2)}$$

where $f(\cdot)$ denotes some endogenous function. Note that in this case we need to substitute only the initial value at t=0 (i.e., Δq_i^1, or θ_i^1) and do not need to substitute after t=1.

D.3 Sequential Interindustry Models (SIM) in Romanoff (1984) and Okuyama et al. (2004)

SIM was proposed by Romanoff (1984) and his collaborators mainly during the 1980s (for a short history, see Okuyama et al., 2004). SIM is a model that takes into account a shift in production timing. As the background, a basic LQM (Eq.3) assumes that an additional amount of final demand (y) would induce the corresponding production (x) instantly. For example, Eq.D1.1 above (Fig. S6) assumes that the round effect finishes simultaneously and instantly. This assumption is not usually realistic, however, because new production would require lead and delivery times, depending on the existing amount of inventory. In particular, a disaster often causes disruptions in production (the supply constraint), but the timing of the disruptions may not affect all sectors equally.

Following Romanoff (1984), LQM (Eq.1) is converted to a "t-period" static model where t means discrete intervals of equal duration (e.g., month or week):

$$\mathbf{x}_t = \mathbf{z}_t + \mathbf{y}_t \quad \text{(D3.1)}$$

Given **A** is fixed, suppose delivery of intermediate output (z) at t is linked to the production at t+1 because of production time:

$$\mathbf{z}_t = \mathbf{A}\mathbf{x}_{t+1} \quad \text{(D3.2)}$$

Thus, the t-period model is expressed as:

$$\mathbf{x}_t = \mathbf{A}\mathbf{x}_{t+1} + \mathbf{y}_t \tag{D3.3}$$

Eq.D.3.3 continues indefinitely (to t+∞) because $\mathbf{x}_{t+1}$ is nested in the t-period equation. By using the double-sided Z transform, Eq.D3.3 is solved as:

$$\mathbf{x}_t = \mathbf{y}_t + \mathbf{A}\mathbf{y}_{t+1} + \mathbf{A}^2\mathbf{y}_{t+2} + \mathbf{A}^3\mathbf{y}_{t+3} + \cdots = \sum_{r=0}^{\infty} \mathbf{A}^r\, \mathbf{y}_{t+r} \tag{D3.4}$$

Eq.D3.4 is called a core SIM of the original version, meaning that the current total production (at t) is expressed as "a power series of future series final demand orders" (Romanoff, 1984, p.354).

More realistic models have been proposed as modified SIM rather than the core SIM. The SIM literature usually considers two types of production modes: the anticipatory production mode and the responsive production mode. The former makes readymade standard products and prepares product inventories. It delivers the final outputs when production is complete. Meanwhile, the latter will make non-standard, unique products without preparing inventories. Hence, before the production is complete, it takes a lead time and production interval to deliver the final outputs.

For example, Okuyama et al. (2004) formulated the two production modes as follows. The anticipatory production mode is expressed as:

$$\mathbf{x}_t = \mathbf{A}\mathbf{x}_\sigma + \mathbf{u}_t + \mathbf{y}_t \tag{D3.5}$$

where $\mathbf{u}$ is (a vector of) the outputs to product inventories. Here, t is time interval of input application, and σ is time interval of production completion. Here, as an assumptions, the intermediate output (**Ax**) is priced at σ (i.e., product completion), not at t (i.e., input application). Meanwhile, the responsive production mode is represented as:

$$\mathbf{x}_t = \mathbf{A}\mathbf{x}_{\sigma-h-k} + \mathbf{y}_t \tag{D3.6}$$

where k is the ordering lead time, and h is the production interval. Note that Eq.D3.6 has no product inventories. Again, as an assumption, the intermediate output (**Ax**) is priced at $(\sigma - h - k)$ (i.e., the initial ordering time), not at t (i.e., input application). Thus, the combined anticipatory-responsive production model encompasses both properties of Eqs.D3.5–D3.6 as:

$$\mathbf{x}_t = \mathbf{A}\mathbf{x}_{\sigma-h-k} + \mathbf{u}_t + \mathbf{y}_t \tag{D3.7}$$

The above SIM is based on LQM, and hence, we consider rewriting SIM in terms of the LPM version as follows. First of all, LQM considers the backward linkage from demand to supply (i.e., from

output to input). For example, the production ($\mathbf{x}$) itself is priced at t (input), whereas the intermediate outputs ($\mathbf{Ax}$) are priced when they are demanded (or ordered; output), such as t+1 in Eq.D3.3, σ in Eq.D3.6, and $(\sigma - h - k)$ in Eqs.3.6–3.7. Thus, in SIM, the current input price is decided by the future output price (Eq.D3.4). Meanwhile, LPM (this study) considers the forward linkage from supply to demand (i.e., from input to output). Therefore, contrary to the normal SIM, in LPM, the future output price should be decided by the current input prices.

Specifically, we convert the original core SIM (Eqs.D3.1–D3.4) as follows. First, LPM (Eq.4) is converted to a t-period static model (similarly to Eq.D3.1):

$$(\mathbf{p}')_t = (\mathbf{p}'_\mathrm{z})_t + (\mathbf{p}'_\mathrm{v}\mathbf{C})_t \tag{D3.8}$$

where $\mathbf{p}'_\mathrm{z}$ is the price vector of intermediate inputs (z), and $(\cdot)_t$ means a period t. For simplicity, suppose that $\mathbf{C}$ may be variable at t. Given that $\mathbf{A}$ is fixed, suppose that the price for intermediate inputs at t is linked to the production at t–1 because of production time:

$$(\mathbf{p}'_\mathrm{z})_t = (\mathbf{p}')_{t-1}\mathbf{A} \tag{D3.9}$$

Thus, the t-period model is expressed as:

$$(\mathbf{p}')_t = (\mathbf{p}')_{t-1}\mathbf{A} + (\mathbf{p}'_\mathrm{v}\mathbf{C})_t \tag{D3.10}$$

Eq.D.3.10 continues negatively indefinitely (to t–∞) because $(\mathbf{p}')_{t-1}$ is nested in the t-period equation. By using the double-sided Z transform, Eq.D3.10 is solved as:

$$(\mathbf{p}')_t = (\mathbf{p}'_\mathrm{v}\mathbf{C})_t + \mathbf{A}(\mathbf{p}'_\mathrm{v}\mathbf{C})_{t-1} + \mathbf{A}^2(\mathbf{p}'_\mathrm{v}\mathbf{C})_{t-2} + \mathbf{A}^3(\mathbf{p}'_\mathrm{v}\mathbf{C})_{t-3} + \cdots = \sum_{r=0}^{\infty} \mathbf{A}^r\, \mathbf{y}_{t-r} \tag{D3.11}$$

This part refers Eq.D3.11 to the original core SIM of the LPM version, meaning that the current total price (at t) is expressed as a power series of past series the price of primary inputs (to t–∞) have induced.

In addition, analogously to Eqs.D3.5–D3.7, we may consider the combined anticipatory-responsive production model for the LPM version as follows. As the simplest idea, we suppose that the correspondence between the time intervals of input (e.g., supply at t) and output (e.g., demand at σ) can be reversed from LQM, as in Okuyama et al. (2004), because LPM is the supply-driven model. For example, the anticipatory production mode may be expressed as:

$$(\mathbf{p}')_\sigma = (\mathbf{p}')_t\mathbf{A} + (\mathbf{p}'_\mathrm{v}\mathbf{C})_\sigma \tag{D3.12}$$

where t is time interval of input application, and σ is time interval of production completion. It is assumed that prices for total outputs $(\mathbf{p}')$ and primary inputs $(\mathbf{p}'_\mathrm{v}\mathbf{C})$ are priced at σ (when production is complete),

and the price for intermediate inputs $(\mathbf{p}'\mathbf{A})$ is priced at t (when input is applied). Note that Eq.D3.12 ignores the inventory price, assuming that the inventory is priced at σ (at the completion of production).

Meanwhile, the responsive production mode of the LPM version may be expressed as:

$$(\mathbf{p}')_{\sigma-h-k} = (\mathbf{p}')_t\mathbf{A} + (\mathbf{p}'_{\mathrm{V}}\mathbf{C})_{\sigma-h-k} \tag{D3.13}$$

where k is the ordering lead time and h is the production interval. It is assumed that prices for total outputs $(\mathbf{p}')$ and primary inputs $(\mathbf{p}'_{\mathrm{V}}\mathbf{C})$ are priced at $(\sigma - h - k)$ because production will take place after ordering at $(\sigma - h - k)$. Note that Eq.D3.13 is also considered as the combined anticipatory-responsive production model for LPM because it already encompasses the property of Eq.3.12.

D.4 Impact on transportation networks in Sohn et al. (2004) and Kim et al. (2002)

The production network is a key issue in the literature on disasters. As a study of this challenging issue, this section would like to review Sohn et al. (2004), who examined the economic impacts of an earthquake on transportation. The authors analyzed the period from the base year (1993) to 2017 (as a forecast) in 36 earthquake analysis zones in 13 economic sectors in the United States. For background, in recent decades, supply chains have become more developed (not only domestically but also globally), probably because the costs of transfers and transportation have become much lower. In other words, transportation intensity (or transportation efficiency) in the supply chain network has increased a lot, leading to an increase in the fragmentation of production. However, the efficiency here relies on eliminating wasteful networks, which can result in a loss of resilience. Therefore, when a disaster disrupts the supply chains, the more efficient the production network, the greater the damage to production.

The model of Sohn et al. (2004) is divided into two parts. One part is the integrated commodity flow model (ICFM) (Sohn et al., 2004, Section 12.6, pp.247–249), which was proposed in Kim et al. (2002, Eqs.1–8 in Section 3, pp.226–229) and related papers. Note that ICFM is based partly on but far from the ordinary IO models, and therefore, this section does not fully review ICFM from the viewpoint of IOA. The other is the final demand loss function (Sohn et al., 2004, Section 12.5, pp.242–247), which is based on LQM.

Specifically, ICFM seeks to find the total transportation costs in the economic system, which are the network assignment costs, intraregional travel costs, and interregional flow distribution costs. This estimate is subject to the following three conditions: material balance, conservation of flow, and non-

negativity of the flow (of the output of sector). Sohn et al. (2004, Eq.12.17, p.249) calculated the change in a sectoral system-wide transportation cost (ΔTC_i) in sector i.

Meanwhile, regarding the final demand loss function (Sohn et al., 2004, Eq.12.8, p.242), we can simplify it as follows. First of all, the loss of final demand ($\Delta\mathbf{y}$) is based on LQM as:

$$\mathbf{y} = (\mathbf{I} - \mathbf{A})\mathbf{x} \Leftrightarrow \Delta\mathbf{y} = (\mathbf{I} - \mathbf{A})\Delta\mathbf{x} \quad \text{(D4.1)}$$

given that $\mathbf{A}$ is fixed. As the steps for estimation, we first estimate the transportation cost (ΔTC_i) by ICFM and the corresponding loss of production ($\Delta\mathbf{x}$). Eq.D4.1 then estimates the loss of final demand ($\Delta\mathbf{y}$) as the economic loss. Specifically, in a manner similar to the survival coefficient (θ) in the main text, $\Delta\mathbf{x}$ is caused by the network disruption (coefficient):

$$\Delta\mathbf{x} = \mathbf{N}\otimes(\mathbf{I} - \mathbf{R}) \circ \mathbf{x} \quad \text{(D4.2)}$$

$\mathbf{N}$ is the matrix of network disruption ratio (by zone) (i.e., the symbol $\mathbf{D}$ is used in Sohn et al. (2004), but we use $\mathbf{N}$ to distinguish it from the other coefficient $\mathbf{D}$ in the main text). $\mathbf{R}$ is the matrix of sectoral resiliency factor, and thus, $(\mathbf{I} - \mathbf{R})$ represents a negative factor of sectoral resiliency. $\otimes$ means the multiplier of tensor product. "∘" denotes the multiplier of Hadamard product (i.e., the element-wise product). Here, the network disruption is represented as the network disruption ratio ($\mathbf{N}$) times the sectoral resiliency ratio $(\mathbf{I} - \mathbf{R})$, meaning that the greater the network disruption and the lower the resilience, the greater the damage to production. Recall that $\mathbf{x}$ is $(\mathbf{I} - \mathbf{A})^{-1}\mathbf{y}$, and the final demand loss function of Sohn et al. (2004) is derived as:

$$\Delta\mathbf{y} = (\mathbf{I} - \mathbf{A})\{[\mathbf{N}\otimes(\mathbf{I} - \mathbf{R})] \circ \mathbf{x}\} = (\mathbf{I} - \mathbf{A})\{[\mathbf{N}\otimes(\mathbf{I} - \mathbf{R})] \circ [(\mathbf{I} - \mathbf{A})^{-1}\mathbf{y}]\} \quad \text{(D4.3)}$$

This part reviews the model of Sohn et al. (2004). First of all, ICFM itself will be helpful for examining the network in each region when considering each material flow. It is also beneficial for calculating the transportation costs (ΔTC).

Meanwhile, regarding the final demand loss function, this part wonders if it is inconsistent with the IOA theory. The strangest point is that Eq.D4.1 (LQM) calculates the demand loss ($\Delta\mathbf{y}$) by the production loss ($\Delta\mathbf{x}$) although LQM is the demand-driven model. Instead, we rewrite the final demand loss function for the supply-driven model as follows.

The simplest idea changes Eq.D4.2 to the quantity equation, analogously to the survival coefficient (θ). For simplicity, before a disaster, we assume that production $(\mathbf{x})$ equals quantity $(\mathbf{q})$ at price $(\mathbf{p})$ is

one. First, suppose that the network disruption causes the supply constraint, and Eq.D4.2 is rewritten to the quantity level:

$$\Delta\mathbf{q} = \mathbf{N} \otimes (\mathbf{I} - \mathbf{R}) \circ \mathbf{q} \quad \text{(D4.4)}$$

Thus, $\mathbf{N} \otimes (\mathbf{I} - \mathbf{R})$ is analogous to the survival coefficient (θ). Thus, we can obtain the quantity loss in a certain sector i (Δq_i) and substitute it to Eq.35.

Rather, suppose that we already know how much the additional transportation cost (ΔTC) will be after a disaster (e.g., via ICFM). Usually, however, ΔTC is value added only in the transport sector and is an intermediate input in the other sectors. Therefore, separately, the price of value added is increased by ΔTC divided by q in the transport sector as follows:

$$\Delta(p_v' c) = \frac{\Delta TC}{x} = \frac{\Delta TC}{q} \text{ in the transport sector} \quad \text{(D4.5)}$$

Meanwhile, the price of intermediate input is increased by ΔTC divided by **Ax** in other sectors as follows:

$$\Delta(p' a) = \frac{\Delta TC}{ax} \text{ in other sectors except for the transport sector} \quad \text{(D4.6)}$$

In this way, we can use the information on the additional prices of value added $(\mathbf{p}_v' \mathbf{C})$ and the intermediate inputs $(\mathbf{p}' \mathbf{A})$ in IOA. For example, we may consider updating the coefficients **A** to $\mathbf{A}_{\mathbf{new}}$, or **C** to $\mathbf{C}_{\mathbf{new}}$.

D.5 Input-occupancy-output model in Chen (1990) and Chen et al. (2005)

Usually, LQM (the Leontief production function) assumes a fixed proportion of inputs (i.e., the fixed **A**), meaning that one product (one output) requires a fixed proportion of inputs. In other words, LQM does not consider capital assets such as the plant, land, labor, and other forms. However, this assumption may not be realistic because, without capital assets, no matter how many inputs, no output can be produced. For example, in a car supply chain, a car (output) is usually made up of tens of thousands of parts (inputs). Now, suppose that a disaster damages a plant that creates a key automobile part that cannot be replaced or substituted in order to complete a car. As a result, car production would not be complete just because the plant cannot operate.

As a model for considering such a capital (or asset) constraint, the input-occupancy-output model (or, the IO model with assets) was proposed in Chen (1990) and Chen et al. (2005). The term "occupancy" means "holding and using assets at a point of time by a sector, where assets include fixed assets, inventory, financial assets, labor, natural resources, and so on" (Chen et al., 2005, p.224). Chen et al. (2005, p.213)

explained assets in terms of the following three features: "assets consist not only of fixed assets (such as machinery and construction), but also of inventories, financial assets, labor force (educated or not, skilled or not), natural resources, intangible assets, and others;" where "(1) assets are a prerequisite for input and output...; (2) assets are related with output and input...; and (3) input is dependent on the assets used."

Regarding the normal LQM, which does not consider capital assets, Chen et al. (2005, Eqs.8–9, p.218) first rewrote LQM as follows:

$$\mathbf{x} = \mathbf{Ax} + \mathbf{y} = (\mathbf{I} - \mathbf{A})^{-1}\mathbf{y} = (\mathbf{A_B} + \mathbf{I})\mathbf{y} \quad \text{(D5.1)}$$

$$\text{where } \mathbf{A_B} = (\mathbf{I} - \mathbf{A})^{-1} - \mathbf{I} \quad \text{(D5.2)}$$

Instead of **B** in Chen et al. (2005), we use $\mathbf{A_B}$ to distinguish it from the coefficient **B** in the main text. Notice that compared to the Leontief inverse **L**, $\mathbf{A_B}$ ignores the identity matrix **I**:

$$\mathbf{L} = (\mathbf{I} - \mathbf{A})^{-1} = \mathbf{I} + \mathbf{A} + \mathbf{A^2} + \mathbf{A^3} + \cdots \quad \text{(D5.3)}$$

$$\mathbf{A_B} = (\mathbf{I} - \mathbf{A})^{-1} - \mathbf{I} = \mathbf{A}(\mathbf{I} - \mathbf{A})^{-1} = \mathbf{A} + \mathbf{A^2} + \mathbf{A^3} + \cdots \quad \text{(D5.4)}$$

Here, Chen et al. (2005, p.218) considered to include the indirect "consumption" of fixed assets, rewriting $\mathbf{A_B}$ to $\mathbf{A_B^*}$ as follows:

$$\mathbf{A_B^*} = \mathbf{A} + \mathbf{A_B^*}\mathbf{A} + \widehat{\alpha}\mathbf{A_D} + \mathbf{A_B^*}\widehat{\alpha}\mathbf{A_D} = (\mathbf{A} + \widehat{\alpha}\mathbf{A_D}) + \mathbf{A_B^*}(\mathbf{A} + \widehat{\alpha}\mathbf{A_D}) \quad \text{(D5.5)}$$

Note that, instead of **D** in Chen et al. (2005), we use $\mathbf{A_D}$ to distinguish it from the coefficient **D** in the main text. $\mathbf{A_D}$ is $I \times I$-matrix with the fixed asset holding coefficient. $\widehat{\alpha}$ is the diagonal matrix of the depreciation rate α_i. In the middle part of Eq.D5.5, the first term $(\mathbf{A})$ means the direct consumption coefficient via intermediate inputs; the second term denotes $(\mathbf{A_B^*}\mathbf{A})$ the indirect consumption via intermediate inputs; the third term $(\widehat{\alpha}\mathbf{A_D})$ denotes the direct consumption via fixed assets, which is the product of the depreciation rate $(\widehat{\alpha})$ and the fixed asset holding coefficient $(\mathbf{A_D})$; and the fourth term $(\mathbf{A_B^*}\widehat{\alpha}\mathbf{A_D})$ refers to the indirect consumption via fixed assets. Notice that Eq.D5.5 has round (or ripple) effects because of including $\mathbf{A_B^*}$ in the two terms in the middle part. The third part shows that $\mathbf{A_B^*}$ is divided into the direct consumption coefficients $(\mathbf{A} + \widehat{\alpha}\mathbf{A_D})$ and the indirect ones $\mathbf{A_B^*}(\mathbf{A} + \widehat{\alpha}\mathbf{A_D})$.

Note that we can rewrite Eq.D5.5 as follows:

$$\mathbf{A_B^*}\{\mathbf{I} - (\mathbf{A} + \widehat{\alpha}\mathbf{A_D})\} = \mathbf{A} + \widehat{\alpha}\mathbf{A_D} \quad \text{(D5.6)}$$

Or, equivalently, using Eq.D5.4:

$$\mathbf{A_B^*} = (\mathbf{A} + \widehat{\alpha}\mathbf{A_D})\{\mathbf{I} - (\mathbf{A} + \widehat{\alpha}\mathbf{A_D})\}^{-1} = \{\mathbf{I} - (\mathbf{A} + \widehat{\alpha}\mathbf{A_D})\}^{-1} - \mathbf{I} \quad \text{(D5.7)}$$

Chen et al. (2005, Eqs.15–16, p.220) compared the normal LQM with their approach, replacing $\mathbf{A}$ with $(\mathbf{A}+\widehat{\alpha}\mathbf{A_D})$ as follows:

$$\mathbf{x}=(\mathbf{A}+\widehat{\alpha}\mathbf{A_D})\mathbf{x}-\widehat{\alpha}\mathbf{A_D}\mathbf{x}+\mathbf{y}=(\mathbf{A}+\widehat{\alpha}\mathbf{A_D})\mathbf{x}+\mathbf{y}^* \tag{D5.8}$$

where $\mathbf{y}^*$ represents the vector of net final demands, excluding the replacement investments of fixed assets $(-\widehat{\alpha}\mathbf{A_D}\mathbf{x})$. Eq.D5.8 considers the consumption via fixed assets as the intermediate outputs $(\widehat{\alpha}\mathbf{A_D}\mathbf{x})$ but removes the same amount of consumption from the final demand $(-\widehat{\alpha}\mathbf{A_D}\mathbf{x})$ because of equality. Thus, we can estimate that the additional net final demand ($\mathbf{y}^*$) would increase how much in terms of production as follows:

$$\mathbf{x}=\{\mathbf{I}-(\mathbf{A}+\widehat{\alpha}\mathbf{A_D})\}^{-1}\mathbf{y}^* \tag{D5.9}$$

From here, as the simplest application, we explain how the model of this study (i.e., LPM) can integrate the input-occupancy-output model. First, considering the coefficient via fixed assets ($\widehat{\alpha}\mathbf{A_D}$), we rewrite the Leontief inverse $\mathbf{L}$ to $\mathbf{L}^*$ as follows:

$$\mathbf{L}^*=\{\mathbf{I}-(\mathbf{A}+\widehat{\alpha}\mathbf{A_D})\}^{-1} \tag{D5.10}$$

Note that $\widehat{\alpha}\mathbf{A_D}$ may take large values if a disaster causes a huge amount of damage to the fixed assets. Thus, replacing $\mathbf{L}$ in Eq.45 with $\mathbf{L}^*$, we represent the price of total outputs plus the unit cost of occupying fixed assets $(\Delta\widetilde{\mathbf{p}}'^*)$ as follows:

$$\Delta\widetilde{\mathbf{p}}'^*=\Delta(\mathbf{p}_\mathrm{v}'\mathbf{C})\mathbf{L}^*=\Delta\mathbf{p}'\hat{\mathbf{c}}\mathbf{L}^*=\left[\frac{\Delta q_i}{q_i\varepsilon_i}\right]'\hat{\mathbf{c}}\mathbf{L}^*=\left[\frac{\theta_i-1}{\varepsilon_i}\right]'\hat{\mathbf{c}}\mathbf{L}^* \tag{D5.11}$$

In this way, we can examine how the change in price for primary inputs ($\Delta(\mathbf{p}_\mathrm{v}'\mathbf{C})$) will affect the price of total outputs with the unit cost of occupying fixed assets $(\Delta\widetilde{\mathbf{p}}'^*)$.

D.6 Extension to the CGE model: the FIDELIO model in Kratena et al. (2013; 2017) and Kratena and Streicher (2017)

The model of this study is just an IOA, not CGE, but some readers may wonder how this study can be applied to CGE. Thus, this part briefly discusses it with reference to Fully Interregional Dynamic Econometric Long-term IO Model (FIDELIO) (Kratena et al., 2013; 2017; Kratena & Streicher, 2017). FIDELIO is very similar to CGE and has a demand-driven and linear "IO philosophy" (Kratena et al., 2013). Regarding the overview of FIDELIO, Fig. S8 (Kratena et al., 2013, Fig. 1.1, p.5) shows the main economic

flows (i.e., monetary transactions, not real [quantity] flows), and Fig. S9 (Kratena et al., 2013, Fig. 1.2, p.10) indicates the selected prices.

Skipping the details, regarding the disaster analysis, FIDELIO can analyze both demand and supply constraints (i.e., shocks) because it includes the CGE essence but is more suitable for the demand constraint than the supply constraint because of following LQM (as the demand-driven model). At the middle of top of Fig. S8, $GD_{bp}(r; g; u)$ represents demand by user u for good g domestically produced in region r at basic prices (bp). As in LQM, $GD_{bp}(r; g; u)$ derives the supply of goods (gross outputs) by sector s in region r (denoted by Q(r; s)), under the constant proportions of market share (denoted by MKSH(r; g; s)) at the base year (t=0 as in the superscript). Kratena et al. (2013, Eqs.4.1–4.2, pp.70–71) express this relationship as follows:

$$\mathrm{MAKE}(r, g, s) = \underline{\mathrm{MKSH}^0}(r, g, s) \cdot \sum_u \mathrm{GD}_{bp}(r, g, u) \tag{D6.1}$$

$$\mathrm{Q}(r, s) = \sum_g \mathrm{MAKE}(r, g, s) \tag{D6.2}$$

where MAKE (r, g, s) denotes the total supply of good g by sector s in r (i.e., make matrix element). Notice that Eqs.D6.1–D6.2 are analogous to LQM (Eq.3) as:

$$\mathbf{x} = (\mathbf{I} - \mathbf{A})^{-1}\mathbf{y} = \mathbf{L}\mathbf{y} \tag{3}$$

where $\mathrm{GD}_{bp}(r, g, u)$ is analogous to $\mathbf{y}$. Thus, FIDELIO can analyze the demand constraint (shock) via $\Delta\mathrm{GD}_{bp}(r, g, u)$, in a manner similar to $\Delta\mathbf{y}$ in LQM.

Regarding the price, FIDELIO "distinguishes between prices at a very detailed level," and "all prices ultimately derive from output prices PQ(r,s), which are basic prices determined in the translog production block using the price function" (Kratena et al., 2013, p.90). This implies that regarding prices, FIDELIO is analogous to GPM (as the demand-driven price model). As in GPM, the output price in region r in sector s (denoted by $\mathrm{PQ}(r, s)$) derives the basic prices of domestic products (denoted by $\mathrm{PGD}_{bp}(r, g)$) via the average weights (denoted by $\sum_s \underline{\mathrm{MKSH}^0}(r, g, s)$) (Kratena et al., 2013, Eq.4.91, p.91):

$$\mathrm{PGD}_{bp}(r, g) = \sum_s \underline{\mathrm{MKSH}^0}(r, g, s) \cdot \mathrm{PQ}(r, s) \tag{D6.3}$$

Note that $\mathrm{PGD}_{bp}(r, g)$ is further divided into each of prices. Here, notice that Eq.D6.3 is analogous to GPM (Eq.18) as:

$$\mathbf{p} = (\mathbf{I} - \mathbf{B})^{-1}\mathbf{D}\mathbf{p}_\mathbf{y} = \mathbf{G}\mathbf{D}\mathbf{p}_\mathbf{y} \tag{18}$$

where $\mathrm{PQ(r,s)}$ is analogous to $\mathbf{p}_\mathbf{y}$ or $\mathbf{D}\mathbf{p}_\mathbf{y}$. Thus, FIDELIO can analyze the change in output prices, $\Delta\mathrm{PQ(r,s)}$, in a manner similar to $\Delta(\mathbf{D}\mathbf{p}_\mathbf{y})$ in GPM.

Although FIDELIO is too complex to rewrite the whole model, as an idea, we can consider converting FIDELIO to the supply-driven model for some key points. First of all, FIDELIO considers first that the final demand derives the supply values (as in LQM) and then that the output prices affect various prices (as in GPM). However, this way of thinking is the opposite of this study (Section 2.1). This study considers first that the price of primary inputs decides the output price (as in LPM), and then that the input value affects the output values (as in GQM).

Specifically, LPM (Eq.6) derives the output price ($\mathbf{p}'$) from the price of primary inputs ($\mathbf{p}_\mathrm{v}'\mathbf{C}$).

$$\mathbf{p}' = \mathbf{p}_\mathrm{v}'\mathbf{C}(\mathbf{I} - \mathbf{A})^{-1} = \mathbf{p}_\mathrm{v}'\mathbf{C}\mathbf{L} \tag{6}$$

Thus, analogously, Eq.D6.3 in FIDELIO is rewritten in the opposite direction, where the input price ($\mathrm{PGD_{bp}(r,g)}$) determines the output price ($\mathrm{PQ(r,s)}$), via some function $f_1(\cdot)$.

$$\mathrm{PQ(r,s)} = f_1\left(\mathrm{PGD_{bp}(r,g)}\right) \tag{D6.4}$$

Although this study is based on LPM, the change of the input quantity (Δq_i) is supposed to decide the change in $\mathbf{p}_\mathrm{v}'\mathbf{C}$ (Eq.44):

$$\Delta(\mathbf{p}_\mathrm{v}'\mathbf{C}) = \Delta\mathbf{p}'\hat{\mathbf{c}} = \left[\frac{\Delta q_i}{q_i \varepsilon_i}\right]'\hat{\mathbf{c}} = \left[\frac{\theta_i - 1}{\varepsilon_i}\right]'\hat{\mathbf{c}} \tag{44}$$

Therefore, analogously, the input price in FIDELIO may be affected by the value added via some function $f_2(\cdot)$:

$$\mathrm{PGD_{bp}(r,g)} = f_2\left(\mathrm{VA(r,s)}\right) \tag{D6.5}$$

where $\mathrm{VA(r,s)}$ is the total value added at base price (bp) of sector s in region r. When linking Eq.44 to Eq.6, we derive Eq.45, meaning that the change in the value added (i.e., the supply constraint) affects the output price ($\Delta\tilde{\mathbf{p}}'$).

$$\Delta\tilde{\mathbf{p}}' = \Delta(\mathbf{p}_\mathrm{v}'\mathbf{C})\mathbf{L} = \Delta\mathbf{p}'\hat{\mathbf{c}}\mathbf{L} = \left[\frac{\Delta q_i}{q_i \varepsilon_i}\right]'\hat{\mathbf{c}}\mathbf{L} = \left[\frac{\theta_i - 1}{\varepsilon_i}\right]'\hat{\mathbf{c}}\mathbf{L} \tag{45}$$

Similarly, we can link Eq.D6.5 to Eq.D6.4 via the functions f_1 and f_2.

$$\mathrm{PQ(r,s)} = f_1\left(\mathrm{PGD_{bp}(r,g)}\right) = f_1\{f_2\left(\mathrm{VA(r,s)}\right)\} \tag{D6.6}$$

Eq.D6.6 indicates that the change in value added (i.e., $\Delta\mathrm{VA(r,s)}$) will affect the change in output price (i.e., $\Delta\mathrm{PQ(r,s)}$). Finally, the model of this study presupposes that the output price decides the output quantity, meaning that consumers buy less (more) if the output price is higher (lower) (Eq.46).

$$\Delta\tilde{q}_i = \varepsilon_i \Delta\tilde{p}_i x_i \tag{46}$$

Analogously, as rewriting Eq.D6.1 in FIDELIO, the demand for each user (u) at the base price (bp) (i.e., $\mathrm{GD_{bp}(r,g,u)}$) is decided by the output price ($\mathrm{PQ(r,s)}$) via some function $f_3(\cdot)$:

$$\sum_{\mathrm{u}} \mathrm{GD_{bp}(r,g,u)} = f_3\big(\mathrm{PQ(r,s)}\big) = f_3\big[f_1\{f_2\big(\mathrm{VA(r,s)}\big)\}\big] \tag{D6.7}$$

Note that each of users (u) may decide the demand quantity, depending on their utility functions. In this way, we can examine how the supply constraint (i.e., $\Delta\mathrm{VA(r,s)}$) can affect the demand as in the model of this study.

D.7 Spatial substitution and price multipliers: the FIDELIO model in Kratena and Streicher (2017)

Regarding advanced issues, this part discusses spatial substitution and price multipliers. That is, when a disaster causes a supply shock (i.e., supply constraint), we may wonder which areas will be damaged (i.e., spatial substitution) and how much the economic impact will be (i.e., price multipliers). As background, Kratena and Streicher (2017) recently examined the fiscal policy simulations in the aftermath of the financial crisis (i.e., the stability and magnitude of fiscal policy multipliers) using the FIDELIO model (see Supplementary Information D.6). Covering 67 countries (i.e., EU countries and rest of Europe), the simulation supposes that there is a 1% shock to GDP in Spain (as an EU economy) over a ten-year period (i.e., shocks to public expenditures, capital taxes, and transfer payments). The estimated result shows multipliers are about 1.9 (1.6) for public consumption and 1.2 (0.9) for household taxes or transfers in the case of high (low) liquidity constraints.

Regarding the spatial substitution, Kratena and Streicher (2017) simulate the effect of the shock (1% of GDP) in Spain on all 67 countries. Such an analysis is possible in the model of this study because this study is already an MRIO model (i.e., 47 prefectures in Japan).

Note, however, that FIDELIO takes various prices, whereas this study conducts a domestic model (i.e., Japan). Thus, we here consider taking various prices as in FIDEIO and as in Fig. S4 (Supplementary Information C). Suppose that the price of final output ($\mathbf{p_y}$) consists of the following four prices: prices for

the final outputs of the household ($\mathbf{p_{hou}}$), government ($\mathbf{p_{gov}}$), investments (capital formation; $\mathbf{p_{inv}}$), and net exports ($\mathbf{p_{exp}}$). These prices are expressed in GPM, given the coefficient **B** is fixed.

$$\mathbf{p} = \mathbf{Bp} + \mathbf{Dp_y} = \mathbf{Bp} + \mathbf{D}[\mathbf{p_{hou}}, \mathbf{p_{gov}}, \mathbf{p_{inv}}, \mathbf{p_{exp}}] \Leftrightarrow \mathbf{D}[\mathbf{p_{hou}}, \mathbf{p_{gov}}, \mathbf{p_{inv}}, \mathbf{p_{exp}}] = (\mathbf{I} - \mathbf{B})\mathbf{p} \quad \text{(D7.1)}$$

where the bracket means the vector (i.e., the four factors of prices). In this case, suppose that the model of this study calculates the spillover change in price ($\Delta\tilde{\mathbf{p}}$) in Eq.38. Thus, substituting $\Delta\tilde{\mathbf{p}}$ to Eq.D7.1, the change of the final output price ($\Delta(\mathbf{Dp_y})$) is calculated:

$$\Delta(\mathbf{Dp_y}) = \Delta(\mathbf{D}[\Delta\mathbf{p_{hou}}, \Delta\mathbf{p_{gov}}, \Delta\mathbf{p_{inv}}, \Delta\mathbf{p_{exp}}]) = (\mathbf{I} - \mathbf{B})\Delta\tilde{\mathbf{p}} \quad \text{(D7.2)}$$

Therefore, some rationing scheme can divide $\Delta\mathbf{p_y}$ into each of $\Delta[\mathbf{p_{hou}}, \mathbf{p_{gov}}, \mathbf{p_{inv}}, \mathbf{p_{exp}}]$.

We then can explain the price multiplier in IOA. In LQM and LPM, the Leontief inverse (**L**) causes the round effect (see Eq.D1.1 above). Thus, the Leontief multiplier in each sector i (lm_i as the row vector) is expressed as the column sum of **L** as follows:

$$[lm_i] = \mathbf{i}'\mathbf{L} = \mathbf{i}'(\mathbf{I} - \mathbf{A})^{-1} = \mathbf{i}'(\mathbf{I} + \mathbf{A} + \mathbf{A}^2 + \mathbf{A}^3 + \cdots) \quad \text{(D7.3)}$$

where **i** denotes a summation vector (of one). Similarly, the Ghosh multiplier in each sector i (gm_i as the column vector) is expressed as the column sum of **G** as follows:

$$[gm_i] = \mathbf{Gi} = (\mathbf{I} - \mathbf{B})^{-1}\mathbf{i} = (\mathbf{I} + \mathbf{B} + \mathbf{B}^2 + \mathbf{B}^3 + \cdots)\mathbf{i} \quad \text{(D7.4)}$$

As a price multiplier, FIDELIO (the demand-driven model) should use the Ghosh multiplier (because of GPM; Eq.D7.4). Meanwhile, the model of this study (the supply-driven model) should use the Leontief multiplier (because of LPM; Eq.D7.3).

D.8 The supply constraint in GQM

Some readers also may wonder if the data of this study can be applied to the disaster models developed in previous studies. Because most of the models in the literature have adopted LQM (as the demand-driven model), however, we could not find such models that could be compared directly with this study (i.e., the supply-driven model) at the present moment. Therefore, here we compare the damage of the supply constraint in GQM as follows. Note that GQM itself is considered implausible in the IOA literature (see Section 2.3).

GQM (Eq.10) is expressed as:

$$\mathbf{x}' = \mathbf{v}'(\mathbf{I} - \mathbf{B})^{-1} = \mathbf{v}'\mathbf{G} \Leftrightarrow \mathbf{v}' = \mathbf{x}'(\mathbf{I} - \mathbf{B}) = \mathbf{x}'\mathbf{G}^{-1} \quad \text{(D8.1)}$$

Given that $\mathbf{B}$ is fixed, suppose that a disaster causes the supply constraint to the primary inputs. For example, the survival coefficient (or production capacity) $\mathbf{\Theta} = [\theta_i]$ constrains the primary inputs directly as $\mathbf{v}'\widehat{\mathbf{\Theta}}$ (where $\widehat{\mathbf{\Theta}}$ is the diagonal matrix of $\mathbf{\Theta}$). Replacing $\mathbf{v}'$ with $\mathbf{v}'\mathbf{\Theta}$ in Eq.D8.1:

$$\mathbf{v}'\widehat{\mathbf{\Theta}}(\mathbf{I}-\mathbf{B})^{-1} = \mathbf{x}'(\mathbf{I}-\mathbf{B})\widehat{\mathbf{\Theta}}(\mathbf{I}-\mathbf{B})^{-1} = \mathbf{x}'\mathbf{G}^{-1}\widehat{\mathbf{\Theta}}\mathbf{G} \qquad \text{(D8.2)}$$

Thus, if the primary inputs will be restricted to $\mathbf{v}'\widehat{\mathbf{\Theta}}$, the production ($\mathbf{x}'$) is changed (i.e., most likely decreased) to be $\mathbf{x}'\mathbf{G}^{-1}\widehat{\mathbf{\Theta}}\mathbf{G}$. Notice that $\mathbf{x}'\mathbf{G}^{-1}\widehat{\mathbf{\Theta}}\mathbf{G}$ is different from $\mathbf{x}'\widehat{\mathbf{\Theta}}$ although they may take similar values with each other:

$$\mathbf{x}'\mathbf{G}^{-1}\widehat{\mathbf{\Theta}}\mathbf{G} \neq \mathbf{x}'\widehat{\mathbf{\Theta}} \qquad \text{(D8.3)}$$

Eq.D8.3 means that in GQM, the supply constraint is likely to decrease the production directly via $\mathbf{G}^{-1}\widehat{\mathbf{\Theta}}\mathbf{G}$. Compared to GQM, the model of this study does not always decrease (or even increases) the production (Section 3.4). As we can confirm, the loss of PS (ΔPS; which is the difference of half-production before and after the disaster) takes even negative values (Tables V and VI).

Supplementary Information E: IIP and disaster damage

IIP covers production (in all prefectures), shipments, and inventories, and this study uses the production IIP because it has abundant production data as an actual index. Because of the real index, however, IIP has a drawback in that it is affected not only by the direct effect of disaster but also by the indirect effect among sectors, which may be somewhat mitigated by the inventories.

The direct damage lowers IIP due to labor and capital damage. The indirect damage may further lower IIP due to the balance (i.e., bottleneck) of supply and demand in the supply chain. Note, however, that this indirect damage can be alleviated to some extent by the amount of inventory, covering shipping capacity. If there are enough amounts of inventories, because shipping capacity can be covered by inventory to some extent, production damage does not spill over to the whole supply chain.

For example, suppose there are five sectors in a product supply chain: raw materials, components, and manufacture as the manufacturing sector, retail as the service sector, and the final consumers (Supplementary Information Fig. S2). The first (raw material) to fourth (retail) sectors are on the supply side, and are involved in conducting production, shipments, and inventory processes (which are all covered by IIP). The maximum volume of shipments depends on production and inventory, and shipments are realized based on the balance between supply and demand. Meanwhile, the second to last (consumer) sectors are on the demand side, purchasing a product from each of the preceding suppliers.

Suppose a disaster stops only the production of raw material. The decrease in production will affect shipping capacity, potentially changing demand (e.g., volume and price) as the intermediate input of components. Because of the shortage of the intermediate input, the supply of components may be affected, potentially changing demand for the components in the manufacturing sector (etc.). Note, however, that inventory is important for such indirect damage. If there is no inventory, production damage directly affects shipping capacity, and therefore largely affects supply and demand throughout the supply chain. Meanwhile, if there are enough inventories, because shipping capacity can be covered by inventory to some extent, production damage does not spill over to the entire supply chain.

Supplementary Information F: Indirect damages in H95Jan (Toyoda & Kouchi, 1997) and GEJE (Hayashi, 2012)

In disaster studies, the loss of SS is not widespread for estimating damage. Instead, two popular damages are direct damage (e.g., damage to capital stock) and indirect damage (e.g., flow damage due to the spillover effect). This study supposes that the loss of SS is similar to indirect damage because it does not consider the damage to capital stock and so on. In other words, the indirect damage in the previous studies is divided into those of buyers (in the downstream sector) and sellers (in the upstream sector), which are similar to the losses of CS and PS, respectively. This study supposes that the reason SS is not popular is that CS is difficult to estimate (although PS is easy). CS is calculated from the difference between reservation price (i.e., willingness-to-pay price) and transaction price. However, the reservation price is usually difficult to estimate. Meanwhile, PS is calculated from the difference between the transaction price and cost, which are easy to determine (with assumptions).

Two previous studies that estimated damage due to H95Jan (Toyoda & Kouchi, 1997) and GEJE (Hayashi, 2012) are introduced for comparative purposes (see Supplementary Information Table S10). Shortly after H95Jan (April 5, 1995), the Hyogo prefectural government (and National Land Agency, Japan) estimated that the direct damage (to capital stock) caused by the disaster totaled 9,926.8B JPY. Toyoda and Kouchi (1997) aimed to update this estimate using a questionnaire survey, which asked sample firms about direct and indirect damage amounts. The survey period spanned January 29 to February 15, 1996, and valid responses were elicited from 1,246 representatives of firms under the auspices of the Kobe Chamber of Commerce and Industry (1,086 firms for direct damage and 810 firms for indirect damage). Toyoda and Kouchi (1997) calculated disaster coefficients from the survey data and estimated the damage in ten cities and ten towns in Hyogo (which were severely damaged). The results suggest that direct damage totaled 5,930B JPY and 1,510B JPY for the industrial sectors, whereas indirect damage for one year was 7,230B JPY in total and 1,203B JPY for the industrial sectors. Based on their results, Toyoda and Kouchi (1997) argued that total direct damage should be 13,268.2B JPY.

Meanwhile, Hayashi (2012) estimated the direct and indirect damage caused by GEJE. Immediately after GEJE, the national government estimated the direct damage to be approximately 16,900B JPY (or 3.5% of GDP). Hayashi (2012) considered the additional cost of the damage and argued that the direct damage (excluding the indirect damage caused by the nuclear accident) should be valued higher, at

approximately 30T JPY (6% of GDP). Also, the indirect damage was estimated to be approximately 10T JPY in Fukushima alone and approximately 100T JPY with respect to annual gross regional product over the decade. Hayashi (2012) argued that, overall, the damage caused by GEJE was three to four times higher than that caused by H95Jan.

Figs.

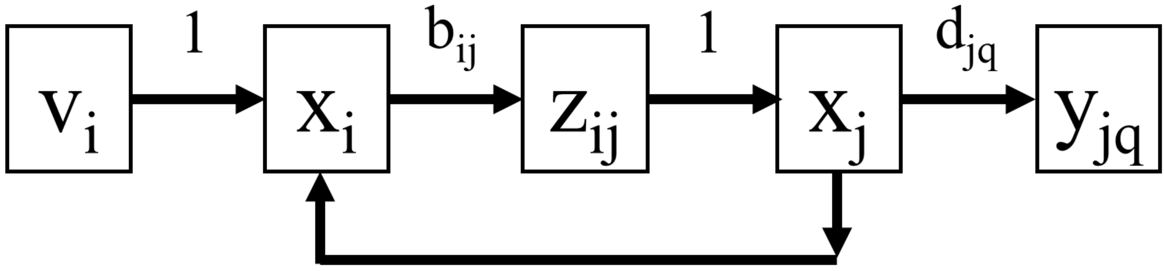

Fig. S1. Causal chain representation of the Ghoshian model (Oosterhaven, 1988, p.206, Fig. 1)

Notes: Source: Oosterhaven (1988). The original "d_{iq}" was probably a typo (i.e., "i"), and therefore, this study changes it to "d_{jq}".

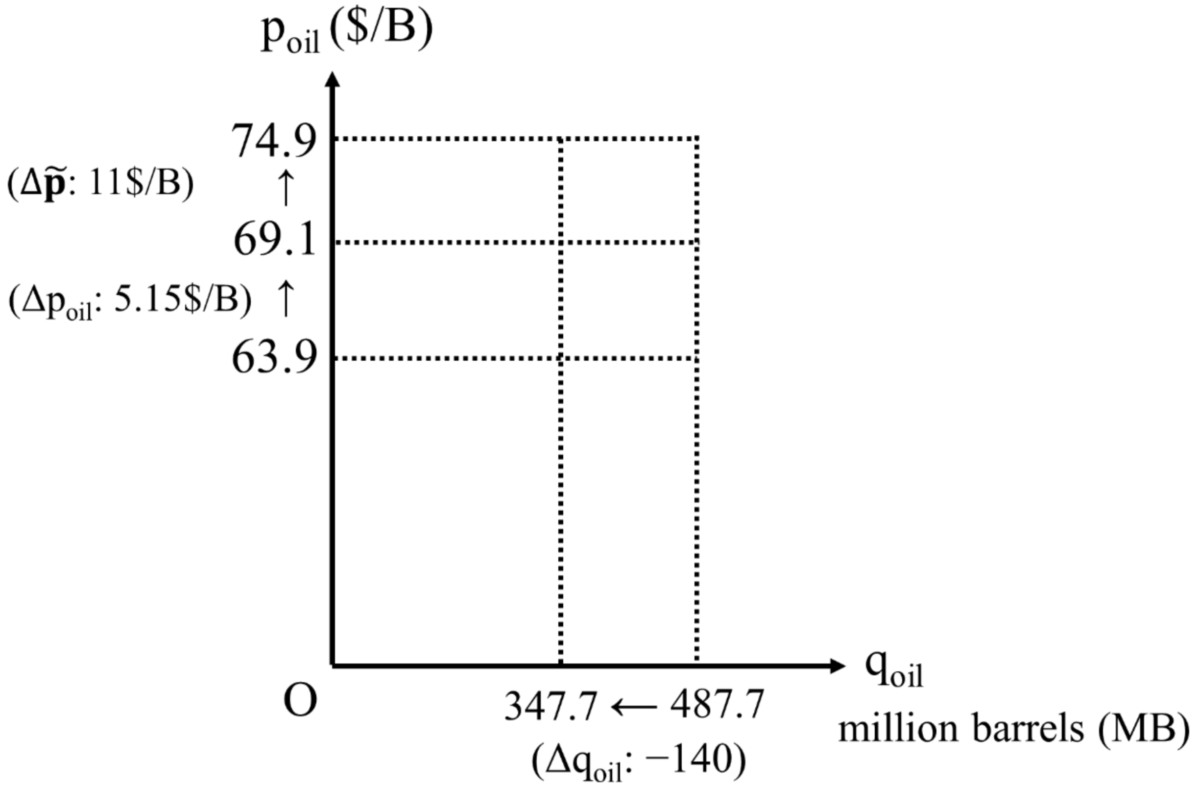

Fig. S2. Economic loss estimation in Park (2009)

Notes: The quantity (q_{oil}) was originally expected to be 487.7MB, and the quantity change (Δq_{oil}) was −140MB for the four months. The oil price before the hurricanes would be 63.9$/B. Park (2009) calculated that the direct loss (Δp_{oil}) was 5.1534 $/B (to be 69.1$/B) and that the total economic loss ($\Delta\tilde{\mathbf{p}}$) is 10.9780 $/B (to be 74.9$/B).

		Intermediate demand ($\mathbf{Z}$: I-by-I matrix) I sectors: 1, ..., I					Final demand ($\mathbf{Y}$: I-by-M matrix) ($\mathbf{y}=\mathbf{Yi}$: I-vector) M categories: 1, ..., M					Production ($\mathbf{x}$: I-vector)
		1	⋯	j	⋯	I	1	⋯	m	⋯	M	
Intermediate input ($\mathbf{Z}$: I-by-I matrix) I sectors: 1, ..., I	1	z_{11}	⋯	z_{1j}	⋯	z_{1I}	y_{11}	⋯	y_{1m}	⋯	y_{1M}	x_1
	⋮	⋮	⋱		⋰	⋮	⋮	⋱		⋰	⋮	⋮
	i	z_{i1}		z_{ij}		z_{iI}	y_{i1}		y_{im}		y_{iM}	x_i
	⋮	⋮	⋰		⋱	⋮	⋮	⋰		⋱	⋮	⋮
	I	z_{I1}	⋯	z_{Ij}	⋯	z_{II}	y_{I1}	⋯	y_{Im}	⋯	y_{IM}	x_I
Primary input (value added) ($\mathbf{V}$: N-by-I matrix) ($\mathbf{v'}=\mathbf{i'V}$: I-vector) N categories: 1, ..., N	1	v_{11}	⋯	v_{1j}	⋯	v_{1I}						
	⋮	⋮	⋱		⋰	⋮						
	n	v_{n1}		v_{nj}		v_{nI}						
	⋮	⋮	⋰		⋱	⋮						
	N	v_{N1}	⋯	v_{Nj}	⋯	v_{NI}						
Production ($\mathbf{x}$: I-vector)		x_1	⋯	x_j	⋯	x_I						

Fig. S3. IO table in this study

Note: To facilitate understanding by comparison, the IO model of this study follows the explanation in Oosterhaven (1996).

		Intermediate demand ($\mathbf{Z}$: I-by-I matrix) I sectors: 1, ..., I			Final demand ($\mathbf{Y}$: I-by-4 matrix) Four categories				Production ($\mathbf{x}$: I-vector)
		1	⋯	I	Household (1)	Government (2)	Investment (capital formation) (3)	Net export (4)	
Intermediate input ($\mathbf{Z}$: I-by-I matrix) I sectors: 1, ..., I	1	z_{11}	⋯	z_{1I}	y_{11} (=hou_1)	y_{12} (=gov_1)	y_{13} (=inv_1)	y_{14} (=exp_1)	x_1
	⋮	⋮	⋱	⋮	⋮	⋮	⋮	⋮	⋮
	I	z_{I1}	⋯	z_{II}	y_{I1} (=hou_I)	y_{I2} (=gov_I)	y_{I3} (=inv_I)	y_{I4} (=exp_I)	x_I
Primary input (value added) ($\mathbf{V}$: 4-by-I matrix) Four categories	Labor (1)	v_{11} (=lab_1)	⋯	v_{1I} (=lab_I)	**0**				**lab** =$\sum lab_j$
	Capital (2)	v_{21} (=cap_1)	⋯	v_{2I} (=cap_I)					**cap** =$\sum cap_j$
	Investment (depreciation) (3)	v_{31} (=dep_1)	⋯	v_{3I} (=dep_I)					**dep** =$\sum dep_j$
	Tax (4)	v_{41} (=tax_1)	⋯	v_{4I} (=tax_I)					**tax** =$\sum tax_j$
Production ($\mathbf{x}$: I-vector)		x_1	⋯	x_I	**hou** =$\sum hou_i$	**gov** =$\sum gov_i$	**inv** =$\sum inv_i$	**exp** =$\sum exp_i$	

Fig. S4. Example of an IO table

Notes: This figure is an example of an IO table. Regarding the demand, intermediate demand has I sectors, and final demand has four categories (household, government, investment [as capital formation], and net export [as an external sector]). Regarding the input, intermediate input has I sectors, and primary input (value added) has four categories (labor, capital, investment [as capital depreciation], and tax). Regarding the balance of supply and demand, intermediate inputs and demand are balanced (I-by-I square matrix). Meanwhile, primary input and final demand are not balanced (hence, total values do not match).

		Activity (I sectors)			Factor		Household and institutions			External	Total
		1	⋯	I	Labor	Capital	Household	Government	Investment (capital formation)	Net export	
Activity (I sectors)	1	z_{11}	⋯	z_{1j}	0	0	y_{11} (=hou_1)	y_{12} (=gov_1)	y_{13} (=inv_1)	y_{14} (=exp_1)	x_1
	⋮	⋮	⋱	⋮	⋮	⋮	⋮	⋮	⋮	⋮	⋮
	I	z_{i1}	⋯	z_{ij}	0	0	y_{i1} (=hou_i)	y_{i2} (=gov_i)	y_{i3} (=inv_i)	y_{i4} (=exp_i)	x_i
Factor	Labor (lab)	v_{11} (=lab_1)	⋯	v_{1j} (=lab_j)	0	0	0	0	0	exp_{lab}	**lab**
	Capital (cap)	v_{21} (=cap_1)	⋯	v_{2j} (=cap_j)	0	0	0	0	0	exp_{cap}	**cap**
Household and institutions	Household	0	⋯	0	lab_{hou}	cap_{hou}	0	gov_{hou}	0	exp_{hou}	**hou**
	Government (= Tax)	v_{41} (=tax_1)	⋯	v_{4j} (=tax_1)	0	0	tax_{hou}	0	0	exp_{gov}	**gov (=tax)**
	Investment (depreciation)	v_{31} (=dep_1)	⋯	v_{3j} (=dep_j)	0	0	dep_{hou}	dep_{gov}	0	exp_{dep}	**inv (=dep)**
External	Foreign countries (= Net export)	0	⋯	0	lab_{exp}	cap_{exp}	hou_{exp}	gov_{exp}	inv_{exp}	0	**exp**
Total		x_1	⋯	$x_{j\,(=i)}$	**lab**	**cap**	**hou**	**gov (=tax)**	**inv (=dep)**	**exp**	

Fig. S5. Example of SAM

Notes: This figure is an example of SAM extended from Fig. S4. The demand (column) consists of activities (intermediate demand: I sectors), factor (labor and capital), household, government, investment (capital formation), and net export. Similarly, input (row) consists of activities (intermediate inputs: I sectors), factor (labor and capital), household, government (as tax revenue), investment (as capital depreciation), and foreign countries (as an external factor). In SAM, supply and demand are fully balanced not only in the activity sectors but also in the other sectors (hence, a square matrix).

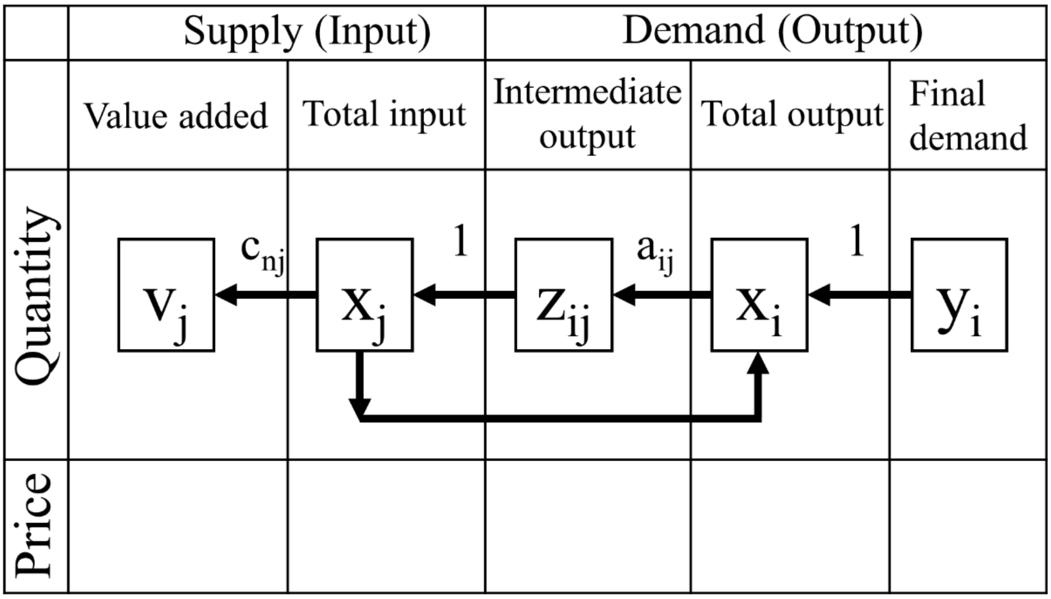

Fig. S6. The round effect of LQM

Notes: See Eq.D1.1. The change in final demand (y) causes the round effect, which affects the amount of the total input (x) and the value added (v).

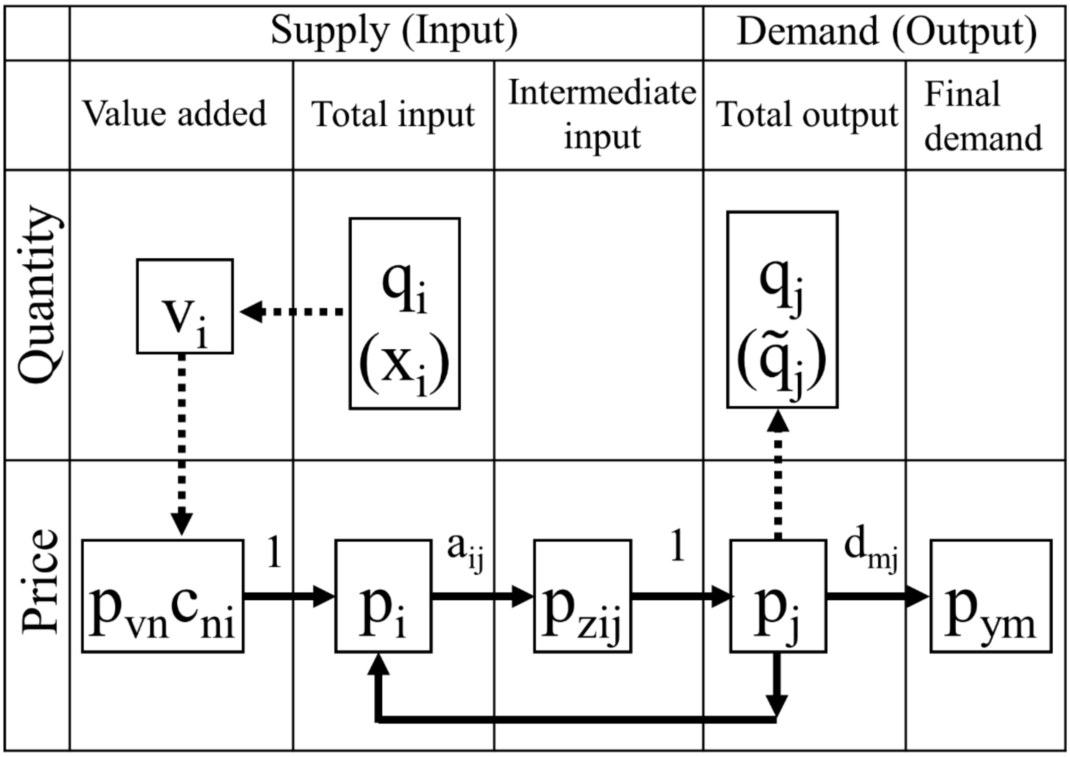

Fig. S7. The round effect of this study based on LPM

Notes: See Eq.D1.2. The quantity change (q or x) is converted to the price change for value added (p_v times c) (i.e., the dotted arrows), which causes the round effect on the prices for total outputs (p) and final demand (p_y times d) (i.e., the straight arrows). Finally, the output price (p) decides the total output quantity (q) (i.e., a dotted arrow).

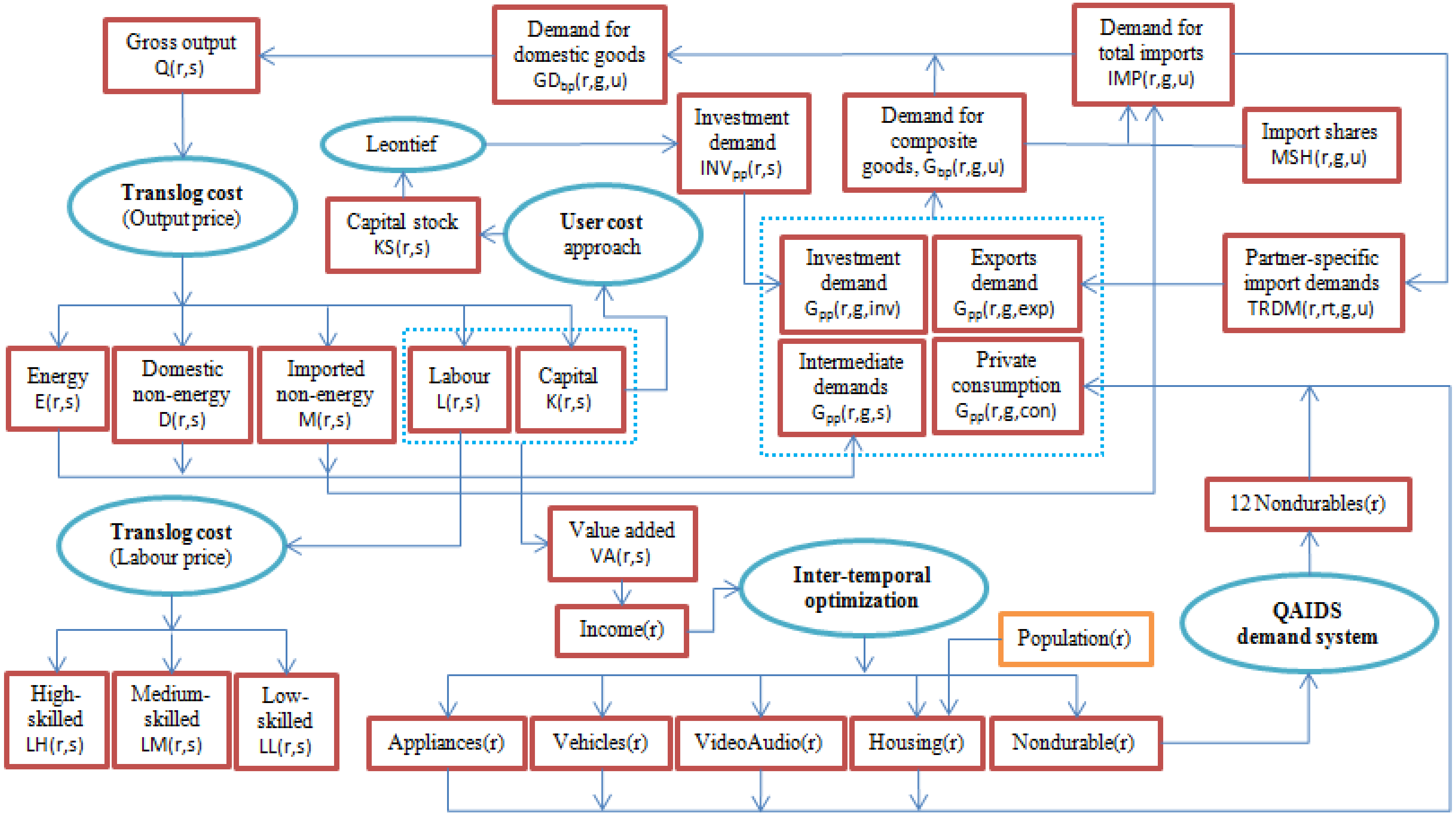

Fig. S8. Overview of the main economic flows in FIDELIO (Kratena et al., 2013, Fig. 1.1, p.5)

Notes: Source: Kratena et al. (2013, Fig. 1.1, p.5). "The variables included within red rectangles are endogenous variables. The main functional forms and approaches used for the derivation of various parts of the model are mentioned within the blue oval shapes."

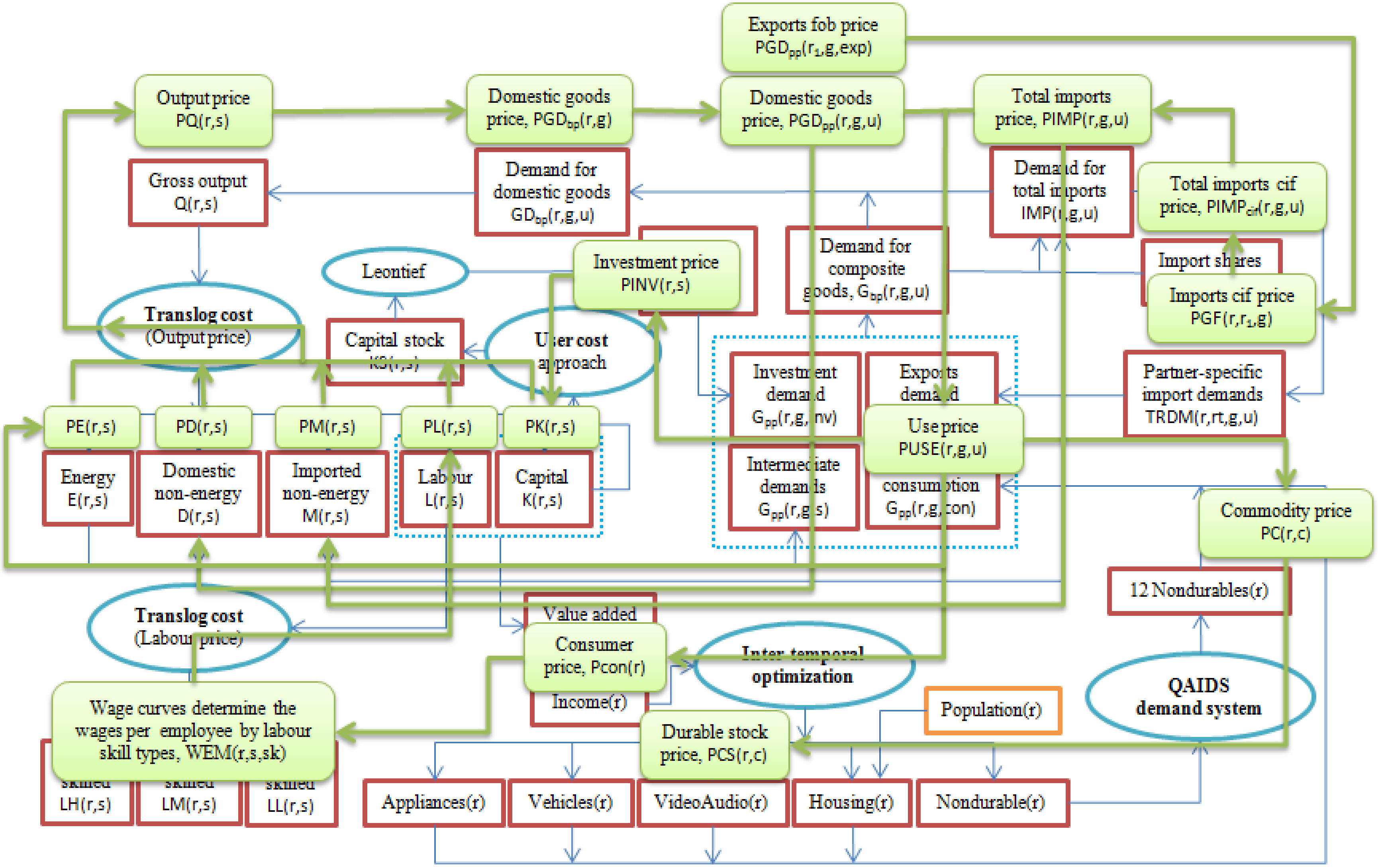

Fig. S9. Overview of selected prices in FIDELIO (Kratena et al., 2013, Fig. 1.2, p.10)

Notes: Source: Kratena et al. (2013, Fig. 1.2, p.10). "Wherever possible, prices (defined within the green rectangles) are positioned/juxtaposed with the transactions which they refer to."

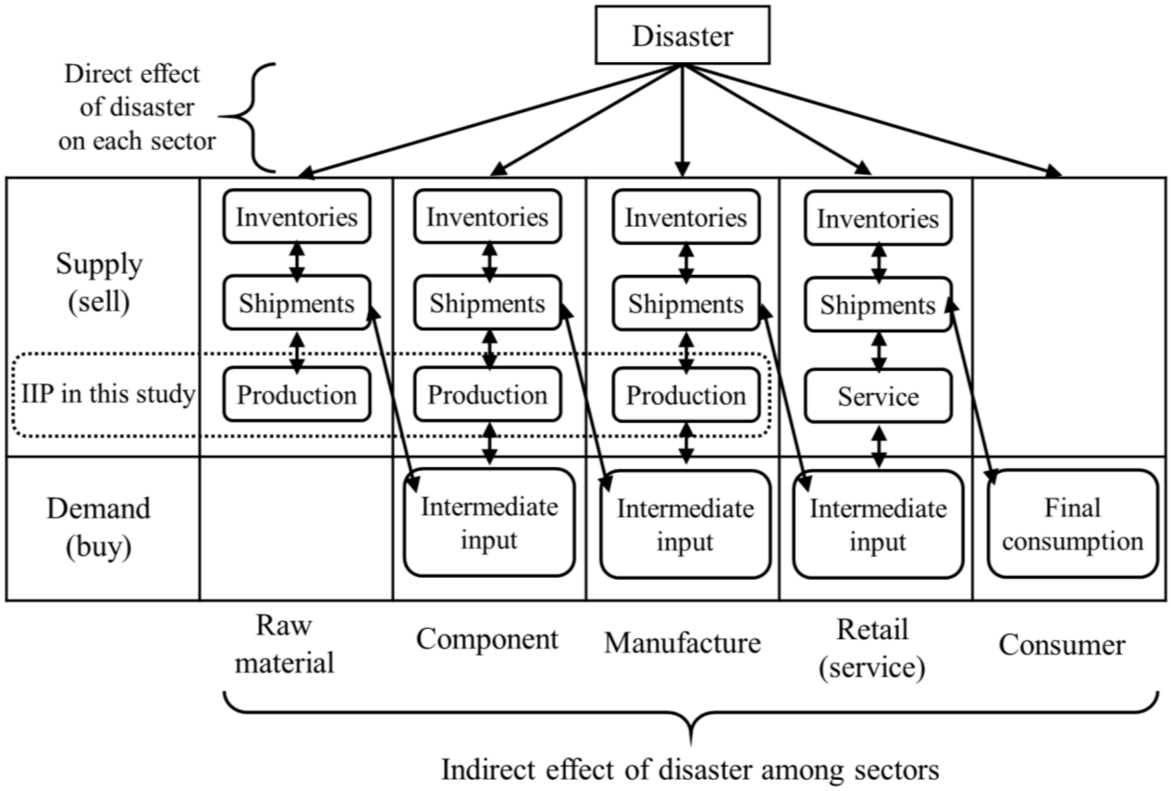

Fig. S10. Direct damage of a disaster and indirect damage among sectors in a supply chain of five sectors (as an example)

Notes: This figure shows the direct damage caused by a disaster and indirect damage among sectors in an example of a supply chain of five sectors: raw materials, components, and manufacture as the manufacturing sectors, retail as the service sector, and final consumers. The supply consists of production (or service), shipments (to the next sectors), and inventories, and demand means purchasing from the previous sectors. IIP consists of production, shipments, and inventories, and this study uses the production IIP. Shipping capacity affects demand (market price and quantity) and depends on production and inventories. If there is no inventory, production damage directly affects shipping capacity. Meanwhile, if there are enough inventories, production damage will be mitigated to some extent by covering shipping capacity.

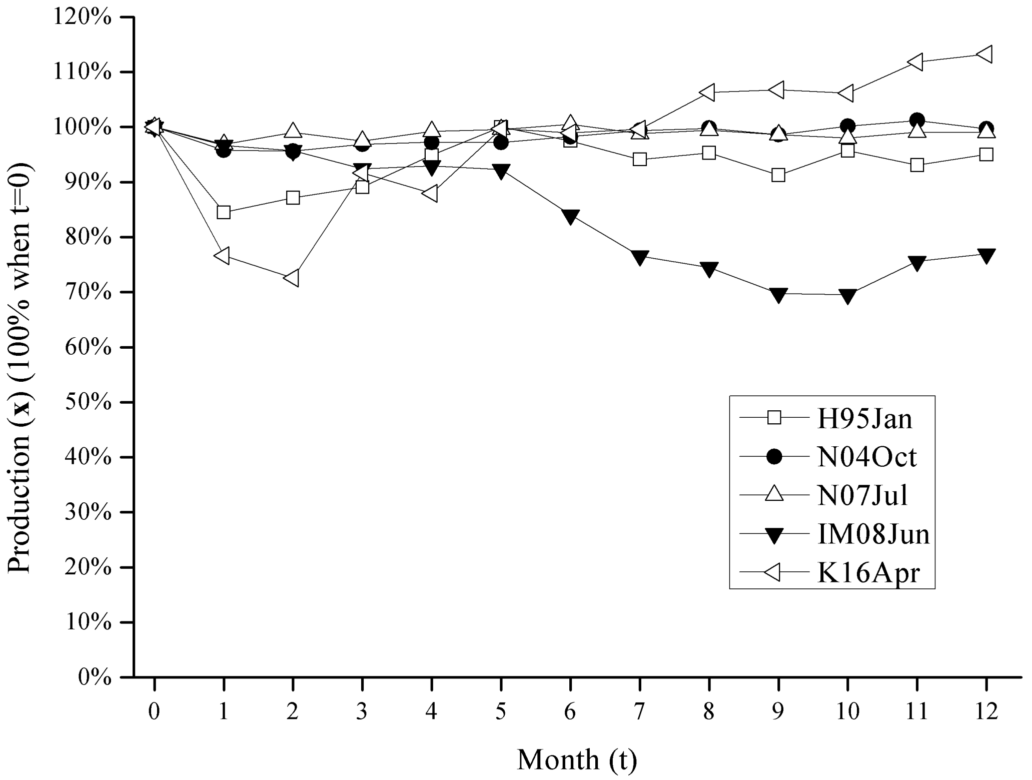

Fig. S11. Production capacity of H95Jan, N04Oct, N07Jul, IM08Jun, and K16Apr
Note: See Table III.

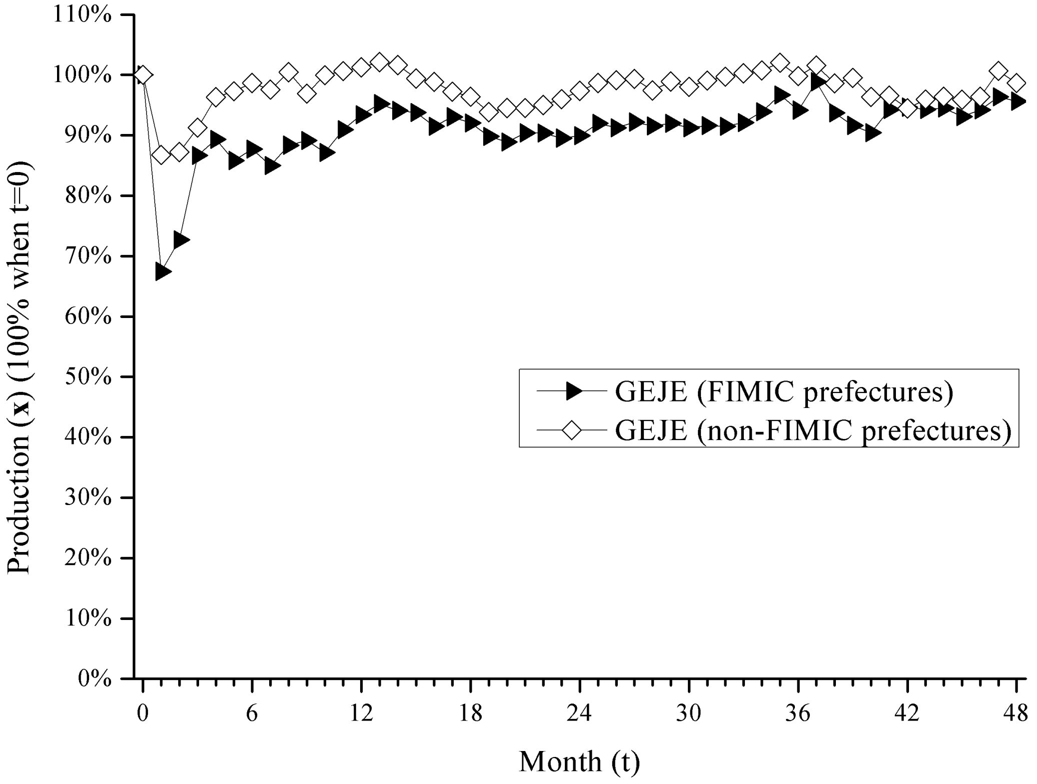

Fig. S12. Production capacity of GEJE
Note: See Table III.

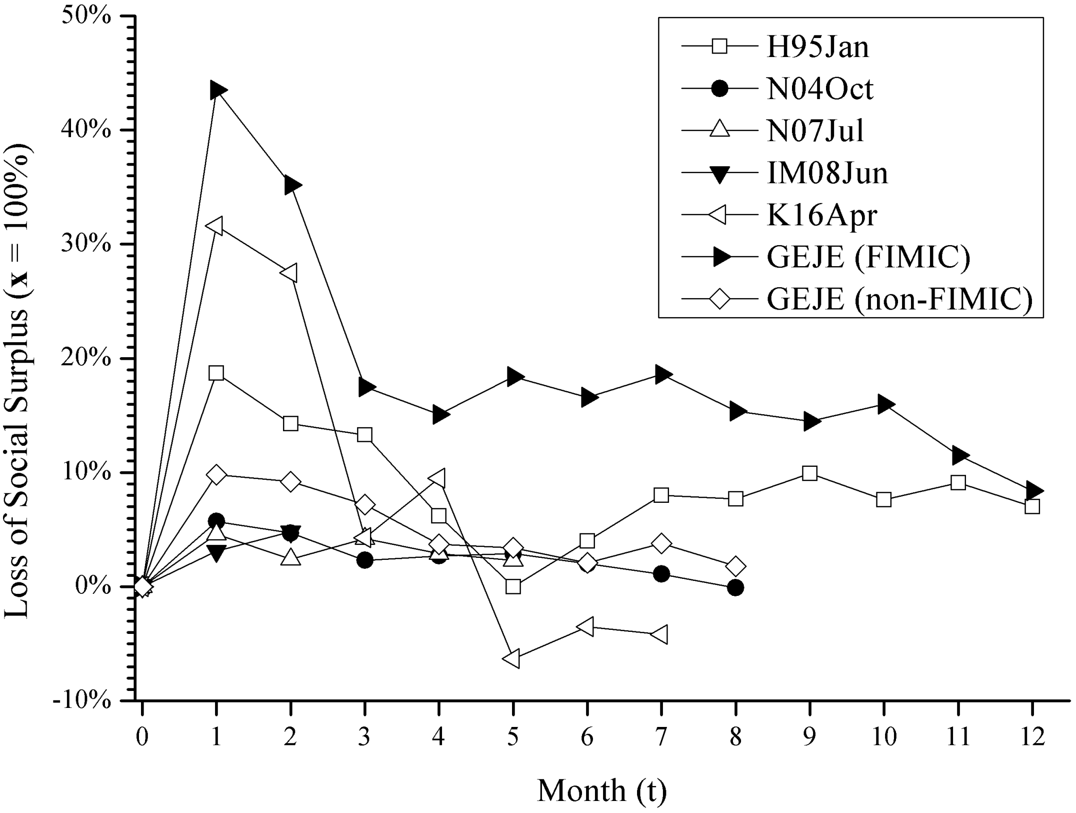

Fig. S13. Loss of social surplus (initial production is 100%)
Note: See Table V.

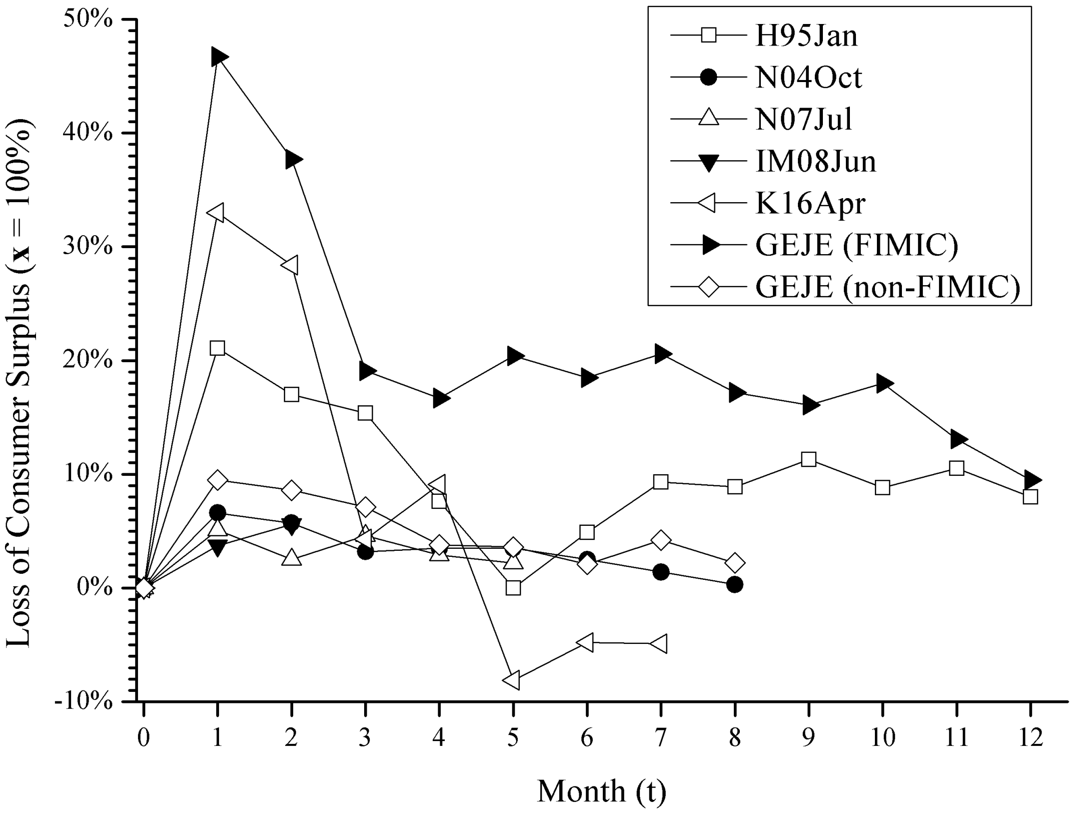

Fig. S14. Loss of consumer surplus (initial production is 100%)
Note: See Table V.

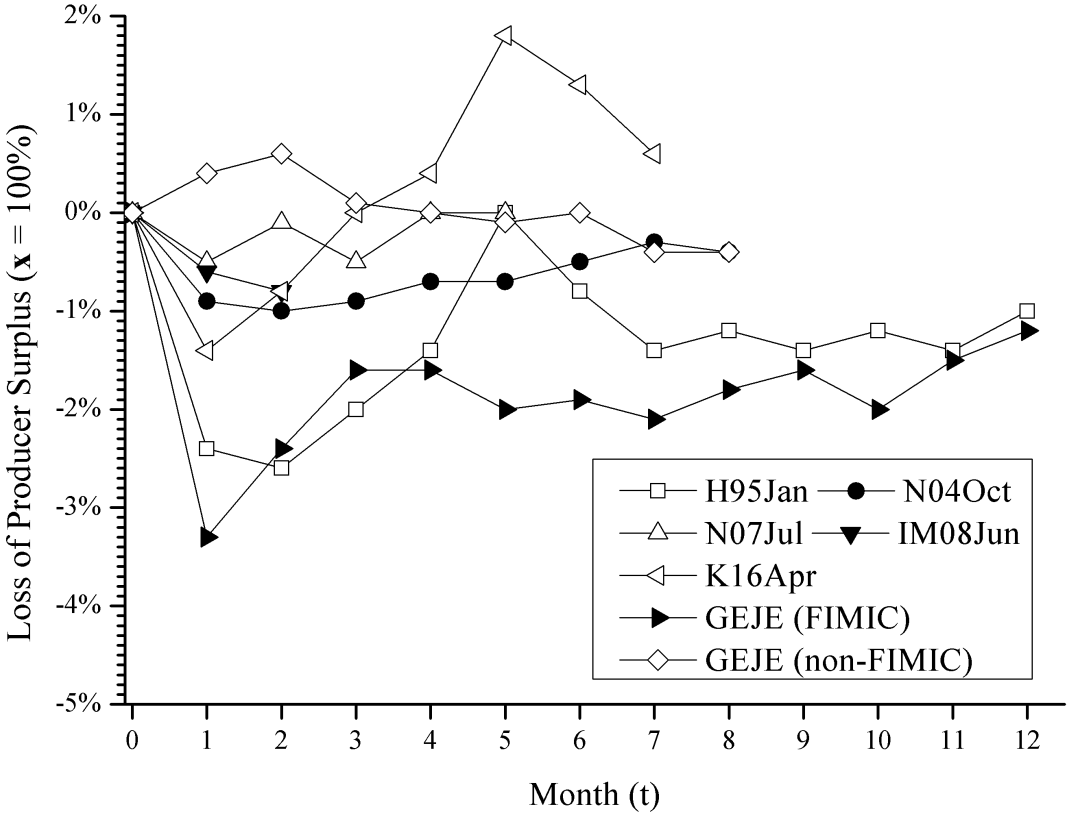

Fig. S15. Loss of producer surplus (initial production is 100%)

Note: See Table V.

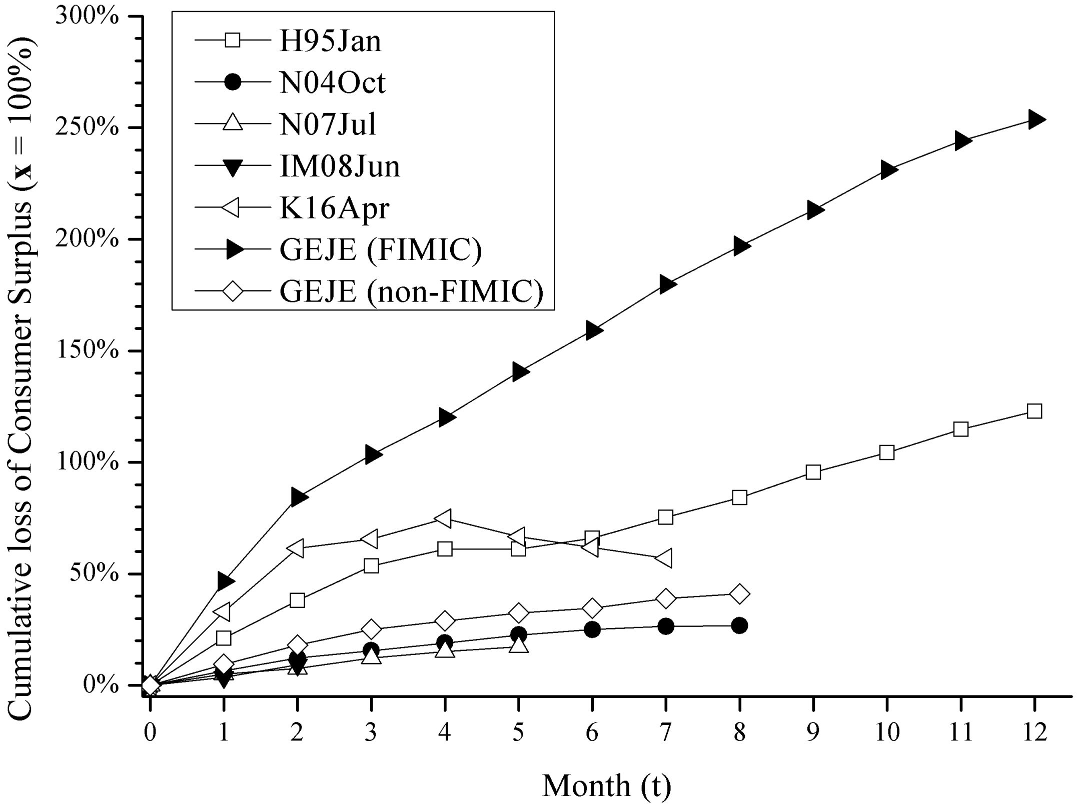

Fig. S16. Cumulative loss of consumer surplus (initial production is 100%)
Note: See Table V.

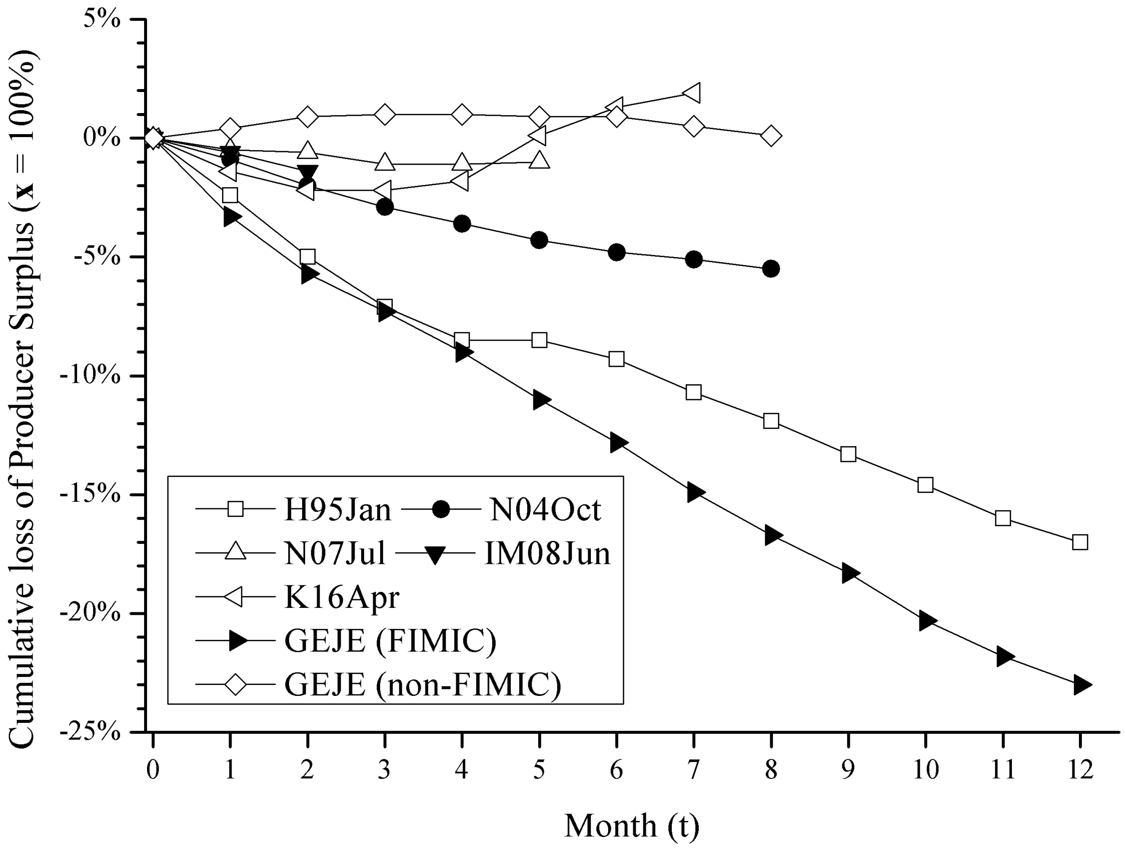

Fig. S17. Cumulative loss of producer surplus (initial production is 100%)
Note: See Table V.

Tables

Table S1. Summary of Data Necessary to Estimate Total Output Vectors (Park, 2009, Table 3, p.22)

Consumption Sector	Oil-Refinery Product Type	q^{oil}	p^{oil}	$\bar{\varepsilon}_p^{oil}$	π^{oil}	Δq^{oil}	W	Δp^{oil}	$W\Delta p^{oil}$
Transportation	Finished Motor Gasoline	263272550	69.528	−0.0334	−0.000008	−37750	0.2696	0.298	0.0804
	Kerosene-Type Jet Fuel	48463714	65.382	−0.0017	−0.000792	−23989	0.1713	18.993	3.2543
	Distillate Fuel Oil	89976381	67.920	−0.0058	−0.000129	−42582	0.3041	5.504	1.6740
	Residual Fuel Oil	6732933	39.336	−0.0058	−0.001000	−4008	0.0286	4.009	0.1148
Residential	Distillate Fuel Oil	12403498	67.920	−0.0997	−0.000055	−5870	0.0419	0.322	0.0135
Commercial	Distillate Fuel Oil	7037068	67.920	−0.4135	−0.000023	−3330	0.0238	0.078	0.0018
	Propane	33689621	41.412	−0.4135	−0.000003	−8493	0.0607	0.025	0.0015
	Residual Fuel Oil	1331172	39.336	−0.4135	−0.000071	−792	0.0057	0.057	0.0003
Industrial	Distillate Fuel Oil	13083732	67.920	−0.2018	−0.000026	−6192	0.0442	0.159	0.0070
	Residual Fuel Oil	11764067	39.336	−0.2018	−0.000017	−7002	0.0500	0.116	0.0058
Total		487754736				−140008	1.0000		5.1534
Unit		Barrel (B)	$/B			1000B		$/B	$/B

Notes: Source: Park (2009, Table 3, p.22). "1) The classifications between consumption sector and oil-refinery product type are available at http://www.eia.doe.gov/emeu/states/sep_use/notes/use_petrol.pdf and 2003 percentages of 'Adjusted Sales of Fuel Oil by End Use' from http://tonto.eia.doe.gov/dnav/pet/pet_cons_top.asp were used to distribute 'Distillate' and 'Residual' Fuel Oil to the consumption sectors. 2) $W_c = \Delta q_c^{oil} / \sum_c \Delta q_c^{oil}$, where subscript c denotes Consumption Sector."

Table S2. Total Impacts via Price-sensitive Supply-driven USIO Model (Park, 2009, Table 4, p.23)

USC Sector	Total Price Increase	Proportion	Total Impact ($M.)
USC1	0.0092	0.084%	−1.29
USC2	0.0105	0.096%	−1.47
USC3	0.0087	0.080%	−1.22
USC4	0.0089	0.081%	−1.24
USC5	0.0077	0.070%	−1.07
USC6	0.0780	0.710%	−10.92
USC7	0.0000	0.000%	−0.01
USC8	0.6115	5.570%	−85.61
USC9	0.1724	1.570%	−24.13
USC10	7.1545	65.171%	−1001.69
USC11	0.2088	1.902%	−29.23
USC12	0.0021	0.019%	−0.29
USC13	0.2882	2.625%	−40.34
USC14	0.0766	0.697%	−10.72
USC15	0.0735	0.670%	−10.29
USC16	0.0421	0.384%	−5.90
USC17	0.0605	0.551%	−8.47
USC18	0.0600	0.546%	−8.40
USC19	0.0129	0.118%	−1.81
USC20	0.0576	0.524%	−8.06
USC21	0.0905	0.825%	−12.67
USC22	0.0895	0.815%	−12.53
USC23	0.0717	0.653%	−10.04
USC24	0.0221	0.201%	−3.09
USC25	0.0481	0.438%	−6.73
USC26	0.0085	0.077%	−1.19
USC27	0.0108	0.099%	−1.51
USC28	0.0115	0.105%	−1.61
USC29	0.0119	0.109%	−1.67
USC30	0.1662	1.514%	−23.26
USC31	0.0088	0.080%	−1.23
USC32	0.1339	1.220%	−18.75
USC33	0.1719	1.566%	−24.06
USC34	0.1064	0.969%	−14.89
USC35	0.0142	0.129%	−1.98
USC36	0.0464	0.422%	−6.49
USC37	0.0535	0.487%	−7.48
USC38	0.1555	1.416%	−21.77
USC39	0.0921	0.839%	−12.89
USC40	0.1819	1.657%	−25.46
USC41	0.0914	0.832%	−12.79
USC42	0.0171	0.156%	−2.39
USC43	0.0032	0.029%	−0.45
USC44	0.0711	0.648%	−9.95
USC45	0.0239	0.218%	−3.34
USC46	0.0082	0.075%	−1.15
USC47	0.3244	2.955%	−45.42
TOTAL	10.9780	100.000%	−1537.01

Note: Source: Park (2009, Table 4, p.23). "Price increases refer to the units in which each sector's outputs are denominated."

Table S3. Comparison between IOA, this study, and CGE

Items	IOA	This study and Park (2007)	CGE
Price and quantity	· Price (model) and quantity (model) are independent with each other	· Supply quantity is exogenous (i.e., supply constraint)<br>· Price is endogenous by price elasticity of demand via supply constraint	· Price and quantity are elastic with each other<br>· Determined simultaneously by utility function to be theoretically consistent
Endogenous sectors (intermediate demand and input)	· Supply and demand are balanced<br>· Based on technical coefficients	(same as IOA)	· Supply and demand are balanced as in IOA<br>· Profit maximization is modelled
Labor and capital (primary inputs of value added)	· Exogenous<br>· Supply and demand may be unbalanced<br>· Free to use	(same as IOA)	· Endogenous<br>· Supply and demand are balanced by corporate utility function (i.e., profit maximization)
Other value added (e.g., investment depreciation)	(ditto)	(same as IOA)	· Exogenous or endogenous, depending on the model setting
Household (in final demand)	(ditto)	(same as IOA)	· Supply and demand are balanced endogenously by utility function (utility maximization)
Government and others (e.g., investment) (in final demand)	(ditto)	(same as IOA)	Exogenous or endogenous, depending on the model setting
Export (or import) in final demand (or external item)	(ditto)	(same as IOA)	(ditto)
Required data	IO table	· IO table<br>· Price elasticity of demand<br>· Supply constraint	· SAM (usually made from IO table)<br>· Other values (if needed) such as price elasticity of demand
Scalability	· Yes<br>· IOA does not matter how many sectors are included<br>· Calculated by spreadsheet (Excel)	(same as IOA)	· No<br>· More sectors, more computational burden<br>· Computational problem is highly non-linear, requiring nonlinear solver (e.g., GAMS)

Table S4. Industry identification numbers

IO id	Industry Name (IO)	IIP id
1	Agriculture, forestry and fishery	—
2	Metallic ores	26
3	Non-metallic ores	26
4	Coal mining, crude petroleum and natural gas	26
5	Food and Tobacco	18
6	Beverage	18
7	Textile products	17
8	Wearing apparel and other textile products	17
9	Timber and wooden products	24
10	Furniture and fixtures	22
11	Pulp, paper, paperboard, building paper	16
12	Paper products	16
13	Publishing, printing	23
14	Chemical fertilizer	13
15	Basic inorganic chemical products	13
16	Basic organic chemical products	13
17	Organic chemical products	13
18	Synthetic resins	15
19	Synthetic fibers	15
20	Final chemical products	13
21	Medicaments	13
22	Petroleum refinery products	13
23	Coal products	13
24	Plastic products	15
25	Rubber products	20
26	Glass and glass products	12
27	Cement and cement products	12
28	Pottery, china and earthenware	12
29	Other ceramic, stone and clay products	12
30	Pig iron and crude steel	3
31	steel products	3
32	Cast and forged steel products	3
33	Other iron or steel products	3
34	Non-ferrous metals	4
35	Non-ferrous metal products	4
36	Metal products for construction and architecture	5
37	Other metal products	5
38	General industrial machinery	6
39	Special industrial machinery	6
40	Other general machines	6
41	Machinery for office and service industry	6
42	Industrial electric equipment	7
43	Applied electrical equipment and electrical measuring instruments	7
44	Other electric equipment	7
45	Household electric and electric applications	7
46	Communication equipment	8
47	Electric computing equipment and accessory equipment	8
48	Semiconductor devices and integrated circuits	9
49	Other electrical equipment	9
50	Passenger motor cars	10
51	Other cars	10
52	Motor vehicle parts and accessories	10
53	Other transportation equipment	10
54	Precision instruments	10

55	Miscellaneous manufacturing products	25
56	Reuse and recycling	—
57	Building construction and repair of construction	—
58	Public construction	—
59	Other civil engineering and construction	—
60	Electricity	—
61	Gas supply and heat supply	—
62	Water supply and waste management services	—
63	Commerce	—
64	Financial and insurance	—
65	Real estate agencies and rental services	—
66	House rent	—
67	Transport	—
68	Communication	—
69	Broadcasting	—
70	Information services	—
71	Internet-based services	—
72	Image information production and distribution industry	—
73	Public administration	—
74	Education and Research	—
75	Medical service, health and social security and nursing care	—
76	Advertising and survey	—
77	Goods rental and leasing services	—
78	Other business services	—
79	Personal services	—
80	Activities not elsewhere classified	—

Notes: This table shows 80 industries for the IOA. IIP id denotes industry codes of IIP, which are used for calculating production capacity (survival coefficients). For the 26 non-mining and manufacturing industry sectors (ID 1 and 56 to 80), there are no IIP ids; thus, these sectors are essentially excluded herein.

Table S5. Industries of IIP for substitution numbers

IIP id	IIP Industry name	Substitute IIP id if there is missing value
1	Mining and manufacturing (Total)	2
2	Manufacturing (Total)	1
3	Iron and steel	2
4	Non-ferrous metals	2
5	Fabricated metals	2
6	General machinery	2
7	Electrical machinery	2
8	Information and communication electronics equipment	2
9	Electronic parts and devices	2
10	Transport equipment	2
11	Precision instruments	2
12	Ceramics, stone and clay products	2
13	Chemicals	2
14	Petroleum and coal products	2
15	Plastic products	2
16	Pulp, paper and paper products	2
17	Textiles	2
18	Foods and tobacco	2
19	Other (Total)	2
20	Rubber products	19
21	Leather products	19
22	Furniture	19
23	Printing	19
24	Wood and wood products	19
25	Other products	19
26	Mining (Total)	1

Notes: This table shows the industry identification numbers of IIP (IIP id). This table corresponds to Table S2 by IIP id. Missing values can occur because certain prefectures often do not disclose indices for each industry; where this is the case, we substitute related IIP id as shown in the right column.

Table S6. Production (**x**) and final demand (**y**) at the monthly level in each prefecture (unit: B JPY)

		(1)	(2)	(3)	(4)	(5)	(6)
#	Prefecture	**x**	**x**	**x**	**y**	**y**	**y**
	(Sectors)	All sectors	54 industrial sectors	26 other sectors	All sectors	54 industrial sectors	26 other sectors
1	Hokkaido	2,827	520 (18%)	2,307 (82%)	1,744	206 (12%)	1,538 (88%)
2	Aomori	625	112 (18%)	513 (82%)	413	54 (13%)	359 (87%)
3	Iwate	696	204 (29%)	492 (71%)	438	108 (25%)	330 (75%)
4	Miyagi	1,295	321 (25%)	974 (75%)	775	147 (19%)	628 (81%)
5	Akita	538	123 (23%)	415 (77%)	337	56 (17%)	281 (83%)
6	Yamagata	653	240 (37%)	413 (63%)	415	142 (34%)	273 (66%)
7	Fukushima	1,299	460 (35%)	840 (65%)	739	237 (32%)	502 (68%)
8	Ibaraki	2,099	1,013 (48%)	1,086 (52%)	1,096	398 (36%)	698 (64%)
9	Tochigi	1,412	703 (50%)	709 (50%)	854	393 (46%)	461 (54%)
10	Gunma	1,351	644 (48%)	708 (52%)	758	314 (42%)	443 (58%)
11	Saitama	3,280	1,146 (35%)	2,134 (65%)	1,964	554 (28%)	1,410 (72%)
12	Chiba	3,242	1,116 (34%)	2,127 (66%)	1,785	371 (21%)	1,414 (79%)
13	Tokyo	12,179	879 (7%)	11,300 (93%)	7,148	347 (5%)	6,801 (95%)
14	Kanagawa	5,007	1,741 (35%)	3,265 (65%)	3,050	839 (28%)	2,211 (72%)
15	Niigata	1,401	400 (29%)	1,001 (71%)	832	172 (21%)	659 (79%)
16	Toyama	742	321 (43%)	421 (57%)	394	116 (29%)	279 (71%)
17	Ishikawa	693	194 (28%)	500 (72%)	441	108 (24%)	333 (76%)
18	Fukui	519	164 (32%)	355 (68%)	298	76 (25%)	222 (75%)
19	Yamanashi	528	199 (38%)	329 (62%)	338	114 (34%)	223 (66%)
20	Nagano	1,427	515 (36%)	912 (64%)	872	300 (34%)	572 (66%)
21	Gifu	1,169	450 (39%)	719 (61%)	653	174 (27%)	479 (73%)
22	Shizuoka	2,847	1,354 (48%)	1,493 (52%)	1,543	647 (42%)	896 (58%)
23	Aichi	6,589	3,105 (47%)	3,484 (53%)	3,548	1,441 (41%)	2,106 (59%)
24	Mie	1,488	838 (56%)	651 (44%)	794	373 (47%)	421 (53%)
25	Shiga	970	505 (52%)	465 (48%)	568	250 (44%)	317 (56%)
26	Kyoto	1,367	385 (28%)	982 (72%)	866	208 (24%)	658 (76%)
27	Osaka	5,741	1,351 (24%)	4,390 (76%)	3,134	532 (17%)	2,601 (83%)
28	Hyogo	3,030	1,179 (39%)	1,851 (61%)	1,812	558 (31%)	1,254 (69%)
29	Nara	564	175 (31%)	389 (69%)	379	91 (24%)	288 (76%)
30	Wakayama	576	232 (40%)	345 (60%)	326	93 (29%)	233 (71%)
31	Tottori	307	91 (30%)	216 (70%)	195	44 (22%)	151 (78%)

32	Shimane	377	90 (24%)	287 (76%)	247	47 (19%)	201 (81%)
33	Okayama	1,381	683 (49%)	698 (51%)	731	276 (38%)	455 (62%)
34	Hiroshima	1,989	763 (38%)	1,226 (62%)	1,101	317 (29%)	783 (71%)
35	Yamaguchi	1,045	504 (48%)	541 (52%)	564	214 (38%)	350 (62%)
36	Tokushima	429	139 (32%)	290 (68%)	255	61 (24%)	194 (76%)
37	Kagawa	597	194 (32%)	403 (68%)	318	77 (24%)	242 (76%)
38	Ehime	832	299 (36%)	533 (64%)	470	115 (24%)	355 (76%)
39	Kochi	324	49 (15%)	275 (85%)	218	21 (10%)	197 (90%)
40	Fukuoka	2,844	707 (25%)	2,136 (75%)	1,693	356 (21%)	1,336 (79%)
41	Saga	431	132 (31%)	299 (69%)	266	72 (27%)	194 (73%)
42	Nagasaki	630	120 (19%)	510 (81%)	414	66 (16%)	348 (84%)
43	Kumamoto	840	226 (27%)	614 (73%)	511	92 (18%)	419 (82%)
44	Oita	785	344 (44%)	441 (56%)	416	120 (29%)	296 (71%)
45	Miyazaki	548	123 (22%)	425 (78%)	352	67 (19%)	285 (81%)
46	Kagoshima	796	159 (20%)	637 (80%)	485	71 (15%)	414 (85%)
47	Okinawa	481	49 (10%)	432 (90%)	322	19 (6%)	302 (94%)
--	Monthly total	80,793	25,263 (31%)	55,530 (69%)	46,870	11,455 (24%)	35,415 (76%)
--	Yearly total	969,519	303,157 (31%)	666,362 (69%)	562,437	137,457 (24%)	424,980 (76%)

Note: Monthly values are calculated by dividing annual values by 12.

Table S7. Initial monthly production of each prefecture and initial social, consumer, and producer surpluses (unit: T JPY)

Area	Hyogo (H95Jan)	Niigata (N04Oct) (N07Jul)	Iwate&Miyagi (IM08Jun)	Kumamoto (K16Apr)	FIMIC (GEJE)	Non-FIMIC (GEJE)	Japan
All sectors							
Production (x)	3.0	1.4	2.0	0.8	8.6	72.2	80.8
	(100%)	(100%)	(100%)	(100%)	(100%)	(100%)	(100%)
Consumer surplus	3.8	2.1	2.8	1.2	13.2	90.4	103.6
	(126%)	(150%)	(141%)	(141%)	(153%)	(125%)	(128%)
Producer surplus	1.5	0.7	1.0	0.4	4.3	36.1	40.4
	(50%)	(50%)	(50%)	(50%)	(50%)	(50%)	(50%)
Social surplus	5.3	2.8	3.8	1.6	17.5	126.5	144.0
	(176%)	(200%)	(191%)	(191%)	(203%)	(175%)	(178%)
Mining and Manufacturing sectors (IIP)							
Production (x)	1.2	0.4	0.5	0.2	3.1	22.1	25.3
	(100%)	(100%)	(100%)	(100%)	(100%)	(100%)	(100%)
Consumer surplus	1.4	0.5	0.6	0.2	3.5	23.0	26.6
	(116%)	(119%)	(112%)	(105%)	(114%)	(104%)	(105%)
Producer surplus	0.6	0.2	0.3	0.1	1.6	11.1	12.6
	(50%)	(50%)	(50%)	(50%)	(50%)	(50%)	(50%)
Social surplus	2.0	0.7	0.9	0.4	5.1	34.1	39.2
	(166%)	(169%)	(162%)	(155%)	(164%)	(154%)	(155%)

Table S8. Production (**x**) and final demand (**y**) at the monthly level in 80 sectors (unit: B JPY)

IO id	Sector name	x	y
1	Agriculture, forestry and fishery	1,092	346
2	Metallic ores	2	0
3	Non-metallic ores	73	5
4	Coal mining, crude petroleum and natural gas	10	0
5	Food and Tobacco	2,366	1,599
6	Beverage	617	470
7	Textile products	189	61
8	Wearing apparel and other textile products	191	127
9	Timber and wooden products	211	12
10	Furniture and fixtures	203	54
11	Pulp, paper, paperboard, building paper	373	30
12	Paper products	278	43
13	Publishing, printing	528	27
14	Chemical fertilizer	27	2
15	Basic inorganic chemical products	156	28
16	Basic organic chemical products	220	31
17	Organic chemical products	455	132
18	Synthetic resins	232	61
19	Synthetic fibers	39	13
20	Final chemical products	532	255
21	Medicaments	540	93
22	Petroleum refinery products	1,166	446
23	Coal products	95	9
24	Plastic products	909	151
25	Rubber products	253	89
26	Glass and glass products	144	40
27	Cement and cement products	255	11
28	Pottery, china and earthenware	62	19
29	Other ceramic, stone and clay products	145	36
30	Pig iron and crude steel	567	16
31	steel products	1,125	266
32	Cast and forged steel products	149	9
33	Other iron or steel products	166	8
34	Non-ferrous metals	173	29
35	Non-ferrous metal products	422	88
36	Metal products for construction and architecture	393	24
37	Other metal products	690	127
38	General industrial machinery	777	507
39	Special industrial machinery	1,096	899
40	Other general machines	329	212
41	Machinery for office and service industry	352	274
42	Industrial electric equipment	584	337
43	Applied electrical equipment and electrical measuring instruments	245	220
44	Other electric equipment	312	187
45	Household electric and electric applications	220	193
46	Communication equipment	649	588
47	Electric computing equipment and accessory equipment	345	329
48	Semiconductor devices and integrated circuits	513	270
49	Other electrical equipment	960	307
50	Passenger motor cars	1,070	998

51	Other cars	322	310
52	Motor vehicle parts and accessories	2,371	628
53	Other transportation equipment	450	274
54	Precision instruments	317	255
55	Miscellaneous manufacturing products	394	256
56	Reuse and recycling	73	19
57	Building construction and repair of construction	3,210	2,570
58	Public construction	1,325	1,325
59	Other civil engineering and construction	628	628
60	Electricity	1,497	428
61	Gas supply and heat supply	275	122
62	Water supply and waste management services	719	280
63	Commerce	8,049	5,046
64	Financial and insurance	3,863	1,406
65	Real estate agencies and rental services	1,829	1,190
66	House rent	4,111	4,111
67	Transport	4,118	1,660
68	Communication	1,347	632
69	Broadcasting	297	87
70	Information services	1,520	838
71	Internet-based services	106	20
72	Image information production and distribution industry	608	142
73	Public administration	3,089	2,998
74	Education and Research	3,096	2,079
75	Medical service, health and social security and nursing care	4,616	4,421
76	Advertising and survey	814	181
77	Goods rental and leasing services	995	116
78	Other business services	3,383	583
79	Personal services	4,392	4,161
80	Activities not elsewhere classified	478	27
—	Monthly total	80,793	46,870
—	Yearly total	969,519	562,437

Table S9. Sample size of each IIP for production capacity

IIP id	H95Jan	N04Oct	N07Jul	IM08Jun	K16Apr	GEJE: FIMIC	GEJE: non-FIMIC
(# of prefectures)	(1)	(1)	(1)	(2)	(1)	(5)	(42)
#1	1	1	1	2	1	5	34
#2	1	1	1	2	1	5	38
#3	1	1	1	2	1	5	42
#4	1	1	1	2	1	5	36
#5	1	1	1	2	1	5	41
#6	1	1	0	0	1	4	13
#7	1	1	1	2	1	2	11
#8	0	1	1	2	0	4	26
#9	0	1	1	2	1	5	30
#10	1	1	1	2	1	5	39
#11	1	1	0	0	0	3	4
#12	1	1	1	2	1	5	42
#13	1	1	1	2	1	5	37
#14	1	0	0	1	0	3	13
#15	1	1	1	2	1	5	41
#16	1	1	1	2	0	5	39
#17	1	1	1	2	1	5	41
#18	1	1	1	2	1	5	42
#19	1	1	1	2	0	5	39
#20	1	0	0	0	1	2	23
#21	1	0	0	0	0	0	10
#22	1	1	0	0	0	2	23
#23	1	0	0	0	0	5	19
#24	1	1	1	0	1	5	36
#25	1	1	1	0	1	5	20
#26	0	1	1	1	1	4	26

Notes: This table shows IIP sample sizes for production capacity. IIP data used herein cover 26 sectors and may have missing values, depending on the prefectural statistics. Each of the earthquakes affected a different number of prefectures: 1 prefecture for H95Jan, N04Oct, N07Jul, and K16Apr; 2 prefectures for IM08Jun; and 5 (FIMIC) and 42 prefectures (non-FIMIC) for GEJE. Therefore, the maximum sample size of each IIP id should be 1 for H95Jan, N04Oct, N07Jul, and K16Apr, 2 for IM08Jun, 5 for FIMIC, and 42 for non-FIMIC. Finally, 0 denotes that no IIP data are used.

Table S10. Descriptive statistics for JIP database

Variable	Obs	Average	Std. Dev.	Min	Max
Real gross output (q) (million JPY in 2011)	2,194	9,526,640	11437374	94,677	76,655,035
Nominal gross output (million JPY)	2,194	9,656,146	11498946	110,203	75,516,116
Deflator (p)	2,194	1.047	0.309	0.359	4.780
ln(q)	2,194	15.459	1.182	11.458	18.155
ln(p)	2,194	0.017	0.228	-1.024	1.564

Note: This data comes from the JIP database 2018.

Table S11. Regression results of the price elasticity of demand (ε)

No.	JIP Industry Name	(1) Coef. (ε)	(2) Std. Err.	(3) Real gross output (2005; T JPY)	(4) JIP Industry group (#1–100)	(5) IIP Industry group (#1–80)
1	Agriculture	–0.105	(0.559)	9.7	1–4	1
2	Agricultural services	–1.112	(1.453)	0.7	1–4	1
3	Forestry	1.014*	(0.608)	0.4	1–4	1
4	Fisheries	0.569	(0.750)	1.7	1–4	1
5	Mining	–1.321**	(0.637)	1.2	5–16	2–8,11,12,19
6	Livestock products	–0.337	(0.840)	4.9	5–16	2–8,11,12,19
7	Seafood products	–2.674***	(0.755)	3.7	5–16	2–8,11,12,19
8	Flour and grain mill products	–0.764	(0.645)	1.4	5–16	2–8,11,12,19
9	Miscellaneous foods and related products	1.390	(0.925)	13.1	5–16	2–8,11,12,19
10	Beverages	3.385**	(1.690)	7.8	5–16	2–8,11,12,19
11	Prepared animal foods and organic fertilizers	–0.051	(0.578)	1.0	5–16	2–8,11,12,19
12	Tobacco	–1.358**	(0.574)	3.6	5–16	2–8,11,12,19
13	Textile products (except chemical fibers)	–5.591***	(0.912)	4.6	5–16	2–8,11,12,19
14	Chemical fibers	–3.048***	(0.625)	0.7	5–16	2–8,11,12,19
15	Pulp, paper, and coated and glazed paper	–1.498**	(0.731)	5.3	5–16	2–8,11,12,19
16	Paper products	–1.987**	(0.818)	3.7	5–16	2–8,11,12,19
17	Chemical fertilizers	–0.920	(0.601)	0.7	17–28	14–18, 20-23, 26–29
18	Basic inorganic chemicals	–0.872	(0.596)	1.9	17–28	14–18, 20-23, 26–29
19	Basic organic chemicals	0.359	(0.565)	3.4	17–28	14–18, 20-23, 26–29
20	Organic chemicals	–0.716	(0.580)	12.0	17–28	14–18, 20-23, 26–29
21	Pharmaceutical products	–1.439**	(0.603)	5.7	17–28	14–18, 20-23, 26–29
22	Miscellaneous chemical products	0.062	(1.372)	7.0	17–28	14–18, 20-23, 26–29
23	Petroleum products	–0.195	(0.564)	21.4	17–28	14–18, 20-23, 26–29
24	Coal products	–0.312	(0.564)	2.2	17–28	14–18, 20-23, 26–29
25	Glass and its products	–0.042	(0.613)	1.5	17–28	14–18, 20-23, 26–29
26	Cement and its products	–2.816***	(0.707)	3.5	17–28	14–18, 20-23, 26–29
27	Pottery	0.721	(0.645)	0.7	17–28	14–18, 20-23, 26–29
28	Miscellaneous ceramic, stone and clay products	–0.855	(0.690)	2.0	17–28	14–18, 20-23, 26–29
29	Pig iron and crude steel	0.575	(0.572)	23.6	29–34	30–37

30	Miscellaneous iron and steel	–0.475	(0.581)	10.0	29–34	30–37
31	Smelting and refining of non-ferrous metals	0.129	(0.565)	3.3	29–34	30–37
32	Non-ferrous metal products	–0.068	(0.587)	6.3	29–34	30–37
33	Fabricated constructional and architectural metal products	–2.094***	(0.710)	5.0	29–34	30–37
34	Miscellaneous fabricated metal products	–1.665**	(0.731)	8.3	29–34	30–37
35	General-purpose machinery	–0.275	(1.424)	11.0	35–48	38–49
36	Production machinery	0.087	(1.206)	15.4	35–48	38–49
37	Office and service industry machines	–0.478	(0.590)	4.0	35–48	38–49
38	Miscellaneous business oriented machinery	–0.956	(0.732)	3.6	35–48	38–49
39	Ordnance	–8.378***	(0.832)	0.4	35–48	38–49
40	Semiconductor devices and integrated circuits	–0.717	(0.562)	3.3	35–48	38–49
41	Miscellaneous electronic components and devices	–0.729	(0.569)	9.0	35–48	38–49
42	Electrical devices and parts	0.565	(0.879)	7.5	35–48	38–49
43	Household electric appliances	–0.497	(0.568)	2.3	35–48	38–49
44	Electronic equipment and electric measuring instruments	–0.367	(0.578)	2.3	35–48	38–49
45	Miscellaneous electrical machinery equipment	–0.479	(0.587)	3.1	35–48	38–49
46	Image and audio equipment	–0.181	(0.563)	2.5	35–48	38–49
47	Communication equipment	–0.454	(0.566)	3.7	35–48	38–49
48	Electronic data processing machines, digital and analog computer equipment and accessories	–0.057	(0.561)	2.7	35–48	38–49
49	Motor vehicles (including motor vehicles bodies)	–0.297	(0.835)	23.2	49–51	50–53
50	Motor vehicle parts and accessories	–4.216***	(0.843)	23.6	49–51	50–53
51	Other transportation equipment	0.176	(0.789)	5.4	49–51	50–53
52	Printing	1.814**	(0.776)	5.9	52–59	9,10,13,24,25,54,55
53	Lumber and wood products	–1.841**	(0.731)	2.7	52–59	9,10,13,24,25,54,55
54	Furniture and fixtures	–6.456***	(0.915)	2.5	52–59	9,10,13,24,25,54,55
55	Plastic products	–0.136	(0.894)	11.2	52–59	9,10,13,24,25,54,55
56	Rubber products	–0.928	(0.934)	3.4	52–59	9,10,13,24,25,54,55
57	Leather and leather products	–7.406***	(0.962)	0.5	52–59	9,10,13,24,25,54,55
58	Watches and clocks	1.672***	(0.608)	0.3	52–59	9,10,13,24,25,54,55

59	Miscellaneous manufacturing industries	3.148***	(0.810)	4.1	52–59	9,10,13,24,25,54,55
60	Electricity	–0.333	(0.653)	16.7	60–65	60–62
61	Gas, heat supply	0.621	(0.589)	3.6	60–65	60–62
62	Waterworks	–0.504	(0.735)	3.1	60–65	60–62
63	Water supply for industrial use	–0.721	(0.829)	0.1	60–65	60–62
64	Sewage disposal	1.226	(1.265)	2.6	60–65	60–62
65	Waste disposal	0.424	(0.836)	5.3	60–65	60–62
66	Construction	–1.757	(1.159)	41.5	66,67,89	56–59
67	Civil engineering	–3.584***	(0.950)	24.6	66,67,89	56–59
68	Wholesale	–1.073	(1.354)	76.7	68,69	63
69	Retail	–3.008***	(0.919)	38.1	68,69	63
70	Railway	–0.370	(1.532)	7.6	70–75, 88	67,77
71	Road transportation	0.286	(1.480)	20.5	70–75, 88	67,77
72	Water transportation	1.710**	(0.748)	5.8	70–75, 88	67,77
73	Air transportation	–0.845	(0.695)	4.4	70–75, 88	67,77
74	Other transportation and packing	–1.508	(1.489)	4.3	70–75, 88	67,77
75	Mail	3.066	(2.226)	1.9	70–75, 88	67,77
76	Hotels	1.264	(1.224)	6.5	76,84,85	65,66
77	Eating and drinking services	–2.042**	(0.992)	26.5	77,96–100	79,80
78	Communications	–1.898***	(0.577)	13.8	78–81,87	68–72,76
79	Broadcasting	–4.226***	(1.312)	3.6	78–81,87	68–72,76
80	Information services	–5.958***	(0.826)	18.0	78–81,87	68–72,76
81	Image information, sound information and character information production	4.298**	(2.101)	7.8	78–81,87	68–72,76
82	Finance	0.169	(0.613)	25.3	82,83	64
83	Insurance	–2.426**	(1.183)	12.9	82,83	64
84	Housing	–0.720	(1.365)	45.9	76,84,85	65,66
85	Real estate	–2.321**	(0.941)	20.8	76,84,85	65,66
86	Research	1.378	(1.113)	4.2	86,90–92	73,74,78
87	Advertising	2.857***	(0.964)	8.3	78–81,87	68–72,76
88	Rental of office equipment and goods	–0.725	(0.568)	9.3	70–75, 88	67,77
89	Automobile maintenance services	0.984	(1.077)	10.0	66,67,89	56–59
90	Other services for businesses	–6.552***	(1.078)	29.4	86,90–92	73,74,78
91	Public administration	–1.552	(0.947)	37.3	86,90–92	73,74,78
92	Education	–1.032	(0.883)	21.6	86,90–92	73,74,78

93	Medical service, health and hygiene	–2.722*	(1.629)	33.0	93–95	75
94	Social insurance and social welfare	–0.442	(0.951)	8.0	93–95	75
95	Nursing care	–9.001***	(1.099)	6.2	93–95	75
96	Entertainment	2.071*	(1.150)	10.5	77,96–100	79,80
97	Laundry, beauty and bath services	–0.141	(3.346)	6.3	77,96–100	79,80
98	Other services for individuals	3.075**	(1.333)	7.9	77,96–100	79,80
99	Membership organizations	–1.337	(1.326)	4.9	77,96–100	79,80
100	Activities not elsewhere classified	–0.844	(0.677)	3.5	77,96–100	79,80
—	Constant (for #1)	16.149***	(0.032)	—	—	—
—	Industry dummy (#2–100)	Yes	—	—	—	—
—	# of observations	2,194	—	—	—	—
—	R-squared	0.989	—	—	—	—
—	Adjusted R-squared	0.988	—	—	—	—

Notes: Columns 1 and 2 show the estimated results of a regression model. Values with and without parentheses are coefficients and standard error, respectively. ***, **, and * denote statistically significant levels of 1%, 5%, and 10%, respectively. Column 3 shows real gross output as of 2005 to calculate weighted average price elasticity in 17 summarized groups (see Table II). Columns 4 and 5 indicate the JIP Industry group (#1–100) and IIP Industry group (#1–80), respectively, for creating the weighted average groups.

Table S12. Earthquake damage estimates from previous studies: H95Jan and GEJE

#	Estimation	Direct damage	Indirect damage	(This study)
H95Jan	Hyogo prefectural government and National Land Agency, Japan[27]	9,926.8B JPY for damage to capital stock	—	(The cumulative loss of social surplus [Δss] is 1.25T JPY for 12 months [Δcs is 1.45T JPY; Δps is –0.2T JPY])
H95Jan	Toyoda and Kouchi (1997)	13,268.2B (=9,926.8B+3,341.4B) JPY in total; 5,930B JPY in total in 10 cities and 10 towns; 1,510B JPY for industrial sectors in 10 cities and 10 towns.	7,230B JPY in total in 10 cities and 10 towns (for 1 year); 1,203.1B JPY for industrial sectors in 10 cities and 10 towns (for 1 year).	
GEJE	The national government (Hayashi, 2012)	Approximately 16,900B JPY (or 3.5% of GDP)	—	(The cumulative loss of social surplus [Δss] is 16.94T JPY until the temporal recovery [7.83T JPY to FIMIC at t=37; 9.11T JPY to non-FIMIC prefectures at t=8])
GEJE	Hayashi (2012)	Approximately 30T JPY (or 6% of GDP)	Approximately 10T JPY for the annual gross regional product in Fukushima, and 100T JPY in total for 10 years. This study estimates approximately 24T JPY for 10 years only in industrial sectors.	
